# Identification of reliable reference genes for quantitative real-time PCR normalization in pitaya

**DOI:** 10.1186/s13007-019-0455-3

**Published:** 2019-07-08

**Authors:** Canbin Chen, Jingyu Wu, Qingzhu Hua, Noemi Tel-Zur, Fangfang Xie, Zhike Zhang, Jianye Chen, Rong Zhang, Guibing Hu, Jietang Zhao, Yonghua Qin

**Affiliations:** 1 State Key Laboratory for Conservation and Utilization of Subtropical Agro-bioresources/Guangdong Provincial Key Laboratory of Postharvest Science of Fruits and Vegetables/Key Laboratory of South China Horticultural Crop Biology and Germplasm Enhancement, Ministry of Agriculture, College of Horticulture, South China Agricultural University, Guangzhou, 510642 China; 2 French Associates Institute for Agriculture and Biotechnology of Drylands, The J. Blaustein Institutes for Desert Research, Ben-Gurion University of the Negev, 84990 Sede Boqer, Israel

**Keywords:** *Hylocereus*, Reference genes, Identification, qRT-PCR, Normalization, *Cytochrome P450* gene

## Abstract

**Background:**

A suitable reference gene is an important prerequisite for guarantying accurate and reliable results in quantitative real-time PCR (qRT-PCR) analyses. However, there is no absolute universality in reference genes among different species. It’s hard to find an ideal reference gene to fit for different tissues and growth periods. Pitaya (*Hylocereus*) is commercially produced as a new fruit crop at a large scale in tropical and subtropical regions. To date, there is no report on the identification of the most reliable reference genes for qRT-PCR normalization in pitaya.

**Results:**

In this study, six candidate reference genes i.e. *Actin(1)*, *GAPDH*, *UBC(1)*, *UBC(2) EF1*-*α(1)* and *histone(1)* were selected from thirty-nine typical candidate reference genes to determine the most stable reference genes for qRT-PCR normalization in different tissues, temperature stresses and fruit developmental stages of pitaya. Among the six candidate reference genes, *Actin(1)* and *EF1*-*α(1)* were the most stable gene according to calculations of three statistical methods (GeNorm, NormFinder and BestKeeper) while *UBC(1)* and *UBC(2)* showed the lowest expression stability. The six candidate reference genes were further validated by comparing expression profiles of key genes related to betalain biosynthesis at flesh coloration stages of Guanhuahong (*Hylocereus monacanthus*) and Guanhuabai (*H. undatus*) pitayas. *Actin(1)* was recommended the best reference gene for accurate normalization of qRT-PCR data.

**Conclusions:**

In this study, the stability of the selected reference genes for normalizing the qRT-PCR data were identified from pitaya. *Actin(1)* was the most stably expressed genes in different tissues and fruit developmental stages in pitaya. The present work provides the first data of reference gene identification for pitaya and will facilitate further studies in molecular biology and gene function on *Hylocereus* and other closely related species.

**Electronic supplementary material:**

The online version of this article (10.1186/s13007-019-0455-3) contains supplementary material, which is available to authorized users.

## Background

Quantitative real-time PCR (qRT-PCR) is a nucleic acid quantitative technology to study gene expression level in molecular biology research [1]. It combines the sensitivity of conventional PCR with a cost-effective assay using a specific fluorescent signal. Compared with northern blot and conventional RT-PCR, qRT-PCR is proven to be highly sensitive and specific and has been increasingly used in various research fields of biological sciences [2–4]. A stable reference gene plays an important role in analyzing the relative expression levels of target genes [5]. Reference gene, i.e. internal reference gene, whose expression is relatively constant in various tissues and cells, can be used as the reference in the detection of gene relative expression analyses. However, transcription levels of reference gene may change in different species, treatments and/or developmental stages. Therefore, it is necessary to validate the expression stability of reference genes under various tissues and experimental conditions in different species.

The accuracy of gene expression levels using qRT-PCR depends on the use of stable reference genes to normalize the difference between samples [6]. *GAPDH*, *Actin*, *18S rRNA*, *28S rRNA*, *tubulin*, *EF1*-*α* and *ubiquitin* are commonly used as reference genes for comparison of samples [7–9]. Reference genes are always expressed because they produce proteins that cells must have to function, however, the stability of reference genes is limited in different tissues, growth and development stages of the same or different plants [10–15]. Therefore, it is necessary to select the most suitable reference gene for accurate qRT-PCR evaluation according to different species and experimental conditions [16, 17]. In addition, calculations of reference genes by geNorm [6], NormFinder [18] and BestKeeper [19] is essential for normalization of qRT-PCR analyses.

Pitaya or pitahaya, also known as dragon fruit, originated from rainforest in tropical and subtropical areas of Central America and Mexico. Pitaya belongs to the genus *Hylocereus* in the Cactaceae family. Currently, red-flesh and white-flesh pitayas have been commercially produced as a new fruit crop at a large scale in Central America, Southeast Asia and China. Pitaya is a fast growing, perennial, hemi-epiphytic, vine-like, crassulacean acid metabolism (CAM) plant. It is a spiny succulent plant which can adapt to a wide ecological range such environmental cues as drought, heat and poor soil. Pitaya has gained great attention of the world due to its high nutritional value as well as its antioxidant capacity and antiproliferative activities [20–22]. Therefore, the knowledge about the molecular basis of pitaya may contribute to the discovery of new and promising genes related to important agricultural traits that could be further transferred to a target crop for the development of new crop cultivars.

Gene expression analysis is an important tool to elucidate the complex regulatory networks of the genetic, signalling and metabolic pathway mechanisms during plant life cycle. qRT-PCR is an ideal tool to verify the results of differential gene expression of interest on a smaller scale. To study mRNA expression levels, a set of appropriate reference genes are fundamental to get reliable results from qRT-PCR. In this study, thirty-nine genes annotated as *18S rRNA* (*18S ribosomal RNA*), *CYP* (*cytochrome*), *Actin*, *GAPDH* (*glyceraldehyde*-*3*-*phosphate dehydrogenase*), *EF1*-*α* (*eukaryotic elongation factor 1*-*alpha*), *histone*, *TUA* (*alpha*-*tubulin*), *TUB* (*beta*-*tubulin*), *UBQ* (*ubiquitin*) and *UBC* (*ubiquitin*-*conjugating enzyme*) were selected as candidate reference genes to evaluate their expression stability in different pitaya tissues, temperature stresses and fruit developmental stages using three available statistical algorithms i.e. geNorm [6], NormFinder [18] and BestKeeper [19]. The aim of the present study is to identify appropriate reference genes for qRT-PCR normalization in pitaya and facilitate future molecular studies in *Hylocereus*. In addition, we validated the expression levels of the key genes related to betalain biosynthesis using the selected reference genes.

## Materials and methods

### Plant materials

Two pitaya cultivars i.e. Guanhuahong (*H. monacanthus*) and Guanhuabai (*H. undatus*) pitayas from the same orchard of Dalingshan Forest Park were used as plant materials. Guanhuahong pitaya is a red flesh fruit with red peel while Guanhuabai pitaya is a white flesh fruit with red peel (Additional file 1: Fig. S1). Root, stem, receptacle, calyx, petal, filament, anther, ovary, style, stigma, peel and flesh were collected separately and used for expression analyses of candidate reference genes. Pitaya flesh were collected on the 13rd, 16th, 19th, 23rd, 25th, 27th and 29th days after flowering (DAF) in July and August, 2015 (Additional file 1: Fig. S1). Cutting seedlings were collected for expression analyses of candidate reference genes on the 0, 9th, 18th, 36th, 72th and 144th h after cultivating in different temperature stresses (4 °C, 10 °C and 25 °C). All samples were immediately frozen in liquid nitrogen and stored at − 80 °C until use.

### Candidate gene selection and primer design

Thirty-nine candidate reference genes with relatively stable expression levels (based on reported RPKM and fold change values) i.e. *18S rRNA, Actin(1)*, *EF1*-α*(1)*, *EF1*-α*(2)*, *EF1*-α*(3)*, *EF1*-α*(4)*, *EF1*-α*(5)*, *GAPDH*, *Actin(2), Actin(3), Actin(4)*, *TUA(1)*, *TUA (2)*, *CYP(1)*, *CYP(2)*, *eIF(1)*, *eIF(2)*, *eIF(3)*, *eIF(4)*, *eIF(5)*, *histone(1), histone(2), histone(3), histone(4), histone(5), TATA(1), TATA(2), TATA(3), UBC(1)*, *UBC(2)*, *UBC(3)*, *UBC(4)*, *UBC(5)*, *UBC(6)*, *TUB(1)*, *TUB(2)*, *UBQ(1)*, *UBQ(2)* and *UBQ(3)* were selected from pitaya transcriptome database (NCBI accessions: SRR2924904) (Additional file 2: Table S1) [23]. The specific primers (Table 1) were designed using Primer Premier 5.0 with the following parameters: melting temperature (Tm) values ranging from 47 to 63 °C, GC percent of 25–65%, primer lengths of 18–22 bp and product length of 100–300 bp. Tenfold serial dilutions of cDNA were used to determine slope of the standard curve to calculate amplification efficiency of primer (E = 10^(−1/slope of the standard curve)^).

**Table 1:** Primers used in this study

Genes	Forward primer (5′-3′)	Reverse primer (5′-3′)	Length (bp)	Efficiency (%)
*18S rRNA*	TGCTTTGAGCACTCTAATTT	CTTGGGTCGTAAGGGTCGGT	140	90.1
*Actin(1)*	AAAGGCTAACAGGGAGAAAA	GACCACTGGCGTAAAGAGAA	104	107.9
*Actin(2)*	AAACTAGGAAGGAGAAAGGA	AAATAGAGAGCATACAGGGC	230	94.3
*Actin(3)*	GCTTTTCCTTGATGTCTCTC	TCCTGCCATGTATGTTGCTA	260	103.4
*Actin(4)*	GAAAACGGATGGGAGGAGAA	GCTGATAGAATGAGCAAGGA	238	94.9
*CYP(1)*	GTGCCCAAAACAGCAGAAAA	ATCCCCCAGAGTGAAATCCC	150	95.9
*CYP(2)*	GGGTTCAGTAGGAGGGATTT	CTCAGCAGTTTTCGGGGTAA	140	106.3
*EF1*-*α(1)*	CGAAGGCACAGAAATACCGT	GCTTTTTACCCATCCAAATG	288	108.5
*EF1*-*α(2)*	AGGTTCTCCACTCAGGCAAC	ACACTCCGCACAATCTCTTC	260	99.4
*EF1*-*α(3)*	TCACACGGGAAGAGGGAAAG	AGGGTATAGCAGCCAGGAAC	290	96.3
*EF1*-*α(4)*	GAGCACTCTTTCTCTTTTGA	GAATCTGGTTTAGTCCCTGT	286	97.9
*EF1*-*α(5)*	AACAGGGGGAAAGGGAAAAC	TGGGGAGGAGAGGAAGGGTA	220	110.4
*eIF(1)*	TCTTCTATGGATGTATGGGG	CGTTCTTGGTGTTGAGGTTT	114	100.9
*eIF(2)*	CAGTGTCTTTTGTTCCGCCT	TTCCTGTGCCCCTACTTGCC	168	99.5
*eIF(3)*	AGGGATGGGAAGAAGGTAAC	ATTCAAAGCATTCAGGGTCA	192	95.3
*eIF(4)*	TCCTTAGCCTGGGATTCTTC	CCTGGTTTCTGAGTTTGCGT	236	99.7
*eIF(5)*	CTCAGAGACTCAGACCCACA	GACCATCCAATGAACCACCT	218	98.6
*GAPDH*	GGTATGAGCAGAATAAAGCA	GGTAATCAATAACAGAACGC	124	96.7
*histone(1)*	AGTCACATACACTGAGCACG	CATAACTAAACGAAGAACCA	228	108.3
*histone(2)*	GGACATTTGATTAGGTTTTG	ATAGATTCTCCCTTTTTTTT	192	95.9
*histone(3)*	TGTCTTTTGATTTGGTGTTT	CTCTCCATTTTCTTTTTGTG	170	92.1
*histone(4)*	GAAAAGTGCTCCGAGACAAC	AACCCCCAAATCCATACAAA	252	96.7
*histone(5)*	TCTCTGGAGCATGTAGAAGC	GGCAAAAACCCATAGACTGT	226	91.3
*TATA(1)*	CATAAAGGAAGGGAGGGAGA	ATGATTACAGCAGCGAAACG	224	97.5
*TATA(2)*	GTTAAATTTCCCATCAGACT	CCAGATACAAAGATAAGCAG	134	99.9
*TATA(3)*	TTCTGCTACATCTGCTCTCT	TTACATTTCTTTCTGCTTCC	246	101.3
*TUA(1)*	AAGGAAGATGCGGCAAATAA	GGAGGGAGTGCGTAGAGAGC	298	106.4
*TUA(2)*	AGAAGGTGTTAAACGAATCA	AGGAAGAGAAAAAGAGGTAG	180	98.1
*TUB(1)*	ATTACTCGGTTACTCCAGCC	AAAGATTCCCCTCTTCAAAA	102	97.4
*TUB(2)*	GGTGGGAGGAGGTGGTGGTA	AGGGAGGGATTCTGAGCCGT	158	93.7
*UBC(1)*	TCTCGGGATAATCTTTGTCA	AGGAACTTCAGGTTGTTGGA	196	105.4
*UBC(2)*	TTTCCATCTGCTCGCTGTTG	GCTCCTTGCTGTGCTCTCGT	110	102.1
*UBC(3)*	TATTCTTTGACTTTTCGCTC	TTCCTGATGCTTATCCCTAC	262	96.9
*UBC(4)*	TGGCTTCCAGACAAATCAAC	GCAACCTACCTCAGCACCTT	300	93.5
*UBC(5)*	TATCAATCCTTCATCCGCCA	CCCTTCCTTATCATCCCTCC	182	99.5
*UBC(6)*	GAAGTTGTCGGAGCTTGGTG	TTTTGGAGGTGAATGAGGGA	288	99.6
*UBQ(1)*	TCGGAAAGGAAAGTCAATTA	CTCACCCACATCAAACACAG	256	105.7
*UBQ(2)*	AATAGACTCAAAATAAGGCG	CCAAGAAAATGGAAGAACTG	296	106.8
*UBQ(3)*	TCAACCAAATAACCCCTGCG	CCTGCCTTGTGGCTCTCACC	284	108.1
*Cyt P450*-*like1*	GCTCCAGCCGAACCATACCC	TCTTCCTAAAACTCCGCCAT	102	104.3

### RNA extraction and cDNA synthesis

Total RNA was extracted according to the protocol of Wu et al. [24] and was purified by RNase-free columns (Huayueyang, Beijing, China). Genomic DNA was removed by DNase I (TaKaRa, Dalian, China). RNA quality was analyzed by 1.0% agarose gel and RNA integrity was assessed using an Agilent 2100 Bioanalyzer (Agilent Technologies, Santa Clara, CA, USA). Only RNA samples with an A260/A280 ratio of 1.8–2.2 and an A260/A230 ratio > 1.8 were used for further analysis. cDNA was synthesized from 2.0 µg of total RNA samples using M-MLV first strand cDNA synthesis kit according to the manufacturer’s instructions (TaKaRa).

### qRT-PCR analyses

qRT-PCR was conducted with ABI 7500 real-time PCR System (Applied Biosystems, CA, USA) using the SYBR^®^ Premix Ex *Taq*™ II (TaKaRa). Twenty microliters reaction mixture contained 2 µL of diluted cDNAs (~ 15 ng/μL), 10 µL SYBR Premix Ex *Taq* II (TaKaRa), 0.4 µL of each primer (10 µM) and 7.2 µL ddH_2_O. The PCR reaction conditions were as follows: 50 °C for 2 min; 95 °C for 10 min; 40 cycles at 95 °C for 15 s; 56 °C for 30 s; and 72 °C for 40 s. The melting curve was generated by heating the amplicon from 60 to 95 °C to confirm primer specificity. Each PCR reaction was repeated three times with three biological replicates. Relative fold changes in gene expression were calculated using the comparative 2^−ΔΔCT^ method [25].

### Analysis of gene expression stability

NormFinder, geNorm and BestKeeper were used to evaluate expression stability of reference genes in various pitaya tissues, temperature stresses and fruit developmental stages. The geNorm program determines stability and the optimal number of genes required to calculate the M value and pairwise variation Vn/Vn + 1 between two sequential normalization factors [6]. The NormFinder program was performed to obtain the best candidate reference genes by the average expression stability between samples based on the results of variance analysis [18]. The BestKeeper program is used for determination of stable reference genes, which calculates pairwise correlations based on standard deviation (SD) and percentage covariance (Cov) values [19].

### Validation of reference genes

To validate the reliability of the selected reference genes, the relative expression levels of a key gene *Cytochrome P450* (*Cyt P450*-*like1*) involved in betalain biosynthesis were analyzed using qRT-PCR at different flesh coloration stages of Guanhuahong and Guanhuabai pitayas. The specific primers were designed according to Hua et al. [23] (Table 1).

### Statistical analyses

Statistical analyses were performed using SPSS 17.0 software.

## Results

### Selection of candidate reference genes and sequence analyses

Thirty-nine reference genes from the transcriptome of pitaya were selected as putative candidate reference genes (Additional file 2: Table S1). The cDNA fragments of the 39 reference genes ranged from 234 to 5304 bp. Results from BLAST analyses demonstrated that 27 reference genes had maximum identity (80–92%) with similar deduced polypeptides from *Chenopodium quinoa*, 7 reference genes had maximum identity (93 to 99%) with similar deduced polypeptides from *H. monacanthus*, and the rest are similar to the other plants (Additional file 3: Table S2). For example, *Actin(1)* had 98% sequence identity with *Hpactin7* (MF356257.1), *eIF(1)* shared 86% sequence identity with *CqeIF6*-*2* (XM_021866570.1) and *histone(3)* showed 92% sequence identity with *Sghistone* (KC415067.1) (Additional file 3: Table S2).

### Verification of primer specificity and stability

Melting curve plays an important role in assessing the effects of primer specificity. As shown in Additional file 4: Fig. S2, there were only one distinctive peak for *Actin(1)*, *EF1*-α*(1)*, *EF1*-α*(2)*, *EF1*-α*(3)*, *EF1*-α*(5)*, *GAPDH*, *Actin(3)*, *TUA(1)*, *TUA(2)*, *CYP(1)*, *CYP(2)*, *eIF(1)*, *eIF(2)*, *eIF(3)*, *eIF(4)*, *eIF(5)*, *histone(1)*, *histone(3)*, *histone(4)*, *histone(5)*, *TATA(1)*, *TATA(2)*, *TATA(3)*, *UBQ (1)*, *UBQ (2)*, *UBQ (3)*, *UBC(1)*, *UBC(2)*, *UBC(3)*, *UBC(4)*, *UBC(5)*, *UBC(6)*, *TUB(1)* and *TUB(2)*. Therefore, those thirty-four candidate reference genes were selected from the thirty-nine candidate reference genes according to the melting curves (Additional file 4: Fig. S2). The efficiency for each primer pair varied from 90.1% for *18S rRNA* to 110.4% for *EF1*-*α(5)* (Table 1). The sequence length ranged from 102 to 300 bp (Table 1). Cycle threshold (Ct) values ranged from 17.45 for *GAPDH* to 37 for *histone(2)*. The boxplot provided expression levels of the 39 candidate reference genes (Additional file 5: Fig. S3). A lower Ct value indicated a higher gene expression levels. Across all samples, the maximum Ct values of *Actin(1)*, *EF1*-α*(1)*, *UBC(1)*, *UBC(2)*, *GAPDH* and *histone(1)* were < 23. *GAPDH* expression levels were the most variable with 5.4 Ct, while *Actin(1)* showed the least variable levels with 1.5 Ct. Based on the melting curve and Ct values (Additional file 4: Fig. S2 and Additional file 5: S3), six genes i.e. *Actin(1)*, *EF1*-*α(1)*, *GAPDH*, *UBC(1)*, *UBC(2)* and *histone(1)* were selected as candidate reference genes for further analyses.

### Ct value analyses of six reference genes

**Fig. 1:**
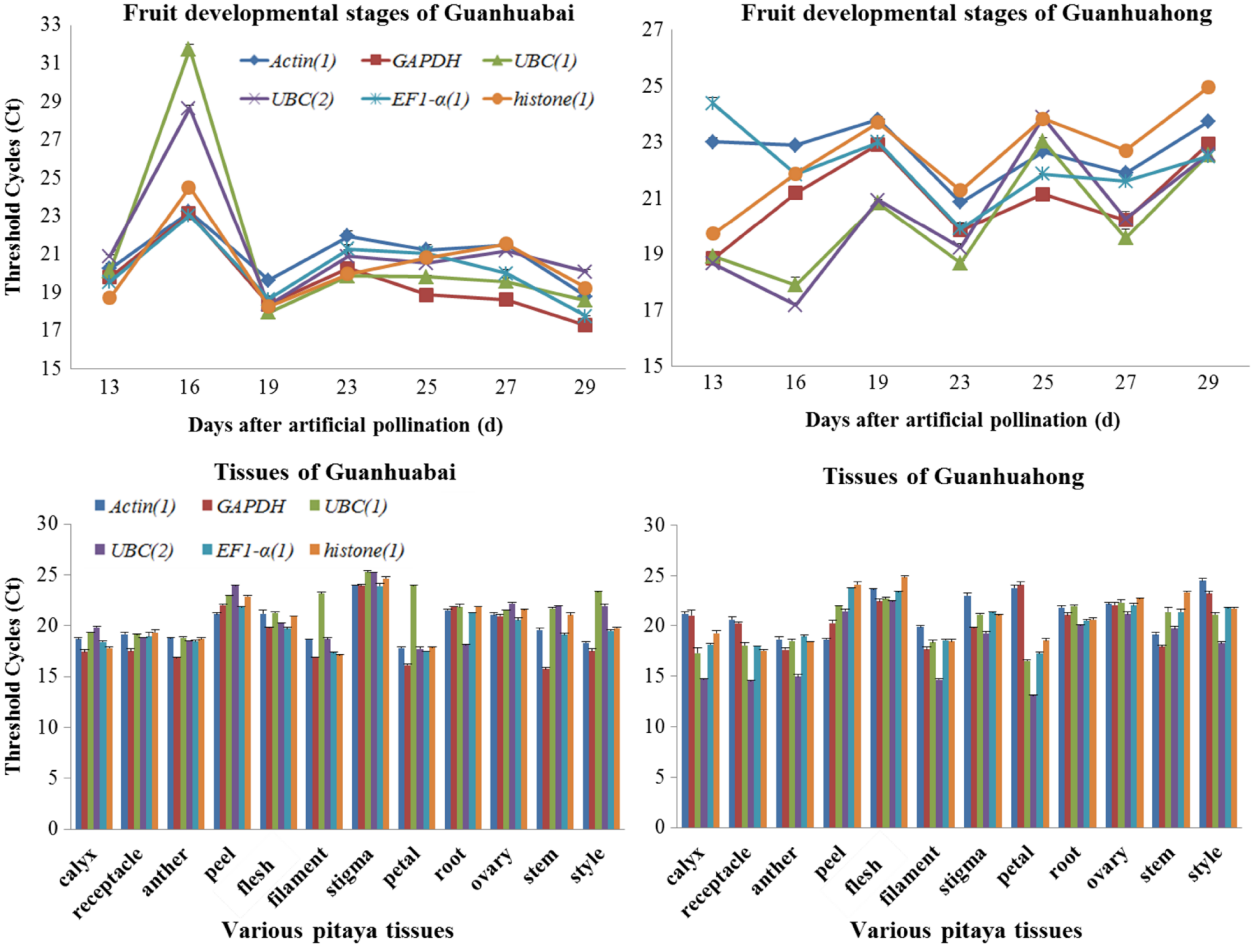
Ct values of six candidate reference genes in different fruit developmental stages and tissues of pitayas. Data represent the average values from three biological replicates (± SD)

Ct values of *Actin(1)*, *EF1*-*α(1)*, *GAPDH*, *UBC(1)*, *UBC(2)* and *histone(1)* were calculated to determine their transcript levels in different tissues, temperature stresses, fruit developmental stages of Guanhuahong and Guanhuabai pitayas. As shown in Fig. 1, a relatively wide range of transcript levels for the six reference genes were detected in different tissues, temperature stresses, fruit developmental stages of the two different color-fleshed pitaya cultivars. In different fruit developmental stages, Ct values of Guanhuabai and Guanhuahong pitayas ranged from 17.28 to 31.74 and 17.18 to 24.94, respectively. Average Ct values were 21.82 for *Actin(1)*, 21.18 for *EF1*-α*(1)*, 20.25 for *GAPDH*, 20.66 for *UBC(1)*, 20.96 for *UBC(2)* and 21.52 for *histone(1)*, respectively (Fig. 1). The lowest Ct value was 17.28 for *GAPDH* and the highest Ct value was 31.74 for *UBC(1)* in different fruit developmental stages of Guanhuabai pitaya. As for different fruit developmental stages of Guanhuahong pitaya, the lowest Ct value was 17.18 for *UBC(2)* and the highest Ct value was 24.94 for *histone(1)*.

Ct values ranged from 15.76 to 25.29 and 14.53 to 24.82 in different tissues of Guanhuabai and Guanhuahong pitayas, respectively. Average Ct values were 20.70 for *Actin(1)*, 20.06 for *EF1*-α*(1)*, 19.75 for *GAPDH*, 20.98 for *UBC(1)*, 19.23 for *UBC(2)* and 20.59 for *histone(1)*, respectively (Fig. 1). The lowest Ct value was 15.76 for *GAPDH* gene and the highest Ct value was 25.29 for *UBC(2)* in different tissues of Guanhuabai pitaya. The lowest Ct value was 14.53 for *UBC(2)* gene and the highest Ct value was 24.82 for *histone(1)* gene in different tissues of Guanhuahong pitaya.

In different temperature stresses, the lowest Ct value was 15.92 for *GAPDH* and the highest Ct value was 23.74 for *histone(1)*. Ct values ranged from 20.81 to 22.84 for *Actin(1)*, 17.79 to 19.78 for *EF1*-α*(1)*, 15.92 to 19.82 for *GAPDH*, 19.50 to 21.35 for *UBC(1)*, 18.13 to 19.69 for *UBC(2)* and 19.53 to 23.74 for *histone(1)*, respectively (Additional file 6: Fig. S4).

The coefficient of variance (CV) of Ct values can determine reference gene stability levels. *UBC(2)* presented a high degree of transcript level variation with a CV value of 15.61% while *Actin(1)* presented the lowest degree of variation with a CV value of 4.96% (Additional file 7: Table S3). Among the six candidate reference genes, *Actin(1)* (4.96%) was the most stable gene while *UBC(1)* (13.16%) and *UBC(2)* (15.61%) showed the lowest expression stability according to CV value analyses of six reference genes.

### GeNorm analysis

**Fig. 2:**
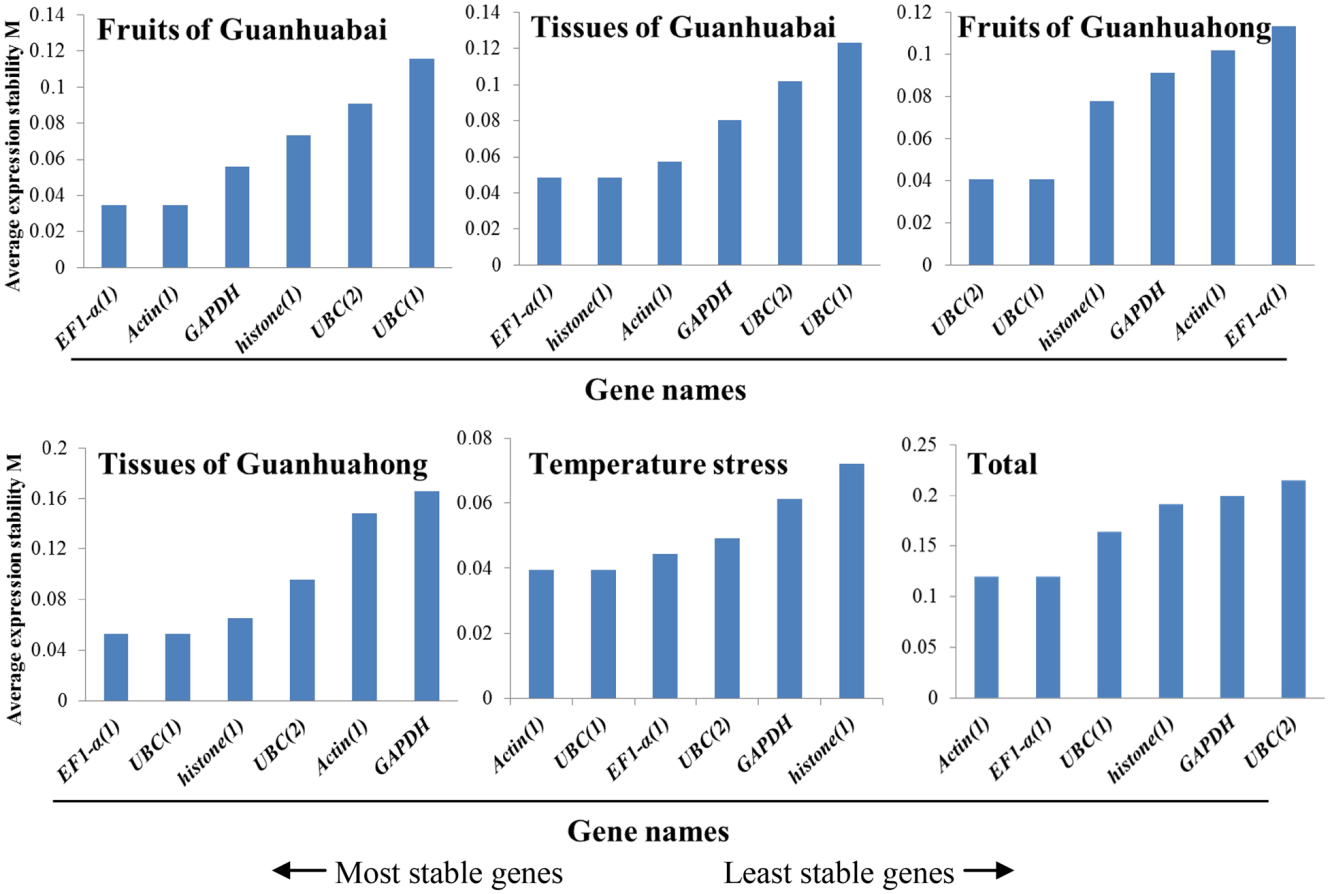
Average expression stability values (M) of the six candidate reference genes using geNorm algorithm. Total represents a comparison between all tissues/fruits between the two cultivars

GeNorm was used to select the most stable reference gene by calculating the gene expression stability measure (M) based on the average pairwise expression ratio. The gene with the lowest M value is considered the most stable. A gene with M value below 1.5 can be considered as a reference gene. As shown in Fig. 2, the M values of *Actin(1)*, *EF1*-*α(1)*, *GAPDH*, *UBC(1)*, *UBC(2)* and *histone(1)* were lower than 1.5, suggesting that all of them conformed to basic requirements for the reference gene. Among the six genes, *UBC(2)* was the least stable reference gene with the highest M value (> 0.214) detected in the two different colored pitayas, different tissues and fruit developmental stages of Guanhuahong and Guanhuabai pitayas. *EF1*-*α(1)* and *Actin(1)* were the most stable genes with the lowest M value (< 0.058) in different tissues and fruit developmental stages of Guanhuabai pitayas. As for different development stages of Guanhuahong pitaya, the most stable genes were *UBC(1)* (M = 0.041) and *UBC(2)* (M = 0.041), followed by *histone(1)* (M = 0.078). *UBC(1)* (M = 0.053) and *EF1*-*α(1)* (M = 0.053) showed the highest levels of expression stability for different tissues of Guanhuahong pitaya. In different temperature stresses, the most stable genes were *Actin(1)* (M = 0.040) and *UBC(1)* (M = 0.040). *Actin(1)* (M = 0.119) and *EF1*-*α(1)* (M = 0.119) are the most stable reference gene suitable for transcript normalization in all tissues/fruits between the two cultivars. Concerning all samples of tested, *Actin(1)* was the best reference gene for qRT-PCR normalization.

**Fig. 3:**
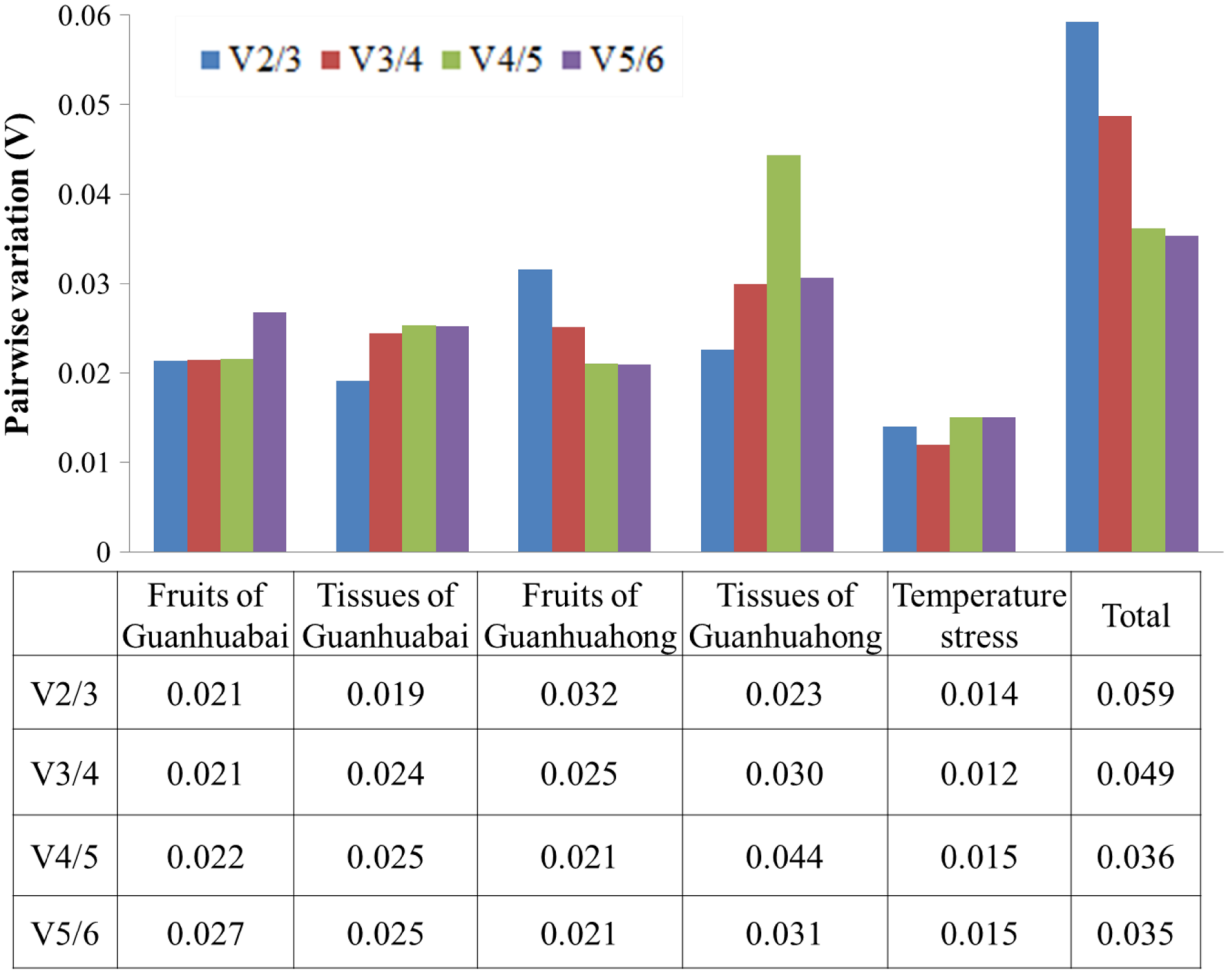
Pairwise variation (V) analyses of the six candidate reference genes using geNorm algorithm. Total represents a comparison between all tissues/fruits between the two cultivars

A single reference gene often can not meet the requirements of accurate quantification for transcription analyses of gene expression. It is necessary to use two or more than two reference genes to produce accurate and reliable normalization. The optimal number of reference genes can be determined using the pairwise variation value (Vn/Vn + 1, V-value) as normalization factor for reference genes. Additional (n + 1) reference genes are necessary to normalize the genes if V-value is higher than the threshold of 0.15. As shown in Fig. 3, the pairwise variation values of reference genes were examined when all samples were considered. The ratio of V2/V3 was lower than the threshold of 0.15, suggesting that the optimum number of reference gene was two. There is no need to add a third reference gene as an internal control for the normalization of gene expression in pitaya. Therefore, a combination of *Actin(1)* and *EF1*-*α(1)* was the optimum reference genes for qRT-PCR normalization of various samples in pitaya.

### NormFinder analysis

**Fig. 4:**
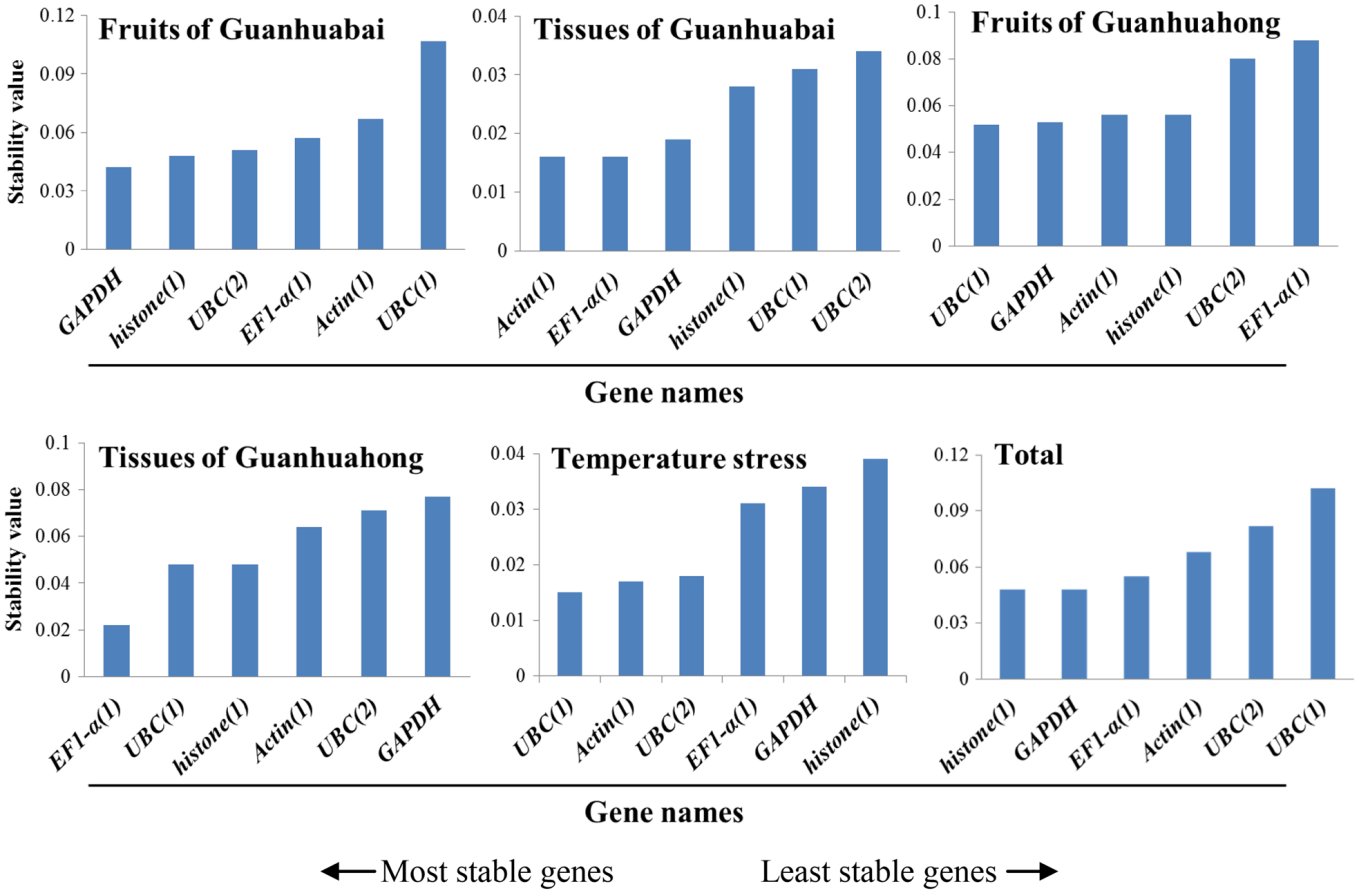
Expression stability values and ranking of the six candidate reference genes using NormFinder algorithm. Total represents a comparison between all tissues/fruits between the two cultivars

Different from geNorm, the NormFinder can determinate optimal number of reference genes for normalization by intra- and inter- group variations. Average gene expression stability values of the six reference genes in different samples were calculated by NormFinder software and ranking was made based on relative stability values. As shown in Fig. 4, *UBC(1)* was the least stable reference gene in the total dataset, which is different from the result obtained by NormFinder. In different fruit developmental stages, *GAPDH* and *histone(1)* emerged as the most stably expressed gene for Guanhuabai pitaya compared with *UBC(1)* and *GAPDH* for Guanhuahong pitayas. In various tissues of Guanhuabai and Guanhuahong pitayas, *EF1*-*α(1)* was the most stable reference gene. *UBC(1)* and *Actin(1)* were the best reference gene for different temperature stresses. Combined with expression analyses of all samples, *histone(1)* and *GAPDH* were the most stable reference genes for normalization in pitaya.

### BestKeeper analysis

**Fig. 5:**
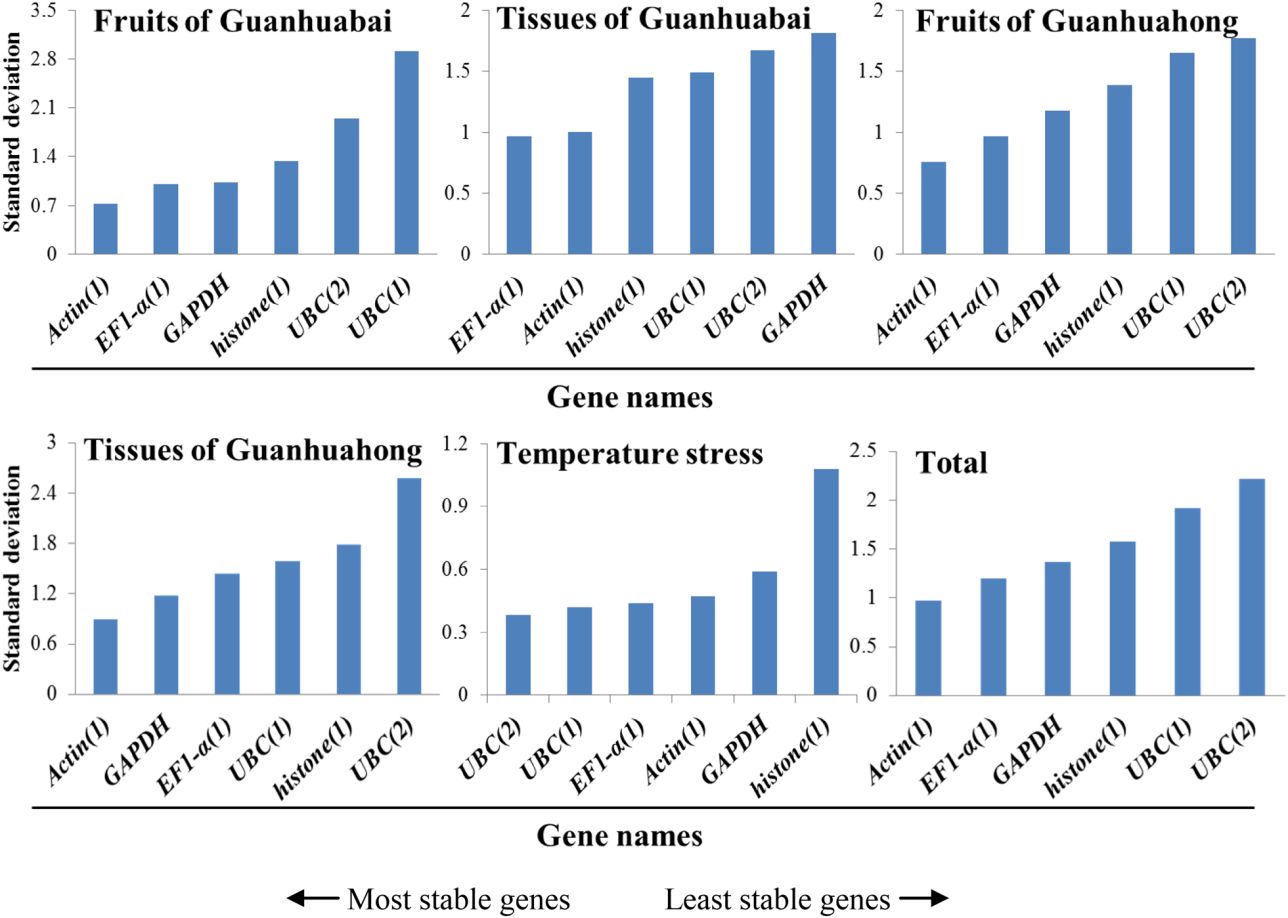
Expression stability values and ranking of the six candidate reference genes using BestKeeper algorithm. Total represents a comparison between all tissues/fruits between the two cultivars

BestKeeper program was used to grade candidate reference gene stability by calculating SD of the Ct values, CV and pair correlation coefficient. A gene with SD value below 1.0 can be considered as stable expression. In different temperature stresses, SD values were 0.38 for *UBC(2)*, 0.42 for *UBC(1)*, 0.44 for *EF1*-*α(1)*, 0.47 for *Actin(1)*, 0.59 for *GAPDH* and 1.08 for *histone(1)*, respectively. As for the different fruit developmental stages of Guanhuahong pitaya, SD values of *Actin(1)* and *EF1*-*α(1)* were 0.76 and 0.97, respectively, suggesting *Actin(1)* and *EF1*-*α(1)* could be used as reference genes. *Actin(1)* was the most stably expressed gene, followed by *EF1*-*α(1)* (Fig. 5). Compared with Guanhuabai pitaya, *Actin(1)* was the most stable gene for normalization of the different fruit developmental stages of Guanhuahong pitaya. In different tissues of Guanhuabai and Guanhuahong pitayas, *EF1*-*α(1)* (0.97) and *Actin(1)* (0.9) was the most stable gene, respectively; *UBC(2)* (2.58) was the least stable reference gene with the highest SD value. In the different colored pitayas, *Actin(1)* (0.97) was considered to be the most stable gene. Taking into account the total dataset, *Actin(1)* was the most stable gene in all samples tested. The results of BestKeeper were consistent with the analyses of geNorm.

### Reference genes validation

**Fig. 6:**
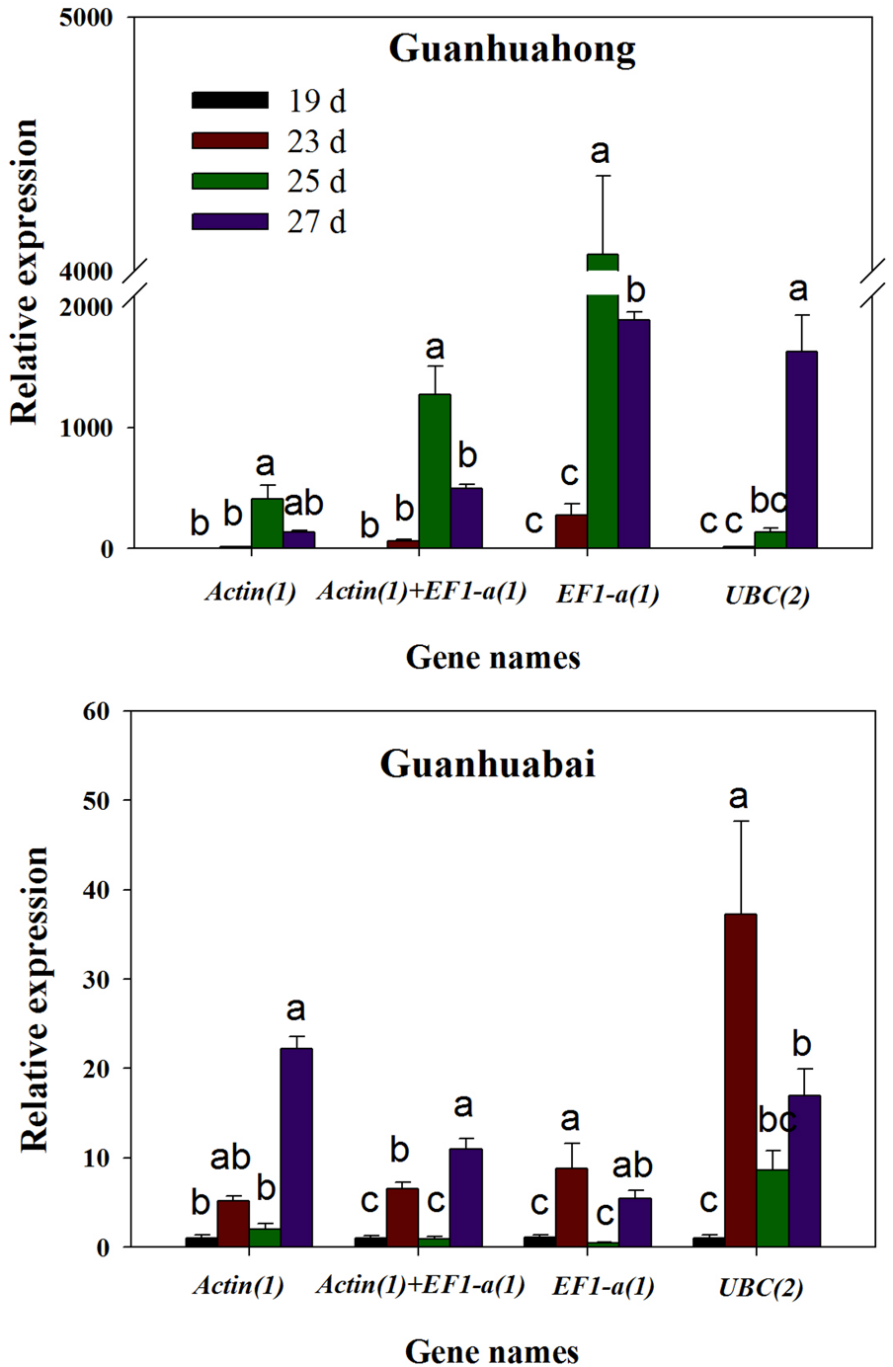
Relative expression of *cyt P450*-*like1* in Guanhuahong and Guanhuabai pitayas. *Actin(1)*, *EF1*-*α(1)* and *Actin(1)* + *EF1*-*α(1)* were used as one or two most stable reference genes, *UBC(2)* was used as the least stable reference gene. Different letters indicate significant difference of the expression of the target gene based on three biological replications (*P* < 0.05, t test; n = 3)

To validate the suitability of the two selected reference genes (*Actin(1)* and *EF1*-*α(1)*), and the least suitably ranked candidates *UBC(2)* according to BestKeeper and geNorm analyses. Relative expression levels of a key gene *Cyt P450*-*like1* involved in betalain biosynthesis were evaluated using qRT-PCR at different flesh coloration stages of Guanhuahong (*H. polyrhizus*) and Guanhuabai (*H. undatu*s) pitayas (Fig. 6). The expression levels of *Cyt P450*-*like1* increased gradually during flesh color transition from white (23 days) to red stages (25 days) and decreased at full maturation stage (29 days) when single or a combination of reference genes (*Actin(1)* and *EF1*-*α(1)*) were used for normalization. These results were in accordance with betalain accumulation pattern and expression characteristics of *Cyt P450*-*like1* gene related to betalain biosynthesis at all flesh coloration stages of *H. monacanthus* [23, 26]. However, the expression pattern of *Cyt P450*-*like1* normalized by *UBC(2)* increased gradually at all flesh coloration stages of Guanhuahong pitaya. Furthermore, when *UBC(2)* was used to normalize *Cyt P450*-*like1* expression in Guanhuabai pitaya, the expression values of 23 days were overestimated. In general, the RT-qPCR profiling of *Cyt P450*-*like1* gene expression supported the analyses of BestKeeper and geNorm .

## Discussion

qRT-PCR is the most sensitive technology of measuring gene transcript levels depending on stable reference gene(s) for normalization [27]. An appropriate reference gene can effectively correct the errors of RNA quantity, reverse transcription efficiency, stability of different reaction channels of PCR instrument and standard operation mode, which can help to obtain the real differential expression of the target genes [28]. The reference genes such as *Actin*, *18S rRNA* and *GAPDH* are commonly used as reference genes. However, these reference genes show instability in various plant species or genotypes [13, 14, 29]. Their expression levels are different in different patterns of cells and tissues, physiological status and growth periods [30, 31]. Therefore, it is essential to select the most suitable reference gene for the specific sample types and experimental conditions. Pitaya has become a popular fruit due to its excellent nutritional, commercial and medical values [20–22]. To date, there is no report on the identification of the most suitable reference gene in pitaya. In this study, 39 typical reference genes from pitaya transcriptome database [23] were selected to determine the most stable reference gene for qRT-PCR normalization in pitaya. Based on the melting curve and Ct values of reference genes (Additional file 4: Fig. S2 and Additional file 5: S3), six genes, i.e. *Actin(1)*, *EF1*-*α(1)*, *GAPDH*, *UBC(1)*, *UBC(2)* and *histone(1)* were selected as candidate reference genes for further analyses.

NormFinder [18], geNorm [6] and BestKeeper [19] were commonly used to evaluate the stability of the reference genes. The geNorm was applied to estimate the stability of a candidate gene by pairwise comparison, while the NormFinder and BestKeeper were used to prevent co-regulation and to further assess the results obtained by the geNorm program. In the present study, geNorm, NormFinder and BestKeeper were applied to define the stability of *Actin(1)*, *EF1*-*α(1)*, *GAPDH*, *UBC(1)*, *UBC(2)* and *histone(1)* reference genes in different tissues, temperature stresses and fruit developmental stages of pitaya. Different rankings for the six candidate reference genes were detected after comparison to the ranking of the candidates generated by the three algorithms (Fig. 2, 3, 4, 5). From the geNorm evaluation, we found that *Actin(1)* and *EF1*-*α(1)* were the optimum reference genes for qRT-PCR normalization of various samples of pitaya. The results of BestKeeper were consistent with the analyses of geNorm, showed that *Actin(1)* was the most stable reference gene in all of the samples examined. However, results from NormFinder analyses showed that *histone(1)* and *GAPDH* were the most stable reference genes in all of the samples tested. The reason for the differences in the top-ranked reference genes may be due to the discrepancies resulted from different approaches of calculations by geNorm, NormFinder and BestKeeper programs. Similar results were also obtained in *Vitis vinifera* [32], *Citrus* [33], *Euscaphis konishii* [34], Cowpea [35] and *Actinidia deliciosa* [36]. It has been reported that the most discrepant results in gene stability ranking were obtained by BestKeeper [37]. In our study, although there are some differences in the stability of each reference gene expression of samples from the three different algorithms, *Actin(1)* and *EF1*-*α(1)* are the optimum reference genes. Combining the three algorithms with expression analyses, we suggested *Actin(1)* was the most adequate reference gene for expression studies in different tissues, temperature stresses and fruit developmental stages of pitaya.

*Cytochrome P450* is a key gene involved in betalain biosynthesis [23, 38–42]. To validate the candidate reference genes, the relative expression levels of *Cyt P450*-*like1* gene were analyzed by qRT-PCR during flesh coloration of Guanhuabai and Guanhuahong pitayas. Expression levels of *Cyt P450*-*like1* increased gradually during flesh coloration and decreased at full maturation stage when single or a combination of reference genes (*Actin(1)* and *EF1*-*α(1)*) were used as reference genes. Those results were in consistent with betalain accumulation pattern and expression characteristics of *Cyt P450*-*like1* at all flesh coloration stages of *H. monacanthus*. Moreover, the expression pattern of *Cyt P450*-*like1* increased with fruit maturation of *H. undatus* [23, 24]. Those results suggested that *Actin(1)* was the most stable reference gene for expression studies of key genes involved in betalain biosynthesis of pitaya. And the most stable reference gene combination for expression studies in pitaya was *Actin(1)* and *EF1*-*α(1)*.

## Conclusions

To the best of our knowledge, this study is the first report on systematically evaluating the expression stability of different potential reference genes for qRT-PCR in the Cactaceae family. Thirty-nine typical reference genes were selected to determine the most stable reference genes for qRT-PCR normalization in pitaya. Based on the melting curve and Ct values, *Actin(1)*, *EF1*-*α(1)*, *GAPDH*, *UBC(1)*, *UBC(2)* and *histone(1)* genes were selected for further analyses in different tissues, temperature stresses and fruit developmental stages of pitayas. *Actin(1)* and *EF1*-*α(1)* were identified as the optimum internal control genes according to calculations made with geNorm, NormFinder and BestKeeper programs. Validation of suitable reference genes was carried out to profile the expression of *Cyt P450*-*like1* gene during flesh coloration stages of Guanhuahong and Guanhuabai. *Actin(1)* was the best reference gene for qRT-PCR normalization. The present study provides the appropriate reference gene for normalization of reliable qRT-PCR data in different pitaya tissues, temperature stresses and fruit developmental stages which will be useful for the expression profiles of target genes in *Hylocereus* plant and related species.

## Additional files

**Additional file 1: Fig. S1.** Different fruit developmental stages of Guanhuabai (A) and Guanhuahong (B) pitayas. A1 and B1, 13 days; A2 and B2, 16 days; A3 and B3, 19 days; A4 and B4, 23 days; A5 and B5, 25 days; A6 and B6, 27 days; A7 and B7, 29 days. Bar = 4.0 cm.

**Additional file 2: Table S1.** cDNA sequences of thirty-nine reference genes.

**Additional file 3: Table S2.** Sequence analyses of thirty-nine reference genes.

**Additional file 4: Fig. S2.** Melt curve analyses of thirty-nine reference genes from eight different tissues (including roots, stems, flowers, and fruits) of *Hylocereus.*

**Additional file 5: Fig. S3.** Boxplot analyses of thirty-nine reference genes from eight different tissues (including roots, stems, flowers and fruits) of *Hylocereus.* The whisker caps show the distribution of the highest and lowest Ct values. The boxes indicate the first and third quartile, while the middle line marks the median.

**Additional file 6: Fig. S4.** Ct value of six candidate reference genes in different temperature stresses of pitaya cutting plantlets.

**Additional file 7: Table S3.** The coefficient of variance (CV) of the six candidate reference genes using BestKeeper algorithm.

## Data Availability

The data and materials supporting the conclusions of this study are included within the article.

## Abbreviations

CAM: crassulacean acid metabolism
cDNA: complementary DNA
Cov: covariance
Ct: cycle threshold
CV: coefficient of variance
DNase I: deoxyribonuclease I
EF1-α: eukaryotic elongation factor 1-alpha
eIF: eukaryotic initiation factor
GAPDH: glyceraldehyde-3-phosphate dehydrogenase
SD: standard deviation
TATA: TATA box binding protein-associated factor
TUA: alpha-tubulin
TUB: beta-tubulin
UBC: ubiquitin-conjugating enzyme
UBQ: ubiquitin
