# Supplementary material for: Identification of reliable reference genes for quantitative real-time PCR normalization in pitaya
Source: Plant Methods. 2019 Jul 8;15:70. doi: 10.1186/s13007-019-0455-3 (PMC6613322; doi:10.1186/s13007-019-0455-3)
Supplement: Supplementary file 1 — Additional file 1: Fig. S1. Different fruit developmental stages of Guanhuabai (A) and Guanhuahong (B) pitayas. A1 and B1, 13 days; A2 and B2, 16 days; A3 and B3, 19 days; A4 and B4, 23 days; A5 and B5, 25 days; A6 and B6, 27 days; A7 and B7, 29 days. Bar = 4.0 cm. [file 13007_2019_455_MOESM1_ESM.doc]

**
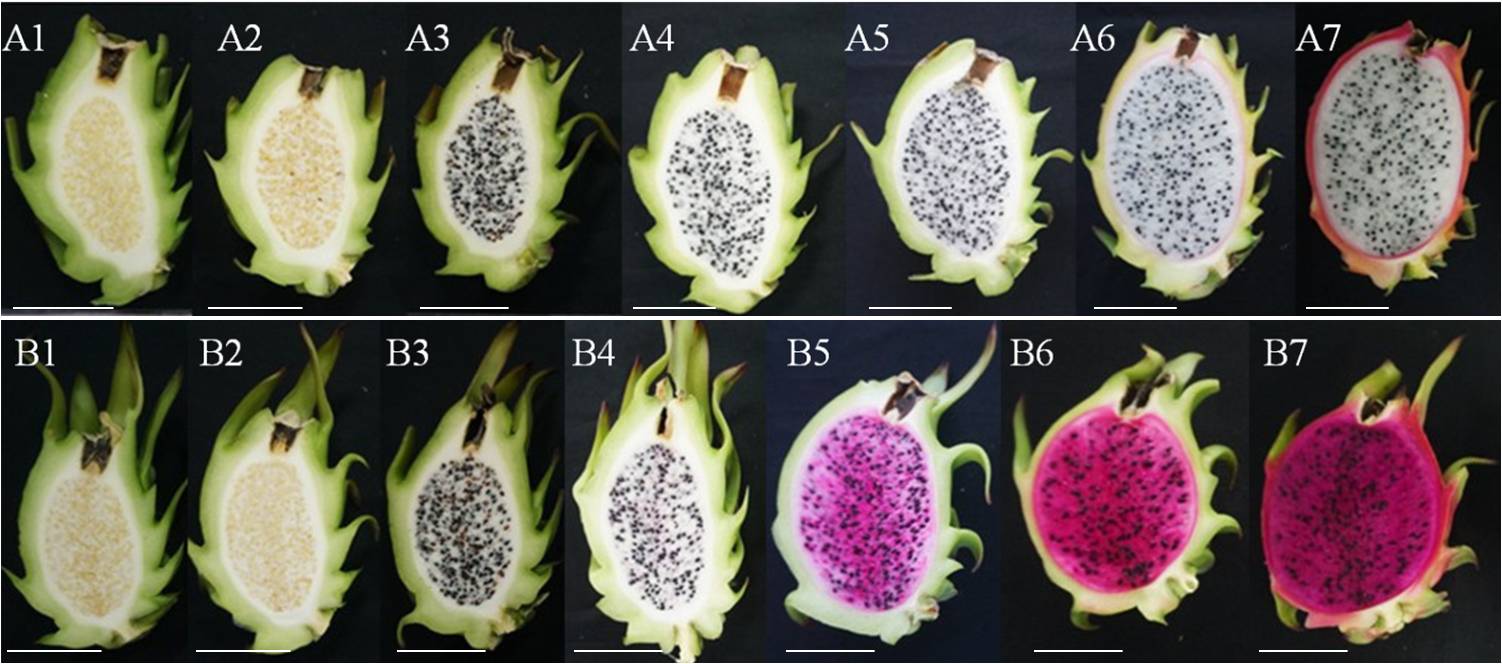
Additional file 1: Fig. S1. Different fruit developmental stages of Guanhuabai (A) and Guanhuahong (B) pitayas.** A1 and B1, 13 d; A2 and B2, 16 d; A3 and B3, 19 d; A4 and B4, 23 d; A5 and B5, 25 d; A6 and B6, 27 d; A7 and B7, 29 d. Bar=4.0 cm.
