# Supplementary material for: Identification of reliable reference genes for quantitative real-time PCR normalization in pitaya
Source: Plant Methods. 2019 Jul 8;15:70. doi: 10.1186/s13007-019-0455-3 (PMC6613322; doi:10.1186/s13007-019-0455-3)
Supplement: Supplementary file 2 — Additional file 2: Table S1. cDNA sequences of thirty-nine reference genes. [file 13007_2019_455_MOESM2_ESM.doc]

**Additional file 2: Table S1 cDNA sequences of 39 reference genes.**

*18S rRNA*

CCGAAGGCCAACACAATAGGACCAGACTCCTATCACGTTATTCCATGCTAATGTATTCAGAGCATAAGCTTGCTTTGAGCACTCTAATTTTTTCAAAGTAACAGCGCCGGAACCGCGACCCAGCCAATTAAGGCCAGGAGCACGCCGCCGACAAAAGGGACGGAAGGGCAGGTGCACACCGAGTAGGCGGACCGACCCTTACGACCCAAGATCCAACTACGAGCTTTTTAACTG

*Actin(1)*

CACCCCCCGCTCAACAATCAACATAATAAAGCTAGCAAGTCTGCTCGTTTAAACGCCTCTCTCTCTCTCTGTCTCTCTCACTCCCTCGCGTACACACTTTCTCGTCTCTCCTCTCGCAGCCTCAAGTTCTTCGCTCTTCAAGTATAAACTAAGATGGCTGACGCAGAGGAGATTCAACCCCTTGTCTGTGACAATGGAACTGGAATGGTCAAGGCTGGGTTTGCTGGGGATGACGCTCCAAGGGCAGTCTTCCCAAGTATTGTTGGTCGTCCCAGACACACTGGTGTCATGGTTGGCATGGGCCAGAAGGATGCATATGTTGGAGATGAAGCTCAATCTAAAAGAGGTATTTTGACATTGAAGTACCCAATTGAGCATGGTATTGTGAGCAACTGGGATGATATGGAGAAGATTTGGCACCACACCTTCTACAACGAGCTTCGTGTTGCTCCCGAAGAGCACCCAGTTTTGCTTACTGAGGCACCCTTGAATCCAAAGGCTAACAGGGAGAAAATGACTCAGATTATGTTTGAGACCTTCAATGTTCCTGCCATGTACGTTGCCATTCAGGCCGTCCTTTCTCTTTACGCCAGTGGTCGTACTACAGGTATTGTGCTCGATTCTGGTGATGGTGTTAGTCACACGGTTCCCATCTATGAGGGTTATGCCCTTCCTCATGCCATCCTCCGTTTGGATCTTGCTGGGCGTGATCTGACTGATTATCTCATGAAGATCCTCACTGAGAGAGGTTACATGTTCACCACATCTGCCGAGCGGGAAATTGTCCGTGATATCAAGGAGAAGCTTGCCTATGTCGCTCTTGACTTTGAGCAGGAATCAGAGACTGCCAAGAGCAGCTCTGCAGTTGAGAAGAACTATGAACTTCCTGATGGTCAAGTGATTACCATTGGAGCGGAGAGATTCCGTTGCCCAGAGGTCCTCTTCCAGCCTTCATTGATTGGTATGGAAGCTGCAGGTATCCATGAGACAACGTACAACTCCATCATGAAGTGTGATGTTGATATCAGAAAGGACCTCTATGGTAACATTGTGCTCAGTGGTGGTACTACCATGTTCCCTGG

*Actin(2)*

ATACAGTGTCTGGATTGGAGGATCCATCTTAGCATCTCTCAGCACATTCCAGCAGATGTGGATTGCGAAGGCAGAGTATGACGAATCTGGTCCGTCAATTGTCCACCGCAAGTGCTTCTAAGATCATCCCATAGGATGAAAGTGGTCGAAGAAGAATGATCTTCCCACTTGGACAAAGAAAAATGCAGCCTTGCAGATCTTTATATGATCGAGTTCTTATACCGTCATTGTTGTCAAAATCGGCCTTTCAAAACTAGGAAGGAGAAAGGATTTAAACGCTAAAGAGAAAAAGAATTAAATTCATTCATTATTTCGCTTTTTTGTTAGTTTAATTGACACTTTGTTACAATGATAATTGACAGTAATATTTGGTGTTGTGATGTACGAGTGAGAATCCAGATATTCTATAATTACCAATTTTTCATTCTTTGTTCATGAGAAAGATAAAAACATGACGTTTGCCCTGTATGCTCTCTATTTGG

*Actin(3)*

TTCCTTGCTCATTCTGTCAGCAATACCCGGGAACATAGTTGTACCACCACTGAGGACAATGTTTCCATATAGATCCTTCCTAATATCCACATCGCATTTCATGATTGAGTTATAAGTAGTCTCATGGATACCAGCTGCTTCCATTCCAATGAAAGATGGCTGGAAAAGGACCTCTGGGCAACGGAAGCGCTCAGCTCCAATAGTGATGACTTGTCCATCAGGCAGCTCATAGTTCTTCTCGACTGCAGAGCTAGTCTTGGTGGTTTCTAGTTCCTGGTCATAGTCAAGGGCAATGTAGGCAAGCTTTTCCTTGATGTCTCTCACAATTTCACGCTCAGCTGATGTAGTGAAAGAATACCCACGTTCTGTAAGAATCTTCATCAGGTAATCTGTCAGGTCACGGCCAGCAAGGTCAAGACGGAGGATAGCATGTGGGAGGGCATATCCTTCATAGATGGGAACTGTGTGGCTGACACCATCACCGGAGTCCAGCACAATACCTGTCGTACGACCACTTGCATATAGGGAAAGCACAGCCTGGATAGCAACATACATGGCAGGAGTGTTAAATGTCTCAAACATTATTTGAGTCATCTTTTCACGGTTAGCCTTAGGATTGAGAGGAGCTTCAGTAAGCAAAACTGGATGCTCCTCTGGAGCTACACGGAGCTCATTGTAGAAGGTGTGATGCCATATCTTTTCCATGTCATCCCAGTTG

*Actin(4)*

ATGGTATTCAAAACATGGTGTAAAAATGTCGATGTGATTTATCAAACTTCATGAATGAATTCATTTATCAAAGAATAAACGCAATTCTTAAACCAGTCCTACATCATTACAATTTAAGAATTGACACAGAAAAAAACCCAAGAAAAAGAAAGGGAAAAAAGGAAGACAAAAGAAACGACAATATGATGGGCATCTTTCTGTACTTGAAAGCAAACAACAGCTCCTGCACCGAAAGGGATTTTGAACCCATTTCCCCCGCCGGGGGTTTTGGGGGGGGGGGGGTTAAGAAAACGGATGGGAGGAGAAAAAGGAAAGAAACAAACTTAGAAGCACTTTCTATGGACAATTGACGGCCCAGATTCGTCATACTCGGCCTTGGCAATCCACATCTGTTGGAATGTACTAAGGGATGCCAAGATGGAACCTCCGATCCAAACACTGTATTTTCTCTCGGGTGGTGCCACCACCTTAATCTTCATACTGCTCGGGGCCAAGGCTGTAATTTCCTTGCTCATTCTATCAGCAATTCCAGGGAACATAGTTGTACCCCCACTAAGTACAATGTTCCCATAGAGGTCCTTCCTGATATCCACGTCACACTTCATGATAGAGTTATACGTAGTTTCATGGATACCTGCAGCTTCCATTCCAATGAAAGACGGCTGGAAGAGAACCTCTGGACATCTGAACCTCTCGGCTCCAATCGTGATAACCTGCCCATCAGGCAGCTCATAGGTCTTCTCAACTGCAGAACTGGTTTTGGCGGTCTCCATCTCTTGATCATAGTCAAGGGCAATGTAGGCA

*CYP(1)*

ATTATGGGCCTGGAGGGTTTTAGAGCGAGAAAGATGGCAAGAACGACGTCAATTACGGTGGCTTTCATCTGGATTCTTGCTATTTTCGGAACCCTAGTTCAAGTTCAGGGTAAGAAGAAATCGAAGGAGAATCTGAAGGAAGTGACGCACAAGGTGTTCTTTGACATTGAGATCGATGGAAAACCAGCTGGTCGGATTATTATGGGTCTCTTTGGCAAGACAGTGCCCAAAACAGCAGAAAATTTCAGAGCATTGTGCACTGGTGAAAAAGGCATTGGCAAATCCGGGAAACCCTTGCATTACAAGGGAAGCACATTCCACAGGATTATCCCCAGCTTCATGATCCAAGGCGGGGATTTCACTCTGGGGGATGGAAGAGGTGGAGAATCAATCTACGGGTGGAAGTTTGATGATGAAAATTTCAAGCTGAAGCATACCGGACCTGGGCTTCTGTCCATGGCCAATGCAGGTCCAAACACCAATGGTTCTCAGTTCTTCA

*CYP(2)*

CGGCTTTGCCATCGTTGCTCTTCGAAACCCCTCTGCATCACAAACCACAAGCTCGCCGAGAACTCAACACAAGGTGAAGGGTTCAGTAGGAGGGATTTTGAAGAGCAACGATGGCAAAGCCGAACCCCAAAGTTTTCTTTGACATTGTCATTGGGCAAATGAAGGCTGGAAGAGTTGTTATGGAGCTATTTGCTGATGTTACCCCGAAAACTGCTGAGAATTTCCGTGCTCTGTGCACTGGAGAGAAAGGAATTGGAAAAGTCGGGAAGCCACTCCACTACAAGGGCTCGGCATTTCATCGGATTATCCCCAACTTCATGTGCCAAGGTGGGGACTTCACAAGGGGCAATGGCACCAGAGGAGAATCAATTTATGGTGCGAAGTTTAATGATGAGAACTTCAAGTTGAAGCATACAGGGCCTGGTATTCTATCAATGGCCAATGCTGGTCCCAATACGAATGGCTCGCAGTTCTTCATATGCACCACTAAGACACCATGGCTGGATGGCAAGCATGTGGTCTTTGGGAAAGTAGTAGATGGTTATAAGGTGGTCGAACAGATGGAGGCAGTGGGCAGTGAGAGTGGGAGAACTACTGAGCCTGTGGTCATTGAAGACTCTGGTCAATTGCCTGATAACTGATGAGACCGGAAGATCAGAGGAGCTTTCTTCGAGGGAATGGGTGTTGGTTTTATGATGGGAAGAGACGTAGATTAGTCTTTTATTTCTATTGTAAGTGTACTTCAAACCCTGGGTGAAAGTTGT

*EF1-α(1)*

TTGGAGTCGCATTTTTTTATCTAAATACACTTTGCATGAGTTAAAGAGTTGGCCATGGGAAAATATCTTTGGCCTTCGGCCGCGGGAATTAAAAATGTCGGAATATTTCTATTCAAAATATCCGAATATCAGCAGTATTAGGGTTTCCCTTTCTGTCTCTCGTTTCTCACTCCTTGATCACGATTAAAACCCTCTTCGTAGTCCTCAGCCGCACCTCTCTTGTTCTCCTCGTTTCTCTTTCTCTCTCCTCCGCATCACTTTCCCTCTTCACTTTTTGATTGGTGACGAAACCTCTCGCCGATTGAGCTCAGTCTCAGGGTTCGTTAATATCCTTCTTGATCCTATCGGGGAGTGCCTGTTTTTTCTAAAGAGCTGCCAAGATGGTGAAGTTCACAGCTGATGAGCTCCGCAAAATTATGGATCTTAAACATAACATCCGTAACATGTCTGTCATTGCCCATGTCGATCATGGAAAGTCTACACTGACCGACTCATTGGTGGCTGCTGCTGGTATTATCGCCCAGGAAGTGGCTGGTGACGTGCGAATGACAGACACCCGTGCTGACGAAGCTGAACGTGGTATTACCATCAAGTCCACTGGAATTTCTCTCTACTATGAGATGACTGATGAAGCTCTGAAGGCTTACAAGGGAGAGAGACTGGGCAATGAGTACCTCATCAACCTTATTGATTCCCCTGGGCACGTTGACTTCTCGTCAGAGGTCACAGCTGCTCTGCGTATCACTGATGGTGCCCTTGTCGTGGTTGACTGTATTGAGGGAGTTTGTGTCCAGACAGAAACTGTCCTTCGGCAGGCTCTTGGTGAGAGGATCAGGCCTGTCCTGACTGTTAACAAGATGGACCGTTGCTTCCTTGAGCTTCAGGTTGATGGTGAGGAGGCTTACACGACATTCCAGAAGGTAATTGAGAATGCTAATGTCATTATGGCTACATATGAAGATCCCCTTCTGGGAGATGTTCAAGTCTATCCTGAGAAAGGAACCGTTGCTTTCTCAGCGGGTCTCCATGGTTGGGCATTTACTCTGTCCAACTTCGCTAAGATGTATGCCTCCAAGTTTGGAGTTGATGAGGCTAAGATGATGGAGCGCCTTTGGGGTGAGAACTTCTTTGACCCAGCCACTAAGAAGTGGACCACCAAGAACACCGGTTCCCCAACTTGTAAGCGTGGATTTGTCCAGTTCTGTTATGAGCCCATCAAGCAGATCATCTCAACCTGCATGAATGACCAAAAGGACAAGCTGTGGCCTATGTTGCAAAAACTCGGTGTCCAATTGAAGGCTGAAGAGAAAGACTTGATGGGGAAGGCATTGATGAAGCGTGTCATGCAGACCTGGCTGCCAGCTGCTAGTGCATTGCTGGAAATGATGGTCTATCACTTGCCATCTCCGGCGAAGGCACAGAAATACCGTGTTGAGAACTTGTATGAAGGACCTATGGATGACCCTTATGCCACGGCCATTAGGAACTGTGATCCGGAGGGACCTTTGATGCTTTATGTCTCAAAGATGATTCCTGCATCCGACAAGGGTAGATTCTTTGCCTTTGGTCGTGTTTTCTCCGGAAAGGTTTCTACCGGTATGAAGGTTAGAATCATGGGTCCTAACTACGTGCCTGGAGAGAAGAAGGATCTCTATGTCAAGAACGTTCAGAGAACTGTCATTTGGATGGGTAAAAAGCAAGAGACAGTGGAGGATGTGCCATGCGGAAACACTGTTGCAATGGTTGGTTTGGATCAGTACATCACCAAAAATGCAACCCTTACGAACGAGAAGGAAGTTGATGCCCACCCAATCCGTGCTATGAAGTTTTCTGTCTCCCCCGTAGTCCGTGTTGCTGTGCAGTGTAAGGTTGCCTCTGACCTTCCCAAGCTCGTTGAAGGTTTGAAGCGTCTAGCCAAATCAGACCCTATGGTTGTCTGTTCCATTGAGGAGTCTGGTGAGCACATCATTGCTGGTGCTGGAGAACTTCACTTGGAAATCTGTTTGAAGGATCTGCAGGAGGACTTTATGGGTGGGGCTGAGATCATCAAGTCTGACCCTGTTGTCTCCTTCCGTGAGACTGTTCTTGAGAGGTCATGCCGTACTGTGATGAGCAAGTCTCCCAACAAGCACAACCGTTTGTACATGGAGGCACGTCCGATGGAGGAAGGGCTTGCCGAGGCTATTGATGACGGCCGTATTGGTCCAAGAGATGACCCCAAGAACAGATCGAAGATCTTAGCTGAGGAGTTTGGCTGGGACAAGGATCTTGCGAAGAAAATCTGGTGTTTTGGGCCAGAAACTACAGGTCCCAACATGGTTGTTGATATGTGTAAGGGAGTCCAGTACCTGAATGAAATCAAGGATTCTGTTGTTGCCGGTTTCCAATGGGCATCAAAAGAAGGTGCCTTGTCTGAAGAGAACATGAGAGGCATCTGTTTTGAGGTGTGCGATGTGGTTCTTCATGCTGATGCCATCCATCGTGGTGGTGGTCAGATTATTCCCACTGCTAGGAGGGTGTTCTACGCTTCCCAGCTGACTGCCAAACCCAGGTTGATGGAGCCCGTCTACCTCGTGGAAATCCAGGCTCCCGAGCAAGCTCTTGGTGGTATCTACAGTGTTCTTAACCAGAAGCGTGGTCATGTGTTTGAAGAAATGCAGAGGCCTGGTACTCCTCTCTACAACATCAAGGCATACCTCCCCGTCATTGAGTCCTTTGGTTTCTCGAGTACATTGAGGGCTGCAACATCTGGTCAAGCTTTCCCACAGTGTGTGTTTGATCACTGGGACATGCTGTCGTCAGATCCCTTGGAGCCCGGAACCCAAGCTGCTGCTATTGTGGCAGATATCCGTAAGAGGAAGGGTTTGAAGGAGCAGATGACCCCACTGTCCGAGTATGAGGACAAGCTGTAAGCTTTTCCGTCTCTATGGAACAAAATTCATTTTGGTAGTTATTCTGAGTAAAACAATTGTTGTTTCCTATATGCATTCTGTATTGGTTACATTGTGACTGTGCACTAGTTTGATATGCTTGGGATAGTATCCCAATTTTCGAAGTAAGAATTTTGTTTCGTTTAGTATTATTACTTGCTGAGCATCCTATGTTCAGTGATTGGGTTTTCCTTGTGTGTTTCAGTACTGGCAGTTAACATGGTTTTTCCCCCTAGTTTGTCATTTTGGCTCTGGTGTCTACGTAATGTTTTAACTGATGTCTGATTCTGATTTCAAATATGTATTTACAGCCTTTGTGTTCTTTTCGTTGTATTTTTCCTCATGACAGTCCGGGGTGCACCCCCCTTTCTCTTG

*EF1-α(2)*

TAAGAACTAGGGTTTTAGCGGATATAAAACAAACTGCTTGGACTGTTAACGTTCTCCTCTTTAGCCTCCTCCTCCTCCGCCTCACTTCGACTTTCTACGTCTCCCCTTCTCTTTCATCAGCAGGAGCTTGATCGTCATCAAACTCCAACCCAATGGCGGTGATTTTCAAGGATCTTCACTCTGAATCTGGTCTCAAATCGCTCGAAGAGCACCTCTCCGGCAAATCATACGTCTCCGGAGATGCGTTGACCAAGGATGATATTAAGGTCTATGGTGCAGTTTTAGAGAAGCCTAGTGATGCTTACCCCAATGCCAGCAAGTGGTACGAGTGCGTCTCTAAGCAGCTTGCCTCAAGTTTCCCTGGCCAAGCTGTTGGAGTGAGGTTCTCCACTCAGGCAACTCCAGCTGTCTCTGTCCCTGCTGCTGAAGAGAAGAAGGAAGCACCTGCTGGAGATGATGATGATTTGGATCTTTTTGGTGAGGAGACAGAGGAAGAGAAAAAGGCTGCAGAGGAGAGAGAGGCAGCTAAGAAAGCATCCTCCAAGAAGAAAGAGAGTGGGAAATCTTCTGTCCTCCTTGAAGTGAAGCCATGGGATGATGAGACGGACATGAAGAAGCTCGAAGAGATTGTGCGGAGTGTCCAGATGGAAGGCCTCTTGTGGGGAGCATCAAAATTGGTTCCAGTTGGTTACGGCATCAAGAAGATGCAAATCATGATGACGATTGTTGATGACCTTGTCTCTGTGGACAATCTCATTGAAGACTTCCTAATGGTCGAGCCAGCCAGCGATTACATCCAGAGCTGTGATATCGCTGCCTTTAACAAGATTTAAGAAAGACCTCAAATTCGGATTTCCTCCCCCTTGTGTCTTAAAAGATTTTGTTTTCATTTGTGTGTCTTGTGCTAGATGTGTACTCTTTGAGCCTTTGTGATATGGGCGTCCTATAAAGGACAAGTGTTAGCACTGCGATATTATTTGAAGCAGGACAGATGTTTCTCTGGATGGCTTTTTGGTGATATTTTATTAGTTCTGTTCCTAAAGTCGTGATTCATGGTAAACTATTTGGCAGGGGGCCATTTAATTTACGTAGAGTTAACATGATAGTTTAGGTTGATCTGGATAATACTCGGTTTGTTCGAAGGGGTTTTGGCAAGTTGAGGAATGTGACTACTTTGATTGGGTTGATGGTGCTGTGTTTGACAGGGTCAGATCAGTGGTGGTGAGGTAGGTGAAAAATGAATCTTTGCTCTTTGGCGCCCGATAATGACAAGGTAGCATATGCAATGATACGAGGATGGGGTGAAAGAGTTGGCTGCGGTTGAAACTCCAGGAAGGCAAAATATTATGGAACAAGGTGTAGGGGTAGATCTAAGTGGATTCCTCTGCCTAATGCTTGATCATACTGGGTTGGGGAAAGGAAAGGCTGTATGATGTATTTTATAATCTGGACTCATGATGTAGTTGGTGAATGTGATGTAATGAAATCTTTTTATTTTTTTTATTTACAGGGTAATCAGGTCAGTCCATGGGACCCCTATCTTAGTGGTACTCAATGGGGCTCAAGGCCAAGGCGTGAGGGATATAATGTAATGGAAGTGATCCAATGGAAACGATCTAGGCGTCCCAAAATTGCAATCAATGTATGACATTGATTTTTCTTAAGAAAAAGAAACACGATTAGTGCAAAACAAAAAAA

*EF1-α(3)*

GGGGCAGATAACGGCTTGGGTGTTTACTCCATAATTTGTCTCCCTTAACTGCTAGATTTCCTAAGTATTTGAGAGCCCATTCTTCTTCTCGCTGAATTGAAGTTTCACACGGGAAGAGGGAAAGGCACGACATGAGTAGTGTAGCGCAAATTCCAACAAGCTTTGGCCATGAACTTAGGGCTTGCCTTCGTTGCAGGCTCGTCAAAACCTACGATCAGTTTAGGGAATCAGGATGCGAGAACTGCCCGTTCTTTGGTTTGGACAAAGATCATGAGCGTGTTGTGGAGTGCACTACTCCAAATTTTACTGGGTTAATATCTGTCATGGATCCAAGTAGGAGTTGGGCTGCTCGCTGGCTACGCATTGGAAGATTTGTTCCTGGCTGCTATACCCTTGCTGTGTCAGAGGCACTCCCAGAGGAATTCCAGGCCATTTGTGAGGATGAGCGCGTTCAATATGTGCCACCGAAACGGATGTGAAACCAGAGGTTTTTGTGGATGGAAGCTGATGATGATGCAAGGTGACTTTGCTCCCAAGTCATGACTGAGCAATGACTAGGGCTTACTGCCTCTGAATCCCATCTCTCTGAGGCTGTTGATGTTTTGTAAACGGTAAACCTAGTGGTGTGGAAATATTTA

*EF1-α(4)*

TTTTTTTTTAAGCAAACTAATTGGAGGCTTCATATATTTACTTGCAATATTAACTTCCATTCACCATCAACAGAGATCTTACACTGAAGAAACTGTCACAACCTACAGAAGCAACGTTAAGCTACCAAGGATTCTAGTTAGGGTAATGTGTTATCGAAAAGCTGACAAGAAAATTGCTCAGTACTAATGCATTGCAGAGACAGGCACACACTACCAGCCACCACCTCCACTGCTAGACCATCCTTTCGAGTTGCTCTTTGAACCAGAGCCCCAGCCGGAATCATTCCCAGCATCCGCACCACCATTGTTCTGGTCACCCCAGCCGCCGCCGCCGTTGCCGCTACTGCCGCCCCAACCACCGTCTCCACTACTGTTCCCACCACCAGGAAAGGCTTCCTTACCTGGCGAGTTTTGAACTTTGGCTCCAGGGAAGCTTCCCCAGCTACTATTACCTTCTTTGTTATCTGAGCTCCACTTTGAACCACGCCCAGAATCATGCCTGTCTGAGTTACCGCCATCTCTATTTCCATGGTATGAACCCCGACCACGTCCCCGCCCTCCATATGGCCTTGGTGCCCCACTAGGATGCCCATCACGGTCACGGTCACGATCGCGACCACCATTTCTGTAATCATTTCGGCCTGGCCTAGAACCTGGCGTAGAACGATCTCGGTCTGAAGACCCTCTCCACCCACCATCATTGCTGGCTGACCCACCCCATCCGCTGCCAGAGGAGCCACCAGGTGCTGGACTACGCATTGGTACCATGGCAGCAACTGATCTAATTGACGGTGACTCATGAATGGGATCATCAATATGCCTCTGGAAATACGCTACAAGCCTGTCAATATCCTCAAACATCCTTTTGCGGAACTTGAATCCTTTGGGATATAGACCTATATACTCATGATGCGGATTGGAGCTACGTATGTATGTGAGGATAAAAGTACCCGGGTGCTCATGAGAAATGCCAAAACTATAAACTATTCTCAGAGGGTATTCTGATTTCTCAATTCGAAGTTGTTCATCAACTTCTGCTTTTGTTCCCTTCCGGAATTTCCGATAGTTAAGCATTGCCTTGAGGTGAGTAACCAATGGATCAACATAACGATCCATCACCTCATCTAAATCTTCAAAAGTATCCTCCCCAATTTTCAGAGTTTTGCCAATACGAAGCAAGCTTGTAATATCTTTGTGGTCCTTTCCCCCTTCAATTATATCTTTGTGAGCATAAACACCATCATAGACCTTTAGAGTCAATGTCAGATAAGATGGGCCACGAGAACTTGGACGTATAATACTTTCACCGGGGTCTTTGTCACCCAGAAACTCCATTGCTTCATCAGCTGTTATGTTCTGGAAACGAGGATGAACAATCATCCTTGGCTTGAAATGCTTTTTAGCAAGCTCCTTTTCTCTGCGAGCCTTTTCTTGGTCACTCTGTAAGCTGCTTCGATCTTCATGATAGTAGGGATCCAGGTTCTCTACATTCTGATGCCTATTACTCCTCATGTCATTCTCTCTACAAGTGAGGAACACCTGGTATCTGTTCTTATGAATTTGTTTGATCCTACAAGTAAGAATATCACCTTCACGCAACTTCTCAGTCAAATCAGAATCCCTTAAATCATCTGAATAGTCTTCCCTTGTAAGCATGCCGGTTAATCCTGATTCCAGGGCACAAATTGCTCTTTGAGGTTGTACTCTGCGGACTGTCACTTGCACGGTCCGTCCTTCGCCAAGAGTATCATCTGTCTCCCCAGATATCATATAGAACTCTTCATCTTGATTCGGTTCTTCGTACGCTTTACGCCAGTCCTGGAAACCCTGAATCAACTCCTGTTTTATGGCATGCAAGGTCTCTATCTTAGAGCCACGGTCCGTGTCCTTCGCATATGCGTCAACATTGAAAGATTTTAATGCACTTGGTCTGTCTCTAAGGTTTTCAATTGCCATTTCAATTGCATCATCTTCATCTAAATTATCGTCCCCTACAATTTCCCTATATATATCTTTGGCCATTTCCTGAGCTAAAGCATAAGATTCTGGATGTATTCTTGTATCGTCCAAGACATCAACAAACTGGCTTGTAGCAGCCAAACCAGTACGCCTGATACGCAAGAAACCAGCAGCACTAATGAAAACCTTTCTCCCCAGTCCATGAGCAGTCAACAAGTCTTTTCGCGTGACAATTGCCCCAGCTCTGACCAATGACCTCTGCAAAGACGCTGCTTTCCTGGGCCCCAGGCCAGAAATAAACTGCAATGGGGCAAATAACCATTCATGATTGGCTGCCAAATTGAGATCAATGCCCACCTGGTTGCTCACATCAACCATGATCTGTTCCACCATGCCATATTTCTCATCAGGATTGAGAAAGCTTTCTAAAGGACTAAGTTTCCAAGACAAAACTTCCCTTCCAGGCCCACAGAGTGTAGCAACCATTGCTAAGGGATTTTGAAGGCACCGTCCAAGGGCAACAGCCCGTTTCACAATGCCCGGCTGCGCAGGGATCTGGTCAGAAGAAATACGTGAATTTTCATATAAGCGAGACAGAGATTCATCCCCATAGAAAACACTCAGTCCATCCATCTCATGGCCCACATCTCTAGGATTTTCTTCAACCATCTTAAAGACAATCTCATAAATATCATCCTTCAGCCGTGTGCAAGACAAATTCACAGCTCCCAATACCACAACCTGTGGTTGGTGTTCTGTCATAAACTTCAAAAGATTCTGCTGGTCGTGTTTTTTACGCTGCTGGTCATTAACATTTTGAGATCGTAGGCTGATAGACCCAGCCTCCAGCACATCAACAATCTCTCCAAATGAATCCAACATTACAAAGGTAGTTGATGGCTTTCCAGGACCCCAACAGCAAGCCATGACCCTTGGAGCCGCCTCATCTTCTGAGCTAACATCACTTTCCTTGCGCTGATATGGAGCAACAGACACTTTGTCCCATAGACGTTTCCCATAATCCGTAAGCAACCAACTCTTGGCCCTACTAGTCAACAACAATCTAGCTTCCTTCTCTAAGGCAGGCAATATAAAATTGAAAATAGCATCCTGTAGTATTAATTTTCTCTGCTCATTCCAAAGCTGTGCCGACTTGCTCACACCACAGCTAAGGTATTTCTCCTGAGAATCATTTATGAGCTTCTTGAGAACTTCATCCGGCAATTTAATGGTAACTTGAAGGAGCTTCTCCTCTTCTGCCTTTTGGATAAGAAGCCACTGTGCATCCTCAAATTTGTTCAATGGTTTGTTACGTAACCACTTGACACCAGCAAACTGATGGAAAGCATCTATGACAGCATTCCCATCAGGTGTAGGGCATGTTGACACCACTGCATTGTCAAGATATATACTACGAAAATGCTTTTTTACCACAGGCTCACAACTTATCTCGACAGCCGCCATGTGCCTTGCTCCTTTAAGCACAGCTTGAGCAGAATCAAACATCGCACAAGTGAAATTTGAAGCCACATCCTCAGGTGTCTCCTTCGGATCCTCTGGTTCATCCACTCTCATTTCTAATAAAGAAATCTGCAACCCAAACTGCTCGGAGCTATAGCCAAACTTGCTTGCAACCTCCCATAAACCAGCTTTATTGCATATGCTGTAGAGTGATTTCCGCTTGGGCCTTTTGTATTGCCCCTCATCGACACCAGCCTCACCAGGAGGGAAATGCAAATTAAATTTTGCATCAACATCATCAACTTCTCTTTCTGATTCCGCATTCCTTAGTGCACCAATAATTGATTCGAAAAGCTGCCGATTCAAACTGAGTCTTGTCTCATCATATATTCTGCGAGATTCTTCCTCGAATCTTTTATTGTAGTAAGCCAGAAGAGCACTCTTTCTCTTTTGAAGTAGCAACCACTTCTTGTCCAAGTCCTGGACAGCCCAGAGGACCTTGTGCCATTTTAAAGAAGGTTTATCATGATTTACATCGTCACCACCTTCTGGATCCTTCAACAAGTTAAAAATTTCCTCTTTGCGGTACATAGAAATATAAGGGACATCAAGTTTCTGAACATGTGTAAAATTCAAAAAATTTTTAATATGATCCTCCATTTCTTCTCTCTCCTGCTCGCTGAGTGTCATCTTCACAAGCACAGGGACTAAACCAGATTCTAGTTGCTTTCTTATCCACTTGGACTCTTCATCTATTGCCTCAGCATCAGTAGGAGGAGGACCAGTGCTCTCCTCAGATATCTGCATCCTCTCAGGTATATCAATCTCCCGAATACGATCATCTTTCTCCGTCATATACCTCTCCGCAAGGATGATAGGCTCAAATTCATCTTCAAGCCTTCTTTCCCTCCAGTCACCAGAATCATCATATCTGCTACTTTTATCAAGGCTCATCTGTCGCATCTTTAGCAATTCTTCAACATCACCAAAAATCTCTTGGGCTTCCTGCAAAGCAGACGAAGAAACACCGGGAGCTTGCCTATACTTCTTCTTCTTTACCTTTTTTCGCCTAACTGGAGCCCCCGTCTCATCTACTTCTTCTTCATCCACGATAAAATCAGCCATATCATCCTCATCGCCCATGTCTGCATCCTCCTCTTCCGGCTGGTCGTCTTCCTCTGGGATATCCTCAACTGGTCCTCCATCATCATCGCCAAATAAAGTGCGTTTAACATGCTCCTCAGCTGTTCTCCCACCTTTCCCAGTTCCATCATAATCTTCATCATCATAAAAACCAGAGTGTCCCTCTTCAGCATCACCCCGGGCTTTCTTCAACCTCTTAAACTTTTTGCTTTCATTCATGGATTTACGAGGAACAGCAACATTATTGTCCAGCAGAAGTTCATAGTCATCTTCATCAAGTACAAAAGACTCCCTCTTCTTTTTCTTCTTCTTTTTATGTCTCTCCTCCTCGTCGTCCCTATCTTCCTCTTCAGGTTCTTCATCAGCATCCTCCACAATGAAACCATCGTCCTCAAACTGATCCTGTCCTTCATCTTCTTCATCTTCATCGTCATCACCACCGGCGACATCTCCTTCTTCGAGGTCTTCACCATCTTCATCTTGCTCTCTCTCATCATCCTGAGGCTCCAGCTCCTCTTCGTCATCTGAAACCACAGCTCTACCACCCATTTTTGGCGACGAAAAGCTAGGGCGAAAACCCTAGAAGCTAAGACAATTGAGGAATCGAGAAGAAATTGCAGAGAGAACTCTCTCTCAAGCTTCGGCGAGTTCTTCCCCAAATTCACAGCACGTCGAAGCAGGCAGTGTGAGAGCTTGAGAGAGAGCAAATGAGAGAGAATTGGTTTTGTCAGGAGATGGCAT

*EF1-α(5)*

CCAACAGGGGGAAAGGGAAAACTAAAGTCCGGCCACGGAGCGGGACAATGTCATTTTGGTAAACTCCGGGGGATGTCAATTTCGTCAACTCAGCATAGGGGCGCACCGCTCTCGTCCCACTCTCTCCCAGTGTCCAGATTATACAACGCTAATGTGAGGTTCCAAGCAAAACCTCTTTGCTGCTTCATTCAGTCATTCCTCATACCCTTCCTCTCCTCCCCACTGCGGCTAGGGTTTTAGCGGAGCTCTCGAAGCTTCACATACTTCATCATGGGGAAGGAGAAGGTTCATATTAACATTGTGGTCATTGGCCACGTCGACTCTGGAAAGTCCACCACCACTGGTCACCTGATCTACAAGCTTGGTGGTATTGACAAGCGTGTCATTGAGAGGTTCGAGAAGGAGGCTGCTGAGATGAACAAGAGGTCATTCAAGTACGCCTGGGTGCTCGACAAGCTTAAGGCTGAGCGTGAGCGTGGTATTACCATCGATATTGCCTTGTGGAAGTTCGAGACCACCAAGTACTACTGCACTGTCATTGATGCTCCTGGACATCGTGACTTTATCAAGAACATGATTACTGGTACCTCCCAGGCTGATTGTGCTGTTCTCATCATTGATTCTACCACTGGAGGTTTTGAGGCTGGCATCTCCAAGGATGGTCAGACTCGTGAGCATGCTCTTCTTGCTTTCACCCTTGGTGTGAAGCAAATGATTTGCTGCTGTAATAAGATGGATGCCACCACTCCCAAGTACTCAAAGGCCAGGTATGATGAAATTGTGAAGGAAGTCTCATCATACCTTAAGAAGGTTGGATACAACCCCGACAAGATCCCATTCGTCCCGATCTCTGGGTTTGAGGGTGACAACATGATTGAGAGGTCTACCAACCTGGACTGGTACAAGGGTCCAACCCTGCTTGATGCCCTTGACCAGGTCCAAGAGCCCAAGAGGCCCTCAGACAAGCCTCTTCGTCTCCCACTCCAGGATGTCTACAAGATTGGAGGTATTGGAACTGTCCCCGTTGGTCGTGTTGAAACTGGTATCTTGAAGCCAGGAACGGTTGTCACCTTTGGTCCTACTGGGCTGACAACTGAAGTTAAGTCTGTTGAGATGCACCATGAAGCTCTTCCAGAGGCTCTTCCTGGTGACAATGTTGGGTTCAATGTGAAGAACGTTGCTGTCAAGGATCTCAAGCGTGGTTTCGTTGCCTCTGACTCCAAGAATGACCCAGCCAAGGAGGCAGCCAGCTTCACCTCCCAGGTCATCATCATGAACCACCCTGGACAAATTGGAAACGGCTATGCCCCAGTGCTTGACTGCCACACCTCCCACATTGCCGTCAAGTTTGCTGAGCTTGTGACCAAGATTGACAGACGATCTGGTAAGGAGCTCGAGAAGGAGCCCAAGTTCTTGAAGAACGGTGATGCAGGTATGGTGAAGATGATTCCCACCAAGCCCATGGTGGTGGAGACCTTCTCTGAGTACCCACCTCTCGGTCGTTTTGCTGTGAGGGACATGAGGCAGACTGTGGCTGTGGGTGTGATCAAGAGTGTTGAGAAGAAGGATCCTACTGGTGCCAAAGTCACCAAGGCTGCCCAGAAGAAGGGAGGAAAGTGAGTACAATTGCAGTTTTTATCTTCACCTGCTGGAGGACTTAATGATTTCTTTTTTATGAACAGTAAAACAATGATCTTTTTAGCACTTTGCGGTTGGCCCTCTGGTTTGTTTGCTGTTTTTATGGTGGTCGAGAGTGTCCTCACTGATATCGGATCCCAGTATCAAAATTTGGTAGGGATGTGCTGCCCCTCTCCCTTAAAGTGTCGATGTTGCTATGATTTTGGGTTGTGACTGTGCAGCATGGGAATTTTTACCTGTTCCTTTGCTGTTGCCAGGGAGTCTTATAGTTAATTACAATGAAGTTTTGATTTTAGAGTCAAACCCCGATCTTCCTCCTGAATTCTCTCCAGCAATTTCTGTACTTTTGGCTTGAAGTTTCTCTTTATTAAGAGAAAGGAAAGAAATGTGCAAGCTTTCCCGGCTCAAGAGTCAGGATGATCCTGGCGCGTTTTGTTGTGGTGAATGTATCATTGCATTGGTCATTGTAGCTGTTCTTCAGTGGTAGAGTTGAGAAGGAAGATTGGAAAGTAGATGCTAATACTAAAAAGAAAAGATAAAAGAATTTGCCAAGCAGGGCTTCCCAACTCCTAAGCAAATTACTCAAGAAAGCCTAGAGTTAGATAGACAGTTGGGTGTATGTGTTGAGATTGTGTCGAGATTTTAGTTATTAGGAGAATTGTAAGGAGTTTTGAGGGAGAAAATGGAAAAGCAACTATTATGCTCTTTTGAAGGGTTTGTTGAGTTGACAAATGGGTTTAGTTGATTACGGTGGCATATCAGTAATCAATGTTTAGTGCT

*eIF(1)*

TTTTGATCAGACTTTAACTCTTACGGCGCAACTGCAAAGCAATAGAGTAATATCATCAATTACTGTCCTGACATTCACATGCCATTGATCTTTGACGAGTGCCGCAAACGCTAAGAATGCGGGCATAAAATCCATTGAAAACGCAAATTTGTTCTCTATACAGAAACAAAACATAACAAGGGTTGTTTTCCACGCAAAGCCAAAATTGCAAGGCTACAGACACTATAAGTTCAAACTGCACTCGTACGTGTGCAGAAGCTTGCCAGGGTGATCAACTGGACCTTAAACTCATATATAGCTGTCAATCAAAGACTTTCTCATCTCATCAACAATGGCACTAGGTTGGGCTTCCCTCAACTTGAACACGCTCTCAATCACTGATAACTCAGTGGCAGTTGTGTCTGACCCACAGAAGGCTGTCCAGTCATTCACCGTCAATCCAGCAGCAATTACCTCACTTCCCCTGTTCACAGTCCCGGCCACAAGGGGAACCTGAAGAAGGGTTGACAGCTCATCCAGATCTTCTATGGATGTATGGGGGTGGACCAGGCCACCTTTGTTGGAGAAAGCACAGTAACTCCCAACCAGAATATTACCAGCAATCGTCTGCCTGAAAACCTCAACACCAAGAACGTCTGCAATTATCTCCTCAGTTTCCTTATCAAGATCAGTGTGTGTGAGAGCAACATAATCATTGCATGCTATGCAGTTTCCTAGAGCAGATAACTTTTCTTCAATACGCTGAACTACTACTGAATCAGGAAGACTGTTCCTCAAGTGCTGAAGTTCCTGGTCAGTGGTGGTGTGAGGCACAAGTAGCCCATTTTTGTTTCCAGCACACATCCGGCCAATGATTCTCGTCCCTGCAATGGAAGTCTTCACAACAGGAATATGCTCTGCCAACTCAGACTCGAAAGTGCTGTAAAAGTTCTCTGAACCACCAATTGCGACTAAGCAATACGCGTTGGTCAACTTGGAGAAGACTCCAACTTCACAATTGTTCTCAAATTGAAGCCTCGTTGCCATGATGAGATGAGCTTGAACAAATCGATGAACCAGAAGATTAACCGATGGCGGCTAGCTAGAGCGAGCAAGACAGACGCGCACACACAGAGACTGTGAGAGAGCCAGAGAGGGACGCGGTGAGAAATGGCCCAACAACTTTTACTCAAGAGCCAGAGCAGTGATGCATAGGGGGGATTTATGTCACACCCCGAAACACCCCCAAAACGAGGAAGGGTGTGCAGGCGCAAGGCACGAGGGGTATATAGCAGTATAATGAGCACTTCCAATGGTTGATTCAGGGGACCAAAATTTGAGGGTTGCAGACTTTTAGGCCACTTCTGCTTCCTGCAGTAGGGGAGCCGCTCGGCAGCTCTTGAGTCAGGCTCAGAGCAGTGGCAGCGGTGGGCACACGAATCATGGGTGGAATCCGCGTGTGACACACGTGTCCAGCATCTCCG

*eIF(2)*

GCCGGTCTTTGCGGTTCCAAGGGCTCAATGGACCCTCCATTTCAGTCCAGTATTCGTGACAGCTTACACAGCCAACCTTCTCTATGCTATCTTTGGCAGATACAATTCAATATAATCCATGGACGGAAATTGATATACATTCTACAACCGCTAACATCAAAGTGAGCAGCTATGCTAACAAATAGTGTATCAAAAAGTGACTTATTCTAAAACATGGCTTCCATTGATTTGGAAAATGAGGGGGAAAAATTCAGGTGTACCAAAGGGTGACTATCAAATTCTCAGATTCCATCAAATAGTATCTTTCAAAACAAAGAACGAAATCATTCAACGTCCACACATACAAAAATTTCACTGCCTGACTAGTGTAACTTTGTGACCAACTCGAAGAAGTTTAACTGGGCAAATATGATAATTTTCTTCTAATGCATTTCTTTCTCCACGCCAGCCAGCCACAAATAATATCATACCTCAATTCATTTGGCGGCAGATAAAAAGGATCGTTGGTTTCAGTATCCGGAATTCAATAAAGATGCCCAAGAGGCACAAGGATCCTGATTTTCTCACATGAAATCATACTCATCCACATCGTCATAGCCGGAGGCAATAACTAGATCATCATCAGGTTTATCGACATGAAGCTGCTTCTTCTTTGCACCTGCTTTCTTTTTACCAGCATTGGCCTCCTTCTCTGCCTTTAGCTTTTCATTTGCAATTGCTGTAACTGAAGAGGCAACCTCCTTTGCATCTGCAGCTTTTAAAGAAGTCATTGAAAGCCTCATCACTGCCTTCAGTAACCCAATATAATGGTAGCTTTTCTCATATGGACGAAGCTTATGAGAAACAAGTTCAGCATATTCCAAGAAGTCACTTTCAGATTTAGGTATAAAATTATCAAGGGTCTTTTCATCACCCTTCTTAGCAAACAGCTCAGTGGTGGACCTATAATCTGCCTCCTCCACAAGCCTTTGCTGTCTAAGTTTCTCTGCCACAGGATCCAATGTTGTTTCTTGTGCTACTTCCACATTTTTCCCTTTCTTCTCAGTGTCTTTTGTTCCGCCTTTCTTCGAAGGCTTTTCGGGGGCTTTTACTGGTGTAGGTGCCGGTGCAGGATCATCATCGTCCCAGTTTTCCTTCACATCATCCTCAGCATCTTCGTCATCCCAATTATTTTTTGGTAGTTCCTTTTTGGCAAGTAGGGGCACAGGAATATCCTCCCAGTCATCCATGTATGAGCGCTCCTTCAAAAAAACTAATCACAACTCAATGCTCACCGCTACCACAAATCAACGTACTGCCTGAGTCGCAGATCTGGACTGGGAACGGGTCGGCCGTCGGCGGCGACGCGTTGGAGTCTGGCGTCGGAGGTCAAGTAGCAGTGCGGCGGTGGGAGTCAATGGAGAAGGAGGAGCGCGAAGGGCTAGGGCACGGCTCACTTTCTGCTTTGAGGTGCTTTTTTTTTGAAAAAAAAAAAAGTTTAGGAGTAATGCCTCTGGCCTCTGGG

*eIF(3)*

GCAGAGGCCGTCTCCATCAGAATGAAGATTTAGGAATGTGGATTTAGAAACATAGACCACGGCTCCACAGTAGAAACAGAGGTACAACAATCTAATTGAAATTCGGTGAGATTTTGTGAATCTCACGGTCAATGGAGGACATTAGAGTATGACATTAAGGCCCTAGCTTTGTTTCGCTGGAGGGGAAAGAAGAAATGAGAAACGCCTCTGTCTTACTCGTCCTCTTCGCTGTCATATTGGCACAGAGACTGAAGTACATTGCTTCCGCTATGATTCCCTTGTTGATCACCCTCGCCATCCGCATTCAATGCAAGTTCTTTCTCCTCTGGTCTTCCAATGGCTTTTTGAACAGGTTTCTTTTTAGCCTTGACTTGAATTGTTGGCAACTGCAAAATTCTCTTTCCATCAGCAGGCTCTTTGATGTTTGTGTGCTCAGAGACGCTGCTCTTCATCAAAATGTCTGTCGGCTTACATGGATCTTCTGAAACCTTGCATCTCTTCGCATTGTGATTAGATGCTTCATTTCTATTGGACATCTCATCATCATCGTCACCATCACCCCACTCTTCATCATTAATCCTTCGTACAGTATCTTTGGCCCCATAAAATATTGATTTAATAAGAGCTTCATCTTCCTCCTCTAGCTTCCTTTCCTTTTCCTCACCCGTGCGCTGGATAGCCTCAAGCATATCTTCTAGACGGACTGTAGCTTGCCTTGACTTCATGGATTTCATCTCATCAAGAGCAGCAAGGATATCCATTTCTTGTTTGGAGTCCAAAGTTCTATTCTCCAAAGATTTCATTGCATCTCCCAATTCTTCTGCATCTCGTTTCCTCTTTTCTTTCTCTAACTCCTCATCTTGATCCCGCCAAGGTTCAAAATTCCGTGCCGCACCCGCTTCAACAACATAATCTGAGTTCTGAGGATCTGTCTTTATGGCAAGCTCAGCAGAACATTTGGTGCATTTGAAGTAGAATCTGAATATTTGAATACCCAAGTACGTCTCTCCGATGACATCTTCTTTGCGAGAATTGAATTTGGTGCCCTTGTAGATGTAATTCCCACATGTATTGCACCGAATACTCATCGGAAGCATCATTCTGACCTTAATCTGTTGATTCTTAGGCTGTCTACGCCGGGGGATCTTCGCCGGATCAAAATCCGGAGGATAATACTTGTTCAAAACTTTCCGCTCTCCCATTGTTCTTCTCTCTCCTCAGTTCCCCGAACCCTAGCCTCCGATGTAAAAATTCAACTTGATCTCTCTCTCTCTCGCTCTCTCTCGCAGAATCTCAACAATGGCGACCTTCGAAGTCGGCGCCGTTCCCTTCAACCCAGACGGCTGGGGTCCGCCGGAAACCTCCGCAACCACCACGGCCGTCAACGTCCCCTTCGCCCCCTTCTCACGCTCCGACAAGCTCGGCCGCATCGCCGACTGGACTCGCAACCTAAACAACCCAGCGAACCGATCAGGGCGGAACAACCCGTCGGAGTCCGTCTTCGACTTCACCGGCGACGACTCCTTCGCCGCCACACTTGCCGCCGACGAAGACTCTTCTTTCCGCTTGGTTGACGGCAAGCCTCCTCCGCGCCCTAAGTTCGGTCCGAAGTGGCGCTTCCACCACAACCAAAATCGACAGTTCCCTCAGCGCCGCGACGAGGAGGTTGAGGCTCGGAAGAGAGAGGCTGAGAAGGAGCGAGCTCGCCGAGATCGGCTTTACCACCTCAACCGCGGTGGCAACACTCAGCGCCGTGAAGCCGCCATATTCAAGTCCTCCGTTGAAATTCAGCCAGAGTGGAATATGCTTGATCAGATTCCCTTCTCGACCTTCTCTAAGCTATCGTTCTCGGTTCCAGACCCGGAGGATCTCCTCTTCTGCGGCACCCTTGAGCCCTATGATCGCTCTTACGATCGTGTTACTCCGAAAAACGAGCGCCGCCTTGAGCGATTCAAGAACCGGAACTTCTTCAAGGTCACAACGACTGATGATCCTGTCATTCGTCGCCTTGCTAATGAGGATAAAGCCACTGTGTTTGCTACTGATGCTATTCTTTCTACGCTTATGTGTGCTCCAAGGTCTGTCTACAGCTGGGATATTGTTGTTCAGAGGGTTGGAAACAAGTTGTTCTTTGACAAGCGAGATGGGTCTCAGCTTGATTTGCTTTCGGTTCATGAAACATCGCAGGAGCCGCTGCCTGAGAGTAAGGATGATATTAACTCGGCCCATTCTTTGAGCGTTGAGGCTGCTTATATCAACCAGAATTTCTCTCAACAAGTCCTAGTGAGGGATGGGAAGAAGGTAACTTTTGATGAACCGAACCCTTTTGCGAATGAAGGAGAGGAGGTTGCTTCTGTTGGGTATAGGTATAGGAGGTGGAAGCTTGATGATGATATGTATTTGGTGGCTAGGTGTGAGCTTCAGAGTGTTCTTGAGATTAATAACCAAAGGCAGTTCCTGACCCTGAATGCTTTGAATGAGTTCGACCCTAAGTATTCTGGGGTGGACTGGAGGCAGAAGTTGGAGACCCAAAGAGGGGCTGTTTTGGCTACCGAGTTGAAGAACAATGCTAACAAGCTGGCTAAGTGGACTGCCCAGGCGCTTCTGGGTAGTGCTGATATGATGAAGTTAGGTTATGTTTCAAGGGTTCACCCAAGAGATCATTTCAACCATGTCATTCTCGGCGTTGTTGGGTATAAGCCAAGGGACTTTGCTGCCCAGATTAATCTGAACATGGCTAATATGTGGGGTATTGTTAAGAGCATTGTTGATTTGTGTATGAAGTTGAATGAGGGCAAGTATGTGCTGGTTAAGGATCCATCGAAACCGCAGGTTAGGATATACGAGGTCCCACCTGATGCATTTGAGAACGACTATGTCGAGGAACCATTGCCGGAGGAGGAGCAAGCTCAGCCTCCAGCTGAGGACGCCAATGTGATTTCTGCGGAGATTAGTGTGGCAGCTACTGATGTGGAGGACAAGGAGATTGATGCTGTGCCTTAAGAAGGATACGCTCTCAGCAGTACCATGCGGTACAAACAGCAATGGGGAAAGCATGCTCTGCATATTTTGCGCTGATAACTAGGGCTCAAGCAGAGGGCCATCCTCTCTACAGGTCTGAATTTTTGTAGATGAGCCGCGATTCGTGAAGTTTCCTTTGCTCTGAGTGTGGTGGACATCCTAATATAGAGTTGAGGTGAAGGATTTTGTTTTCTATGACGACCTTGACTGTTTTGTTTATTTTGTGTTTCCCATACAATTTCATTAAGAAGTTCCAAGTGATCTATCTTGTTGATGTTGAAGGATGTTTATGGCGTATCTTACGGTGCAATTTGCCGGCAACTTTTTGTCAATTATATGTACTTACCATTGGTACTCAATATTCACTAGTACCTATTGGCGCTCGTCACTCGTCCACATTGTTCGTTAGCGTTTTAGTCATGTTTTCTAGTTATAATTTCCCAATATTGAGCACTTATTGGCACTTATTAGCATCCAACAGAATTGGTTAGTGTTCGTCAG

*eIF(4)*

AAAAAAAAAAAAACTAAATCAGAGGGCAGGAGACAACCGTATTTCTCTGTGAACCAACCCTCCAAAAAGAAAAAAGAAAAGAAAAAGGGAAACAGATGAACAGTTAAATAACCTAATCAGGCAGGGCCAGCTCATCATCCAGACAGACAGCAAAATCAATCTTCAAAGCGACTCCATTAATCCACTGTATCCAGAATCTCTCTTAGACAGCAAGCCATCACAAACGATGCATGCAAGTCACATTGGCAAGCAACCAATTTCCCCTATTCATGATTCCGCGAATGAGAACGGATCATTTTCAAGCAGCAAACAAGCACGGGGAACCAAGCATCAGTAGAAGGACGCAGATCACGAGTCTCAGCAAAATGGATCATCGACAACCAAGACAATTACCCTGAACTGCCGCCATTCACATGCAAAAGTTAACTTCAGTCTTAGAGTCGAGACCTAAGCTCCATAGAGGATGATGACTCGTTTGCCTGCACAGAATCCAGTGTGACACAGGAGCCACTATATAGCTGTGGGTGGAATTTTGCATGATATTCAAGTTTACACCTCACTGAATCTTGAAAAGATTCTTCAATTTTACGACCAACCTCCACTCCTCATTAGTTAAATCATCCCGGTAATTAGACTTCAGCACATCAATTGATTTCCTAGATATGCGGCTGACAAGCTCATCATCGATGTCAAAATGCCTTCCATACATTTTCTGCTGCTCCTCAGGATTACCACCGACAATCTTAATGGCCACCTGCTGCCCTTTCTTTGCAATATCAACTGCTTTGTGATTATTCTCAATAGATGCAATCCTACCAATGTCTATAAAATCCTGTGAAGGAATGCAAATTGGAGTTCCCACCTTAGCGATTCCATCAAGAACATCCACTCCCAAGACAATAGGATCTTTTTTGTTGAACACGCAGTTAGGAATTATCTTAAGTACACAAGGAAAGACCGCTTCATCGGCAGCTTCCCTTTTCTTTTCCTCCTTGATATTCTCAATATAAGCCTTGAATTGATCAAAGAGATGATAAATGATATCAGCCATGAAGATCTTAACACCAACTTCCTCTGCATATTCCCGTGCCTCAGGTGTCACCTTGACATCAAAAGCCAAGATTGTAGCATACTCTTTCTTCCTTTCAAGCATGACACTAGCTTTCATGACATCTTTCTTGTGTACAGGACCTATACTTATGCTGCTGACAGGAATTTGAACAGCTGGAGATTTCAGAAATTCCAACAATGCTTCCAAGGATCCCAGAGTAGATGCCTGAACACAAACACCCTCCCCACTCTTGTCGATTCTGCTCAAGACAGACTCCATGTCTTCCATGGCAAATTTCTTTATATCCTCCAAATCATCATCAGGCCCCACAACATACAAGCCAGTTCCCGCAATTGCATGCTCAAGGCCCTGTGCTGTTATCTTGATGCCCTGTGCTGCCTTGATTTCCTTGTGATGCAAATATGTTCCCTTAACTCTAAGTTCCTTCATTGGATGGGGAGTCAACAGAGCTCGAATTGTCGTAACAATGGGCCCCTGCATGCCACACACAACAATCTGGTCCCCTTCATGAAGAATGCCATTTACCAACACAACATCTATAGTTGTTCCATGACCCTCAACGACCTTCACCTCCAGAACTGTACACTCGATTTCATCGCTGAATGTAAGTTTCTCAACCATTGTTTTCTGTGCCCACTGAACTAGCAGTAGCAGTAAATCAGGGACACCTTCCCCAGTTATTGCACTAGTTGGCACTATGCTGTATGTTTCCCCCATTTCTTTGTTTTTATAGTACAGTTCAGTATTTAATCCTTGCTCCTTGAACTGTGTAATTATCTGTATGAGCCTCATGTTGAATTCATTCTGCACATCTTTGGTTTGTCGTTTCAGGGCCTTAACAATGGGCTCATTGCGGTGAGATTTCCACCCATAAAGTCTATCCACCTTGTTCAATGCCACAATGAACTCTGTATTCCTCATTCTCAAAAGGTTGAGCGACTCAATTGTCTGTGGTTCCAGGCCATGCATGATATCCACAACCAAAATCGCAATATCACAGAGTCCTGAACCCCGAGATCGCAAGTTGGTGAAAGACTCATGACCTGGAGTATCAATAACGAGAAGACCAGGGACGTTCAGCTTTGCATCAGCCTTCAATTCCCTAGTCCTCTCCCTAATATTTTCAGCGGGAAAATAGGTTGCCCCAATTTGTTGTGTAATACCTCCAGCTTCACCTTCCTGAACATTTGTACCTCTAATGCAATCTAGCAGCTTGGTCTTACCAGTATCAACATGACCCATAATACAGCAGATTGGAGACCTGAGATTTGCTTCAGCCTTTTTGTCCTGGGAACCTGAACCCTTTCTAACTTCAACGTCTTTACCCCTGTTCTTTGCGACTGGTTCAACCTCGGTATGCGTTTGTTTACTCTCATCAGTCTTGGATTTAGTTGGCTGAGATGCCATTTCCTTCTTTGAAGGTGCAGCCGATTTAACAACTGGACGTGATAGTGCAGTATCATGAACAGGTTGGGCAGTCTTTTTGGCTTCCTTTTTAGCCACTGACAGAGCCCCAGAATCAACCTCTTCATCCTCAAATGCTCTTTTATCAGGGAGAGCAAGGTCGGCATCATCCCAACTTTTAGCATCCCATTCCTCATCATCATCTTCCTCTTCTTCAGCAGCACCATTTTCTTTTATGTCCTCAGCAGCTTCTCCCTTCTCCTCTAAACCAATAGTTATATCAACCTCTTCCTCCTTTTCTGCCTCAACATGATCAGCAACTGAAGCAGTGTCCTGTTCAGTTTCTTGTTCCTCAGCACTTTGGGTCCTTCCACCATTTGTTTGTCCGTGCAGTGGCTTTGACTTCTTAGTGGTGTAAATAGGACGTTTTGCTGGAGCAGCATTGTCCCCAGCAGGAAGAGAAAACCCCTTGCTAGCAAGAAACTGGTTCCTCATTGCTTCTCTCCGACGTTGTTCTTCTTTCTGTTTTGCAGTCAGCAGCTTACCTTCCTGCTTTTTCTTCAGAAGCTTCTCCTTTTCCTTCTCCTTCTTTCTACGCCTGGCTTCTTCAGCTTGCCTCTCAAGTTCCTCTTGTCTACGGCGCTCTTCTTCTTCCTTTCTCAGCCTCTCCTCTTCTTCTCTCTTTTTTCTTTCTTCAGCTTCTTTACGCCTAGCAAGTGCCTCTTGCATTTCCCTAATATGCTTGGGGATTTTCTTGTCAGCCAATTTGCTCTTTGGATCGTTCTTGACTTCTTCCTTCATCTCCTCCTTCACTTCTGTGGTGGTTTCAGCTGCGGCTGCTGCTGCCGCCTTTTTCTCTTTCTCTTTTTCCTTCTTCTTTTTCTTCTTCTTTGCCGCTGCCGACTCTGCTACTCCTTCTTCACCCTCTTTATCACCTGCTGTTTCAGCTAAGCTGGAGGGTTCAGGCTGTGCAATTTTCTCATCTTGAGGTGCTGGAGCTGGTTCGGGCTTTGAAATTGAAAGCCCTTCCCCAAGCTCTGCCAGAATCTTATCCAAGTCTTCCTCTTCTTGCGCTGTCCTTCCACTCTTTTTCTTCTTTTTTTTGCTCTTTGAAGTTTCAGCAGCTGCTTCACTGGGTTTTTGTTTTGCATTGTCCACTTCAGCACTAGTGGGGGCAACATCACTAATTTTAGGCTCATTCTCAAGAGCTTCATCTTCTACTGCAGTTGAAGCAGCAGAACCCTTCTTCTTGGAAGATTTCTTTCTACCAGTAAATGCCATTAGCCCATCATTTTCGTCAGCAACACCAGCCCCATTGGCCTCTTCATTTTCTTCATCCAACAATGCATCACTGAACACACTGCCAATCTTCTTTGCAGACTTTGAGGACTTTTTCTTCTTTCCTGCAAAAGAAATCAGCTCAAAATCCTCACTCTCCTCCTTGTCATGAGTAACACCGTCATCACCTCCCAAATCATCAATTTTTGGTTCTGCAAACACACGTCCTGGAGGTTTTTTACTTGATTTTGACTTCTTTTTCTTACCGGAAAAGGAAACAGAAGCAGCTTCCTCATCATCATCTGATTTGCGCCCATCCTCTGTTAACTCCGTAACAGTGCTACCATGCTCCTCGCCCTCATCATCACCAATAGCATCAGAAGCTGAGGCAGAAAACAAACGGCCACCCTTCTTAGAGCCCTTTGATGATTTCTTCTTCTCTGTGAAGCTTGGAGCTGGTTCATCCTCCGCACTTTGAACAGTCCTCTCTTCATCATCCTCCCCATCATCCAATGCAGCAAAGCTAGCCTTTGAACCACCCTTCTTAGAGCCTTTTGATGACTTCTTCTTCCCTCCAAAGCTAATGACAGGCTCATCATCCTCATCACTCTGACCATTTTCATCATCTAAACGATTGAAGCCTGCCTTAGCACCATCCTTTTTCAAACCCTTTGATGGCTTTTTTTTTCCAGCAAAGCTCACTACCGGCTCATCATCCTCAGTCTGATCATCCTCCTCTTTATCATTGTCCTCGTCTTCCAACACATCAAGACTCACCTTAGTAAACAAGCTGCCCCCACCCTTTTTCCCCTTGGACTTCTTTTTTCCAGCAACTGACGTCTCTGGAACGTCCTCCTCATCCTCTAACTCCCCAAGATTCTTAAAATCATCATCCTCATCCTTCAACTGTGCCCCTTTGGAATTTCCCTTCTTACCCTTCTTCTTCCCACCAGCTGGAGCCACCTTATCTTCCTTAGCCTGGGATTCTTCAGACATTTCAGTCCCAATTGAATACTCATCATCATCTATGACAAGATTCTTCTTCTTCGACCTGCCACCCCCACCCCCCTGTGGAGGGGCATCGTCATCCCTTGATGTAGGCTTCTTTTTACCCATCTATAATCTCCTCCTTACGCCGGCAAATACTTATATTGCTCTGAAGATCAGAACTCCGATGTTGTGTGATACGCAAACTCAGAAACCAGGGGAACGGAAAAACGATAGATAGGAGAGACGATAATAATTAGGGTTTTGAGATTATCAATCGGAAATCTTCCTCTCTCTTTTTTCCCTTTATTTTACACAGAGCAAGTGAAGGTTCAGGGAGAGGGGGTGGACGCTAGAGTCGAGAGAGAAAGAGAGAGACGAAGAGAGAGAGAGAGGGAATTGGGAATTATAAGAAGAGAGAAGACGGAGGAGGTTGAGGAGAACCCAC

*eIF(5)*

GTTAATTAGGAGCGAAATCAGGTTAGTCGCGCGACATAGTAAGGGCCAATTGAGGTGAACTGGAAATTCAGGCCTTAAACTCGTATAAACTGGCGTCGTCTCTATCTCCCGCTCTCTCTCTCTCTCTTCTACAAATTGAAGCGTCGAGTCGTGCGAAATCTTCCTTGAAATCGAAGTGCTGCCGAAAATTCTCTCTGATTTCTATTTCTCTCTCAATCTCTAAAGGTCGTCTCTCTCTCTCTGCAAATTTCGATTCACTCAAATCAGGGGCGAAATTGTTGCGCTGCTGAATTCGGTGAAATTGTTAGGGTTTTCCATCTTTGCTCAACCTGAAGCGCCATTCCATATCTGGTTTGATTGCTTCAAGGTAAACCCTTTTCTTTCTTTGTCATAAACCCTTCAATTCACCACTTTTTGATTGATAAATTTGCTTGTTGTTGCTGTTGCTCAACCCTTCAATTCCTGAATTATCTGCTTGTTTTTCTGGGTTTCGGGCTGTTGTTGATGTACTTCTCATCACTCGCCAGATTTGTATTAACAGAATTACAGAGTGTTGTCAAAGATTGGGTTGTTGTTGAAGTAATTAAACCCTTGAAGATTTGTATTAACAGATTTGTACACAGTCCTCAGCCTGCTGGGTTTATATCAGATTGGAGGAAGCCACCTGCCTGCCTGGGATCCCAATTTAAATTGGAAGAAGTCAGAAGCCAACGGGCTCTTGAAATGAATTAGAGGCCATAGGCAATATTGTTCTGTTGTGTTATCTCTCTTTTCTGTTTTTGTCCCCGCTTCCAATTTATCATCTAGGAAGAATCAATCTTTTGCCTTTTGAACCTCATCAAAAGAAAAAAAAGAATGAATTTTGTCCTTTTAATGTGCTAATTCCCAACGTAACAAACGTTTTGCTGTCTCAACCAAGACTGGAAAACAAGGGTTGATGTGCTCTGTTGAACTCTTCATTTTTTTGGTTTGATGATGTGTATAACTTGATATCGATTGGTTTAAGTGGTTCTTCTTGCTAGGCTGATTGAGAAAGTTCTGAACTTGTTGGGCTAGTTTATACAGAAGGAAGATGGGTTCTCGTTTTTGGACTCAGGGTGACAGTGACACTGAGGAGGAGGAGGAGGAGGTGAGTGAATACAGTGATAGTGAAGGTGAGGTCACTCACGGTGGAGACTCTGGTGCGCCTGCTGCTGGAAACAAGTATCTGCAATCAGATGATAGTGACAGTGATGACTCGGATGGCCAAAAGCGAGTTGTTGTTTCAGCCAAAGATAAACGGTTCCAAGAGATGAACGCTACTGTTGAACAGATAAAGAATGCCATGAAAATCAATGACTGGGTGAGCCTGCAGGAGAGCTTTGATAAAATAAATAAGCAACTGGAAAAAGTCATGCGTGTTGCTGAGATGGGCAGGGTCCCTAATGTCTATATAAAAGCTCTTGTTATGCTAGAAGATTTCTTGAATGAGACCTTGGCAAACAAGGAAGCCAAGAAAAAGATGAGCAGCAGCAATGCCAAGGCATTGAACAAAATGAAGCAAAATCTGAAAAAGAATAATAAGCAGTATGAAGAGCTAATTGCCAAATATAGGGAGAATCCTGAGGTAGAGGAGGAAGAAGAGGAGGAAGAGGAATCTGAAGGGGATGATTTTGGAGAGGATATTGAGGATCCCTCTGATGCTCTTGGATCAGAAGCAGAAAGTGATGATGATGATGATGACAAAGGAGGTGATCAGGACCTGCTGGATGATGATTGGCAGAAGAAGCTGGGGAAGAAAGAGAAGTTGATGGAGAAACAGTTTATGAAGGACCCAAGTGAAATTACTTGGGATATAGTCAATAAAAAGTTTAAAGAAATTGTTGCTGCTCGTGGTAGAAAGGGTACTGGAAGAGTGGAGCTTGTTGAGCAGCTCACCTTCTTGACTAGGGTTGCAAAGACACCTGCGCAGAAGCTGGAGATTCTCTTCAGTGTTGTGTCTGCACAATTCGACATCAATCCTAGTCTCAGTGGGCACATGCCAATAAATGTGTGGAAGAAATGTGTTCAGAATATGTTGATCATTCTTGACATTCTCACTCAGTACCCAAACATCTTGGTTGATGATACAGTTGAGCCTGATGAAAATGAAACCCAAAAGGATAAAGACCATAAGGGTACAATTCGAATATGGGGGAACTTGGTGGCATTTCTTGAGAAGATAGATGTTGAGTTTTTCAAGAGTTTGCAGTGTACTGATCCTCATACACGTGACTATGTTGAGAGGCTGCAGGATGAGCCTCGGTTTTTGGTACTGGCCCAAAATGTCCAAGAGTACTTGGAAAGAAGTGGCAATCTCAAAGCAGCTGCAAAAGTAGCCCTGAAGCGTGTTGAGCTTGTGTACTACAAGCCTCAAGAAGTATATGATGCCATGAGGAAATTGGCTGAACTGTCAGAGGGGGGAAATGATGAAGCCACAGGTGATGAGTCTAAAGGGGCTGATGAAGTTAAGGGCCCTCCGTCCTTTGTTGTAACCCCTCAGCTTGTGCCTCGGAAGCCTACTTTTTCGGAGAGCAGTAGGGCCCTGATGGACAGCTTAGTTTCCCTCATCTACCAGTATGGTGATGAGCGAACTAAGGCTCGTGCAATGTTGTGTGATATATATCATCATGCGCTAATGGATGAGTTCTCTACTGCTCGCGACCTGCTGTTAATGAGTCACTTACAAGATAACATTCAACTTATGGATGTCTCAACCCAAATACTTTTCAACAGGGCTATGGCTCAGCTTGGTCTTTGTGCTTTCAGGAGTGGACTGATTTCTGAAGCACATGGTTGCCTTTCTGAACTGTATTCTGCTGGAAGGGTGAGAGAGCTTCTTGCCCAAGGTGTGTCACAAAGCCGCTACCATGAGAAGACTCCGGAACAGGAGAGGCTAGAAAGAAGACGCCAGATGCCGTACCATATGCATATCAACCTGGAACTCTTGGAGGCAGTCCATCTGACTTGCGCAATGCTGCTAGAGGTTCCTAACATGGCTGCCAACACCTTTGATGCCAAGCGCCGTGTTTTAAGCAAGACTTTCCGTAGACTTCTAGAAGCGAGCGAGAGGCAAACATTGACCGGTCCACCCGAGAACGTCCGGGACCATGTCATGGCTGCATCCCGAGCCTTGATCCTGGGGGATTTTCAGAAGGCCTATGATGTCATCACGGCTCTTGATGTCTGGAAACTTCTGAGAAACCGGGAGAATGTCCTCAATATGCTCAAGTCCAAGATCAAGGAAGAGGCTCTGAGGACGTTCCTTTTTACCTATGCCTCTTCATACAACTCCCTGAGCCTGGACCAGCTGGCTAAGATGTTCGACCTCTCAGAGACTCAGACCCACAGCATTGTCAGCAAGATGATGGTGAATGAGGAGCTGCATGCCAGCTGGGACCAGCCCACGCGGTGCATCATCTTTCATGAGGTTGAGCATACCAGATTGCAGGCTTTGGCATTCCAGCTCACTGAGAAACTGTCAGTCCTCGCTGAGAGCAATGAGAGGGCCATGGAGGCCAAACTAGGAGGTGGTTCATTGGATGGTCTGCCACTGAGGAGGAGAGAAGGACCAGACTATGCTGCTGTTACTGCAACTGGCGGTGGTAGGTGGCAGGACAACTCGTACTCCCAGGGAAGGCAAGGAGGCCGCTCGGCATATAGCGGGAGACCATTGGGTCCAGGTCAGGTCAGCGGAGGAGGGTATTCGCGAGACCGTGGTGGACAGTCAAGAGGAACAGGGGGGTCTTACAGTAGGGCGTCCATGAGGGGCCAAATGGATTCATCTGCCCGTATGGTCAGCCTTAACCGTAGTGGAGTTGCCCGTTGATCTCGTTAAACTTTCAATATTTCTCTCTTTAAGTGTTGCTTTCGAGAATATAGCGTGATTTTTGCTTATGACTTTTTTGAGGTTAGGTTACTTTGAATTGTGAGACTGCTGTGTAATTAGGTTGATCTTTGTGATGAACTCTAGCATATTAACAGGTACTATTCACCACTTCTGGCATTGCTGCTGTCTTTCCATTGATGTGTTTTTTGGGTCATTTGGGACGTTTGCAAGATCGTTGTCAGCGCCCAGGCAATTCTCCGATGGTTTCACTGAATGTGGG

*GAPDH*

ATGCACTGTTGTGGACGCGCTACGCCCCCCCCACCCCTCGAGAAAATCTATTTAGTTTTTCTTGGCTTTTTTGGCCTCCCTGTCATTCTCTCTCAGATCACCCTCCTTCACTCTCTCGCTCTCTCTCTCTCTCTCTCTCTCCCTCCTCTTCTCTTTAAATATAAAACGTAACCCTACTTTCTCTCAGTCCGCTCTTCTACTCTCCACTAACCCTTCTAGAAAATTCTTAAACAACCACTCTCCGCCTCTCCTTCATCATCTTGCAATGGCAGCAGCAAAGGTCAAGATCGGAATCAACGGATTCGGAAGGATCGGCCGTTTGGTCGCTAGGGTGATCCTTCAGAGGGACGATGTTGAGCTTGTTGCTGTTAATGATCCCTTCATCACTACCGAGTACATGACATACATGTTCAAGTACGACAGTGTGCACGGACAATGGAAGCACCATGAGCTCAAGGTCAAGGATGAGAAGACACTTCTCTTCGGTGAGAAGGCAGTCAGTGTCTTCGGCATCAGGAACCCAGAGGAGATCCCATGGGCTGAGGCTGGAGCTGACTTCGTTGTTGAGTCTACTGGAGTTTTCACTGACAAGGACAAGGCTGCTGCTCACTTGAAGGGTGGTGCTAAGAAGGTTGTCATCTCAGCACCTAGCAAGGATGCTCCTATGTTTGTTGTTGGCGTTAACGAGCATGAGTACAAGTCTGATCTCAACATCGTCTCCAATGCTAGTTGCACCACAAACTGTCTGGCTCCTTTGGCTAAGGTTATCCATGAGAAATTTGGCATTGTTGAGGGTCTTATGACCACTGTACACTCTATTACTGCCACCCAGAAGACTGTTGATGGTCCGTCAATGAAGGACTGGAGAGGTGGAAGGGCTGCTTCATTCAACATCATCCCCAGCAGCACTGGAGCCGCCAAGGCTGTTGGCAAGGTTTTGCCTGCTCTTAATGGGAAATTGACTGGAATGGCTTTCCGTGTTCCTACTGTTGATGTTTCCGTTGTTGACCTCACAGTCAGACTAGAGAAGCCTGCTAGCTATGATCAGATTAAGGATGCTATCAAGGCTGAGTCTGAGGGTAAGATGAAGGGAATTTTGGGATACACTGAGGATGATGTTGTTTCCACTGACTTCATTGGTGACAACAGGTCAAGCATCTTTGATGCCAAGGCTGGTATCTCTTTGAATGACCACTTTGTGAAGCTAATCTCTTGGTATGACAATGAAGTGGGATACAGTACTCGTGTAGTCGACTTGATTGTGCACATGGCAAAGACCCCGTAAAGTACTGTTGAAGAGAGGTTGGGCCCGTGTTGAAGCAGTTGTTTTCCTTATATATGAGATGGGTATGAGCAGAATAAAGCAGGAGTTTGAGTCACTGTTGAATGTTGAATGTGTGAATTCGAGTGTCATAGGTTTGCTAAACAGGGGTTTGAGTCATCTCCCCTTGCGTTCTGTTATTGATTACCAAGTAAGGCCGTAGTGGACGGTTACTGATCGAGCTGGTAATTTTTGCTTCTCTACTTGCCCAGTATTTACGATGTTGACTGTGGGACCGAGTTTTGTACGTGTATGTATCCTGCTATGTATCCTTTTCATTGTTCCAAGTTCTTGGTTCTTTGTCAAAATGCGTGGTAACTATGCGATCTGGGTGTTGAAAGTGTGGGTTCATTTGGCTCTAAGACTTGAAAGAAAGCGAAATGCCTGGTGGACTGTGAACTGTTAACTAATATCTAGGATTTTTTTTTTGACAATTTGTAGCTGAGGTCTCCCCTCTGTAGTCTGTGGGACAGAAACCAAAGAGAGGATAAAATAGGTTCTAGCGACTGCCCACGTGTGCTATC

*histone(1)*

CTCATCTGTAATCAGCTTGCCCATTCTCTCTCTAGAACGTTCTCTTGCTAACTGTCGTCGTTCTCCAGAAGCTATCTCTCTCATCAAAAATGTCTGGCCGAGGTAAGGGAGGCAAGGGATTGGGAAAGGGAGGCGCAAAGCGTCACCGTAAGGTCCTCCGAGACAACATCCAGGGGATCACCAAGCCGGCGATTCGCCGTCTCGCTCGTCGTGGTGGTGTCAAGCGTATCAGCGGTCTGATCTACGAGGAGACTCGTGGTGTGCTCAAGATCTTCTTGGAGAATGTCATCCGTGATGCAGTCACATACACTGAGCACGCAAGGAGGAAGACGGTGACAGCCATGGATGTCGTGTACGCACTCAAAAGGCAAGGCAGGACTCTGTACGGGTTCGGTGGTTAGAGAAATTGGAGAATTTCGGATTTCTAGGGTTTCTGGAGAAGGTTCTCGTGTTTTATTTGAGGTTAGGGTTTCGTAGCTTTTGTGGATCTGGTTGGTTTTAGTCGATGGTTCTTCGTTTAGTTATGTCAGCAGTGGTGGTTTTATGTAATTTAAGTTTGTACGAAGTAATGAATATCTCATTCTTACTAATTTCTGTGATT

*histone(2)*

AAATGCCAGTAAGGATCTCAAGGTAAAGCGTATCACTCCTAGGCATCTGCAGCTTGCTATTCGTGGAGATGAGGAACTTGATACCCTCATCAAAGGAACCATTGCTGGAGGTGGAGTCATTCCTCACATCCACAAGTCCCTCATTAACAAGTCATCCAAGGATTAGACTTCCAGTGATTATCTCTGGGTTTTCTGCATTTTCATGTGAGACCTCTGGAAGAACATTTGCAGAGAGAAGTCAAAGGCTGATAGAGGAGGACATTTGATTAGGTTTTGTGTTCTTCATTTCAATTAGCCACAGGATGTTGAATATGCATTTGCTAATGTGGATGTTCTAGGTGATCTTTTAGCTGTCAACTTTTAGTTTGTGCATCTTTGATGTTTAATTCAGGGGCTTCGGCCCCCATACCAACCCCCCCCCCCCCCTAAAAAAAAAGGGAGAATCTATGTCTAGTGGTTTAGTATTATGACGCTGTGTGCAGTTTATGATGATATGATCTGTCATT

*histone(3)*

TCAAAATCCCTAGTTCTCCCTTCAGCTCTCCTCGCTTCTAAAACCCTTCTTCAAAACCCTACCGCTTGCCATGGCAGCCATGCAATTCTGGGGCATTGAAGTTAAAGGAAAAGAGCCTAGCAAGGTGAAACTTGAGGAGGGCCATCTCATCCACCTTTCACAGGCTGCTTTGGGTGAACAGAAGAAGGATGGTGAGCCTGTAATCCTGCATGTGAAGGTTGATGGCAAGAAGCTAGTCCTGGGCATCTTGAGCTCGGAGAACAACCCACAGTTGTCTTTTGATTTGGTGTTTGAGAGAGATTTTGAAATCTCTCACAACTGGAAGAATGGAAGTGTTCACCTCTTGGGATACCAGTCTGAGTTCCATGACGAGGGGGATGACTATGTTGATTTCACTGATGAGGATGAGGATATGCCTATTCCACAAAAAGAAAATGGAGAGGCTGCTGCTGCAGTTACGAAGGCTATTGCTGCAAAGCCAAACAAGGATGATAAACCAAAGCCTGAGGCAAAAGCTGAGGATGATGATGATGACAGCAACGATGATGAAGATAGTGATGATGATGAGTCTGATGAGGATATGCTTGATATGAATGATGATAGTGATGATGATGAGGAGGATGGTGAGGATGATGATGAGAGTTCAGAGGAGGAAGAACCCGCCAAAGCTGAGTCTGGGAAGAAAAGAAAGCCTGAGGCAAAGACACCTGAGCCTAAGAAGGCAAAAACAGCTACACCCCAGAAAACAGATGGAAAGAAGGGTGGACATACGGCAACACCGCATCCCGCTAAGAAAGGTGGAAAGACTCCTGGTGGAGATAAATCTACCCCCAAGTCTGGTAGCCAAGTTTCCTGCGGTCCTTGCAAGAAGACCTTCAACTCAGAAGTTGCTCTGCAGTCTCACACAAAGGCCAAGCACAGCGGCAAGTAGAGTGGGCATTTTTTCTTTGCCCTTTAGTTTGTGTACGATGATTGATGGAGGGGGGGGGGGG

*histone(4)*

AATCATCTTCCCCCTCTATAAAGCCAACCCCAAAAACCCTAGCTTCCCCAACCTCATTTCCACAACAAACCAAATCAAATTAAATCCAACCTGAGATTAAAATCCAAATCAACCGACCCAGAAGATCCATCGAGGACAGAGATAATGTCGGGAAGAGGAAAGGGGGGAAAGGGGTTAGGCAAGGGCGGAGCGAAGAGGCATCGAAAAGTGCTCCGAGACAACATTCAGGGGATTACGAAGCCGGCGATACGGCGGTTGGCACGGCGTGGCGGCGTCAAGAGGATCAGCGGCCTCATCTACGAGGAGACTCGAGGTGTGCTCAAGATCTTTCTGGAGAATGTCATTCGCGATGCTGTTACTTACACCGAGCATGCTCGCCGGAAAACCGTCACCGCCATGGATGTGGTGTATGCTCTGAAACGTCAGGGTCGTACTTTGTATGGATTTGGGGGTTAGAGAGAGAATTTGATGTGTAGATTTGAGATTTAGGGTTTAGTTTGAAGTCGGATTTTAGGAGTTGGATGTAATTATGGTATAGATTTCTGTGTTGGTGTCAATGAAAATGCTGAATTGAAGGCGAAATTTGGTTGTTTTTGTCAACTTCAATTCTGTTCTTTTTGAAGTTGAATGTAATTTTGTTTTGTTGCCAAAAAATTAAGCAATTATGTTTAATGGTGAGTGTCAACGTACGTAATTGTCATGATGGATGACGAGGTATATTTTTAATATACAT

*histone(5)*

AGAGAAGAAGAAGAAGAAAAAATCTAGTCTTTTAGTTTTACATGCCAAGAATTAAGTTTAGTGAATGGTGGTTTTACCAAGCAAATTTCGTCTCAGAGAGAAAATGGCAAGATCTGTGCAAAAATGATGCCTTTGTATGGAGCCTGTTGCATTAAAAATTTTGAAAAAAGATAGAGAAAAAAGAAACCCTTTTTTAATTAAGGTAGGTGTATGAAAGAAGGGTGATGATGGGGAGTTGCAATGTTTTGAACACGAAATGATCTAAACCAGTTCCAATTGGCTAGTTCAATGGACACAAAGATGGAAACAATTGCTCAAAATGGAAATGACCGGATTGTATGCGGGGACCTCAATGGAAGGTGTGAAAATGTGGAGCTGGCGAGTGGCTGCCATTGTTCTGATGCAAATGGCAGAGTAGGCAATATATCAGTCATGTCTACTGAAGGCATTCCCCAGAAAAAGGCAAGGCTTAATGGAGTGTTGACGCTGGAAGATATCTATAACCTTCCAGATGAGTTGGATGATGACGAAGATGATAGTGACTGGGAGCCGTTACTGGAGCCTCTGGCAGTTAGGAAATGGTTTTGCACCAACTGTACAATGGTCAACTTCGACGGGTTTGACTTCTGTGAGACATGTAAGGAGCACAAAGAATCTGGAATCCTTAAGCATGGTTTTTTTGCTTCGCCAGCTTTGCAAAGGACCAGATCCACCCAAATTGAATCAGAAGTAATAGAAAGATATACAGAATCAATCTGTGATATATCTGCATCCGCGTTGTCCACCGTTGTTGGTTTTGATGAGAGAATGTTATTGCACTCAGAAGTTGTATTGAAGCCACATCCACATCCTGAAAGGCCTGATCGTCTTCGAGCCATTGCGGCTAGCCTTTCAACTGCAGGAATATTTCCAGGAAAATGCCATCCAATTGCTGCAAGAGAAATAACACAAGAAGAACTCCTTAAGGTGCATTCTCTGGAGCATGTAGAAGCTGTTGAAGTTACTCGCCAAATGCTTTCTAGTTATTTCACCCCTGACACATATGCAAATGAGCATTCAGCACAAGCTGCAAGGCTTGCGGCAGGTCTATGTGCTGATCTTGCTTCCGAAATCTATTCAGGACGTGCCAAGAATGGATTTGCCCTGATTCGGCCGCCTGGTCATCATGCTGGAGTCCAACAGTCTATGGGTTTTTGCCTTCACAATAATGCTGCAGTTGCCGCATTAGCTGCGCAAGTTGCTGGAGCCAAGAAGGTCTTGATTATAGATTGGGATGTACACCATGGAAATGGAACTCAAGAAATATTTGAAGGGAACAAGTCGGTTTTGTACATTTCACTGCATAGACATGAAGGAGGAAAGTTTTATCCAGGAACCGGAGCAGCTCATGAGGTTGGTACAATGGGTGCTGAAGGCTATTGTGTTAATGTACCTTGGAGCCGCGGGAGAGTTGGTGATAATGACTACATTTTTGCATTTCAAAATGTTGTAATTCCAATAGCACACGAGTTTTCTCCAGATTTCACCATTATATCTGCTGGATTTGATGCAGCAAGAGGAGATCCGCTTGGCGGTTGTGATGTAACACCTGCTGGCTATGCTTGCATGACTCACATGTTGAGTACTCTTGCTGGTGGAAAGATGCTTGTAATACTGGAGGGCGGCTATAATCTACGTTCAATATCATCCTCCGCCACAGCTGTCATTAAGGTATTACTTGGTGAAAAGCCCACGTGCCAATTTGAGAACATTGAACCCTCCACATCTGGCTTGCAAGCGTTGCTGGAAGTGCTGAAAGTGCAGACAAACTTTTGGCCATGTCTTAGTTCTAAGCTTACACAACTGCAGTCATGTTGGGAAGCATACTTGTCAGGGAGAAAAAAGCAGAAGAAAAGGAGATTCCGAACTGTGGCACCACCACCTATTTGGTGGGCATGGGGAAGAAAAAGGTTCTTGTATTTTCTCCGTTGCCAGCGGTTTCGCATGAAACCCTAGCCATGGTTACAGACACAAACACAATGTAATGCTAACCAACCCCATCATAGTCATAGTTGCCCCCTTTTGTTTGATCTCTCTGCGGGGCTCTGGGAAGCTAAATGTTGTTTAATCTTGCATTCTACCTGCTTCATGAATGTGACTTGTCCATGTAATTATACGCCTAGGTGCAATCAGTTTGAGTTTGCC

*TATA(1)*

TCCCAGCAACCCCCTCTTCTCTCTCTCTCCTCTGATATAATCCTCTCTCCTATCTCCGTCATATAAGACCTCCATCCTTCAAATTTCTCATCTCCATCTCATATAAAACACTATCCTTTTCAACTTCTTCGCGCTTGTTGCCTCAAATCCAACACTGTAAAGCTCGGAGCCCTTCATTTTTCATTTTGATTCTTCTTCGCTGAATAAGCAACCCTAGATTTCATTTTGATAGGTAGATCCGTCGATCTTCTTTGTCTAATCGGCGGATCTTTTGTCATAACGAAAATCTAATTGAAGAATCTTAAGTAATGTTGTCAATTTGTCATCATTAGGTCTAAATCGAAACCCCTTCAAGGAATGGATTAGGTTTAGCTTTTTCGCTTTTAGGGTAATTTTGAACGGGGTTGGCGTTTAATTGATGAGGATTTTGGTTTGTGATTGGTCAAATTAGTGTGATTGAAGAGCATAAAGGAAGGGAGGGAGACGGTTTCAGATAGAGAACAATGGCAGAACAAGCCATGGAAGGAAGCCAGCCCGTGGATCTGGCCAAGCACCCATCTGGGATCGTCCCTACGCTCCAGAATATTGTCTCAACGGTCAATTTGGACTGCAAGTTGGATCTCAAAGCCATTGCATTGCAAGCTCGTAACGCAGAGTATAATCCCAAGCGTTTCGCTGCTGTAATCATGAGGATAAGGGAGCCAAAGACGACTGCGTTGATATTTGCCTCCGGGAAAATGGTCTGCACTGGAGCTAAGAGCGAGCATCAGTCGAAACTGGCTGCTCGTAAGTATGCGCGCATCATTCAGAAGCTGGGTTTCCCTGCCAAATTTAAGGATTTTAAAATCCAGAATATAGTAGGATCTTGTGATGTCAAGTTTCCTATACGACTGGAGGGGCTTGCATACTCCCATGGTGCTTTCTCTAGCTATGAACCGGAACTATTTCCTGGCTTAATTTACCGAATGAAGCAGCCCAAGATTGTGCTACTCATATTTGTATCGGGCAAGATTGTTCTTACAGGAGCTAAGGTGAGGGAAGAGACCTATACTGCCTTTGAGAACATATATCCAGTGCTCACTGAGTTCAGGAAAAACCAGCAATGATTTGTGGGAGGAGTTGCTGAAGATGTGTGCCCTCCAGGAGCTTGATGCCCTTTCATCGTGTGAACTGTAAATAGATGAAAGGAGATAAAGATATAAGGCGCTTGCCCTGCCGTTTGGATTGAATTTATGTACTAGCGTTGTATGCCTATGCCTAGTTGTGGGGACTGGATCATGTATGACCTGGCCGAAACACGATCGTCTAAGTCGCTGACTCAAATGACCATCTAAAAATTTTGAAAGACTATTCGATTGTCTGCTGATTGTAGGTTGCCTCAATTTAAACTTCAAACCTAATTTTTGCTGTTAATTTTAGCCTTTGCTGACGATGTGTTCCTTCCATATTTCACAAAAGACGAAAACAGAGTTATTGACTTGTCTTCCCGTTTGCAAATCACAATGTTTCTCTTGTTTGCAACTGTCTTTCAATATTTGTTCCGTGTTCTTC

*TATA(2)*

TGTTTCTCTCTCCTCTCTCCCCCCCACCAATATTAGCTTCGATCTTTCGTGAGTTTTTTCCTCTTTAGTATATAAAAGCCCCCCTTTTCAAATTCTTGGCGCTCAAATTCTCACTTGAGATTTCTGAATCCTCTGTTCTTTGGGGGCTTAAATATCTTCCTAAACCCTAGATTCGTCCGGGGGAGGCGAGGCGATTCGAGAATGGCGGAGCAAGGGTTGGAAGGAAGCCAGCCGGTGGATCTGTCAAAGCATCCTTCTGGGATCGTTCCTACACTGCAGAACATTGTCTCGACTGTTAACTTGGACTGCAAGTTGGATCTTAAAGCTATCGCTTTGCAAGCTCGTAACGCGGAATACAACCCAAAGCGTTTTGCTGCTGTAATTATGAGGATAAGAGAGCCGAAAACCACTGCATTAATATTTGCGTCTGGAAAAATGGTTTGTACTGGAGCCAAGAGTGAGACTCAGTCAAAATTAGCAGCTCGTAAGTACGCCCGCATTGTGCAAAAGTTGGGATTTCCTGCCAAATTCAAGGATTTCAAAATCCAGAACATAGTGGGATCTTGTGATGTTAAATTTCCCATCAGACTGGAAGGGCTCTCATATGCTCACAGTGCCTTTTCTAGTTATGAACCGGAACTTTTCCCGGGTTTGATCTACCGAATGAAACAGCCCAAGATCGTGCTGCTTATCTTTGTATCTGGAAAGATTGTTATAACTGGAGCAAAAGTGAGAGAGGAGACGTATACTGCCTTTGAGAACATATAT

*TATA(3)*

CTCAATTCATAGATGTTATTATGTTAATAAGCAAAGAGCTGCTCTGAATCAGCAAATCAGTATCTTGAGAGCTACAAAATCTTGTAATTAAATAAGCAAAGAGGATTTAATGGCTTCTGCTACATCTGCTCTCTCAGTTTGTAGTTCATCATCATGTGTTAGAAGGAGCAAACGAACCATGTATTCTAGCACTTCTTCTTCACATTCATGCCTCAAAGCCCAAAAAACCCTGAATTTTGATCTCTCATCCAAGTTTTCTCAAATGGGTATGTGCCATTTTACACCTTGGAATGGTCTGAGACATGTGGGCAGCTCAATCTTAGCAAGACCTCAGAAGCTGGGAAGCAGAAAGAAATGTAAAGGGATGGTTGTTTATGCTTCTTTGTTTGGAGTTGGTGCTCCTGAACTCTTGGTGATTGGGGCTGTAGCTTTGATGGTTTTTGGTCCTAAAGGACTTGCTGAGGTTGCTAGAAGTCTGGGTAAAACTTTACGTGAATTCCAACCAGCCATCAAAGAGCTGCAGGATGTATCCAGGGAATTCAAAAGCACCCTGGAGCGGGAGATTGGACTTGCTGATGATCCCAGTGCTGTGAGAAAGACAAATGAATCAAGCAGATCTGATTTGACTTTGAGCCAATCAGTTTCCACCTCGGACAATCAGATCACATCCATGGCTGAAACTAATGGAGCTCCACTCAACAACGCATATTCAGGCGAGCACAACTCGAATATCACAAAGGAGCAAAAAATTGCAGCTGCACAACAGGACACCAAATCCTCGCCACCAGAAAACCAGACTGAAATCAAGGGTCAGCCTCAAGGTGTCCTACCTGACAATGCAAATTCTAGTGAAGATCACTTGAAGATCACTGAGGAGCAACCAAGGGTACCTGTAGGTACACAACAGGATGTTACATCCTCACCACAAGATAGCCAGACTGAAATTAAGAGTCCGCCACAAGGAGCCATTGAAGAAACTGCCACAGCAGCACCTGCAGTCCAGATACCTGAAAGTGAGACATGAGAAATTCATGGGACACCTTGTATGTTATGAAAATGCTTCAGCTGAGAAATTGACCAGTGCATATTCAATGTGGGAAAACGAGTGCGTGTGTTTAGCATCTAATTGCTCATATGCTGGATGATGATGACTATTGCTAGTGCTTGTTATGAGTGCAGCTGAGTATGTTTTAACCAATATTTGAACTCTGGCACTTGAGGATAAGATCATTTTGCTAATGAACAACACCTTTGAAGAGTTATGCGGTGCAATGTAGTTC

*TUA(1)*

TGATGCCTTCAACACCTTCTTCAGCGAGACTGGAGCAGGGAAACATGTTCCCCGTGCCGTCTTTGTTGATCTGGAACCCACCGTCATCGATGAAGTCAGAACTGGAATTTACCGCCAGCTGTTCCATCCAGAACAACTGATTAGTGGGAAGGAAGATGCGGCAAATAACTTCGCTCGTGGGCACTATACAATTGGAAAAGAGATTGTCGATTTGTGCCTTGATAGAATCAGAAAGCTGGCTGACAACTGCACTGGGCTCCAGGGCTTCCTTGTATTCAATGCTGTTGGTGGTGGTACTGGCTCAGGTCTTGGATCTCTTCTGCTTGAGCGTCTCTCAGTGGACTATGGCAAGAAATCCAAGCTGGGTTTCACTGTTTACCCATCGCCCCAAATCTCAACTTCCGTTGTGGAGCCCTACAATAGTGTGCTCTCTACGCACTCCCTCCTTGAGCACACTGACGTTGCAATTCTTCTTGACAATGAGGCTATCTATGATATTTGCAGGCGCTCCCTTGACATTGAGCGCCCAACTTATACCAACCTCAACCGTCTTGTTTCTCAGATCATCTCCTCGCTCACAGCCTCGTTGAGGTTTGATGGAGCCTTGAATGTGGATATGAATGAGTTCCAAACTAACTTGGTCCCCTATCCAAGAATCCATTTCATGCTTTCCTCCTATGCCCCTGTCATCTCAGCCGAGAAGGCATACCATGAGCAACTCTCAGTTGCTGAGATCACTAACAGTGCCTTTGAGCCCTCTTCTATGATGGTCAAGTGTGACCCCAGGCACGGCAAGTACATGGCTTGCTGTCTCATGTACCGGGGTGATGTTGTCCCAAAGGATGTGAATGCAGCTGTGGCCACCATCAAAACGAAGAGGACCATCCAGTTTGTGGACTGGTGCCCAACTGGGT

*TUA(2)*

CCATAATTTGTGGTACAATTACAAGATAGTTATTACTTACAAATCTCTCTTATAACCAAATAAAATAATAAAAACACCATTACAAATTTGTTTGCATTACAAGTTTGTTTACATTCAATCAAATATTATCTTAAAGTCACTGCCTGAAAATCAGGTTGAATAAAAATGGATGAGGGAGGAGAAACCAAGCAAAACAGATACCTAATGAAAGAAATAGAGCACATGATACCGAGACATTTATACACAGAGAGTGCAAAGAACAGCAGAAATTCATGTGTCATTTTCATATACTCAGAAAAAAATAGGCCACCCTAAACCAATCAAAACAACATTGTGACATGAAACAAGCCATATTCAAACTATCATCCACTCAGCGCCAAAAGTAAACAAACTATGCACCGGAGAAAATTTAGCAACACGATACACAGTGGGCAACAACAGGAAAAGACACAGGAAACCTGGAGACATTGCCGAACTCTCCCAACAGCACTGGACCTCCTGAATTGTGAATTTGTGATGCCTACCCCCTTCAGTATTCCTCGCCTTCATCCTCATCTTCAGCACCCTCAGCCCCGACTTCCTCGTAGTCTTTCTCAAGAGCAGCAAGGTCTTCCCGAGCTTCAGAGAACTCGCCTTCTTCCATGCCTTCACCGACATACCAGTGTACAAATGCCCTCTTGGCATACATGAGGTCGAATTTGTGGTCAATTCTGGCAAAGACTTCAGCCACTGCTGTGTTGTTGCTGATCATGCAGACAGCACGCTGCACCTTCGCAAGGTCGCCTCCTGGCACCACTGTTGGGGGCTGGTAGTTGATACCGCACTTGAAACCAGTAGGGCACCAGTCAACAAATTGGATAGTTCTTTTTGTTTTGATGCCAGCAACGGCAGCATTGACGTCCTTGGGTACAACATCACCCCGGTACATCAAACAACATGCCATGTACTTCCCATGTCTTGGGTCGCATTTCGCCATCATGCTAGAAGGCTCAAACACAGCAGTTGTGATCTCGGGCACTGAAATCTGCTCATGGTAAGCCTTCTCAGCAGAAATAACCGGGGCATAGGATGACAGCATGAAGTGAATACGAGGATAAGGCACAAGGTTAGTTTGGAACTCTGTAACATCCACATTGATGGCTCCGTCAAACCTCAAAGAAGTTGTCAACGAAGAAATGACTTGGGATATTAAACGGTTCAAATTGGTGTATGTTGGTCTCTCAATGTTTAGGGATCTCCTGCATATGTCATATATAGCTTCATTGTCGAGAAGCACAGCAACATCGGTATGCTCCAAGAGAGAATGAGTGGAGAGCACGCTATTGTAAGGTTCTACAACAGCTGTTGAAACCTGAGGAGAAGGATAAATTGTAAATCCCAGTTTTGACTTCTTTCCATATTCTACGGATAGGCGCTCAAGAAGCAATGACCCAAAACCAGAACCAGTTCCACCACCAACAGCGTTGAACACCAAGAAACCCTGTAAACCAGTACAACTGTCGGCCAATTTCCGCACCCGATCAAGGCACAAGTCAATGATATCCTTGCCCACTGTGTAGTGTCCCCGAGCAAAGTTGTTGGCAGCATCTTCTTTCCCAGAGATGAGCTGCTCTGGGTGGAAAAGTTGTCTATATGGTCCGGTCCTGACCTCATCAATCACAGTTGGTTCCAGGTCAACAAACACAGCTCTAGGGACATGTTTCCCGGAGCCGGTTTCGCTAAAGAAGGTGTTAAACGAATCATGTTCAACACCTACAGAAGTGTCACTAGGCATTAACCCATTAGGCTGAATATCATGCTCAAGGCAGAAAAGCTCCCAACAAGAATTGCCCACTTGAATTCCAGCTTGACCAATATGAATGCTGATTATCTCTCTCATTTTCTACCTCTTTTTCTCTTCCTCTTCTCACCTACAGCGACAGAGATCTAGATCAGCCAAGACCCAGAATTCTTCCACCTCTCTGTCTCTCTCTCTCTCTCTCTGTGTGCCTTTTAGCTGTCTGTTTCTCTCTCTAGGATGGCAGTTTCTAGAGAGGGAGAAAGCAGGGAGAAAGAGGGCCGGTGCAAGGGGACGAAGTGGATGTGCTGTGTTCAACGTTGACAGACAGGCCTTCGTTAACTGTTTCCTTTATATTATACCTAAAAACCGATGGAAACTACTCACTCACTTTTA

*TUB(1)*

CGACTATGATGAGAAGGGGTTGCAGGTCAAGTAACTTGTTACTGAATATGGGTCCATTGTCTAACAATGGTGGAGTGCATCACTTTAATTCGCAGAACGCTAAGGGTTAAAGAAGACAGCTTTCATAATCCTGACATGGGTACCTGTAGGCTGAGCTGAAATGCCAATGCACTATACTAACAGACATATTTTTCTTTTTCTAAAATACTTGGGGGCAGGGGAGGTGCCATTTCAGAGGATAGCCATTGCTACAAAAACGTGAAGGCTTTGAGCAAATCAATAGATTAAGATGCAAGACTGACCAAATCAATAGCATTTTACTTGAAGAAAGGGAATTGCAAGGATTTTAAAGATACAAACAAAGAACAAATTGAAACAATTGAAGCAATAGGTTTGTGCCGAGCTACTCCATCATACTCTGCAAAGCAAATAGCATCTCTAACCCTCATAGTTATCGTCAGCACCTTCCTCATACTCTTCCTCATACTCTGCAGTTGCATCCTGGTATTGCTGGTACTCAGCCACCAGATCATTCATGTTGCTCTCTGCCTCTGTGAACTCCATCTCATCCATACCTTCCCCAGTGTACCAGTGCAAGAAAGCCTTGCGCCTAAACATGGCTGTAAATTGCTCACTCACCCTCCTGAACATCTCCTGAATGGATGTGGAGTTGCCCACAAAGGTTGAAGCCATCTTTAGGCCAGTTGGTGGGATATCACAAACACTTGACTTGACGTTGTTGGGGATCCACTCTACAAAGTAGGACGAGTTCTTGTTCTGAACGTTGATCATCTGCTCGTCCACCTCCTTCGTGCTCATCTTTCCTCTGAACATGGCTGAGGCTGTTAGGTACCGACCATGGCGGGGGTCAGCAGCACACATCATGTTCTTGGCATCCCACATTTGCTGAGTCAGCTCGGGGACAGTCAGGGCACGGTACTGCTGGGACCCGCGTGAGGTCAGGGGAGCGAACCCAACCATGAAGAAGTGGAGACGGGGGAAAGGGATCAAGTTCACAGCAAGCTTGCGGAGGTCAGAGTTGAGTTGACCAGGGAAACGAAGGCAGCACGTAACACCACTCATGGTGGCTGAGATCAAGTGGTTCAAATCACCAAAGCTGGGTGTGGTTAGCTTCAGAGTCCTGAAGCAAATATCATATAAAGCCTCATTGTCCAAAACCATACACTCATCTGCATTCTCCACGAGCTGGTGGACTGACAGGGTAGCATTGTATGGCTCAACCACTGTGTCTGAAACCTTTGGTGATGGGAAGACAGAGAATGTCAACATCATCCTGTCTGGGTACTCCTCCCTGATCTTTGAGATCAGCAATGTACCCATACCCGAACCCGTTCCTCCACCAAGAGAGTGGCAGACCTGGAACCCTTGAAGGCAGTCACAGTTCTCAGCCTCCTTCCTCACAACATCAAGAACTGAGTCAATAAGCTCAGCACCCTCAGTGTAATGTCCCTTAGCCCAGTTGTTACCGGCACCGGATTGTCCAAAGACAAAGTTGTCAGGACGGAAGATCTGTCCATATGGGCCAGTCCTAACGGCATCCATGGTACCGGGCTCAAGGTCCATCAACACAGCACGGGGAACAAATCTTCCGCATGAAGCCTCATTGTAGTAAACATTAACACGCTCCAATTGCAAGTCAGATGTGCCGACGTATCTTCCAGTAGGGTCAATGCCATGCTCATCACACACAACCTCCCAAAATTTAGACCCAATCTGGTTACCACACTGCCCACCCTGAATGTGAAGGATTTCTCTCATTTTTCCTGTTAGGAAGAAGATCCGAAGTTAGAGAGAGAAAGAGATAGAGATAGAAAGGGACAGAAATAAGACGGCAATGAATTATGAATTGAGAAATAGAGAATTTGGGATTGAATTTGTTTGGGGGCTTCAGCTTTTTGGGTAAGAAAAAAGGAGGAGACAGATGGGCAACAGTATGCAACAAAGGCTTTAATTAGAAGCAAAATACAAGCCAGAAGGATGAAAAAGGAAGGAAGCAAAGCGGGGGTTTTTCTTTTCTCTCTCTTGCTTTATATGTGTATGCACACTGTGGAGAGAGAAAAGATTACTCGGTTACTCCAGCCTTGAAGCCGGTGGTGGTGGGTGGAGGTTGAAACCGTGTGGTGTGTTGTGTGACATTGTCTGGTTTTGAAGAGGGGAATCTTTTATCATTGCTGATCAATGCACATTTGGGTCATCTGCATTATTACAATCCATTGATCATTTATCTAGCCGTTACGTACCTTTGGG

*TUB(2)*

CATCATGTTCTTGGCATCCCACATCTGTTGGGTCAGCTCGGGTACTGTCAAGGCTCGGTATTGCTGGGAGCCCCTCGATGTCAAGGGGGCAAACCCAACCATAAAGAAGTGGAGACGAGGGAATGGAATAAGGTTCACGGCTAGTTTCCTCAGATCGGAGTTCAGCTGACCGGGGAACCTAAGGCAGCAAGTAACACCACTCATGGTTGCAGAGATAAGGTGATTAAGATCGCCAAAAGTTGGAGTGGCAAGCTTGAGAGTGCGAAAGCAAATGTCGTAGAGAGCCTCATTATCAAGAACCATACATTCGTCAGCATTTTCAACTAGCTGATGCACCGAGAGGGTGGCATTGTAGGGCTCAACAACGGTGTCAGACACCTTCGGGGAAGGGAAGACTGAGAACGTCAACATCATCCGGTCAGGGTACTCCTCTCTTATCTTGGAAATAAGAAGCGTACCCATACCAGAGCCAGTGCCTCCACCAAGAGAGTGACACACCTGGAATCCCTGAAGGCAATCGCAATTCTCGGCCTCTTTGCGAACAACATCAAGAACAGAATCAATGAGCTCGGCACCCTCAGTGTAATGGCCCTTAGCCCAATTGTTACCGGCCCCAGATTGGCCAAATACGAAGTTATCGGGTCGGAAGATCTGGCCAAACGGGCCGGACCGGATAGAGTCCATGGTACCCGGTTCGAGATCCATGAGAACAGCCCTTGGGACATACCTTCCACCGCTGGCCTCGTTGTAGTAGACATTGATGCGTTCTAGCTGTAGATCTGAGTCTCCGCTGTACCTTCCGGTCTGATCAATGCCATGTTCGTCGCATATCACCTCCCAGAACTTGGCTCCGATCTGGTTCCCGCATTGCCCGCCTTGAATGTGAAGGATCTCTCTCATGGTGTCGTCCCTTTTGGCGGGTGGGTTCGGGATCGGAGAGCGAGAGACAGAGAGAGAGAGATGGCGGGACTGCTAGCTTGGGCAGCGGATGTGGTGGGAGGAGGTGGTGGTAGCGACGGAGGATCAGACGATCCAGATTCGATCCCACTCATTCTCACCCCAGAACAAGAGCAGTATGTCAGAGAACTCAACCGGAAGGCGGCTTCTCTCAACCGTTCGATCCAGGATCTACGGCTCAGAATCCCTCCCTCTGACATCTCCCAGCGTCTTCCTCACCTTCATGCTCACTCCCTCGCTTCCAATGCTGCCCTTGCCCTTCAATTGAATGCCCACTCTGCCACTCGTGAGCAGGCACAACAAAGAGAGGCAAAGTTACTGGAAGAAAATGCGGCTTTTGAAAAGGAAATATCAATTTGCCAGGCAAAGATTCAAGAGAAGTTACAGGAGGCCGAACAGTTTCAGAAGAAGTTGAAGGAGATGGAATTGACAGAGGAGATTCTCAGAGCAGAGCTGGAAAAGGCTCAAGCTGCTTCAGACACCAGTGATTCTAACAAATCTATGTTTGAGCCTGGACATGGATTTGAAAACAATGCTGAAGAAGATGCTTCCAAGGCTGCTCTGATAGAGAAGTTGGAGAATAAGAAAAAGGCATTGCACTCGGTGGAAGAGAAAATTCAACTTCTGGAGAGGAAGTGGGCAGAGATTCAAGATAAAGTACTGAAACAACCTTCTCCAGCTCAGAGAGAGAAAGCCCTGGATAAGCAGCTTCATAGCTTAATAGAGCAACTAGCTGCAAAGCAGACACAAGCTGAAGCTCTCGCAAGCGAAATACATTTGAAGGAGAAGGAGCTGGAAAGATTGAACAGGATGTGGAGGACAGTTGAGAATAGCAGGGAAGCCAACAATGCCAGGAACCGGTTGGGAAGAAGTGGATCAAGCGGCAGGTTTGACTTAGCTGACAGCGAACTAGATGGTCATAGCAAACTCCCATATCATACAGGTGGTCGGTCTGAATACCGACAGAGGCTTAGGCTTCTAAGATCATCGTTTGTGCTCTACATCTTTTTGCTACATATTGTTGTCTTCATCAAGATCTCATTCTGAGAGGGTTGATGCTTGCATGCTTCCTTTTGTAATCGGTTCAGTGAAAATGAGATCATTTGTGCATATATGACTGAGTAACTCGCGCGGAATAAGCTTGACCTGTATTATCATTATGAGCCTTGCAATGGATCTTGATATTTGGAAATACGCCACGATTCCTTCATCCCTTGAGCTAGTTGAAAATATAAGAGAAATCGTATTATTTCTTATTCTTCACCCATCCAAGACTGTAACACAGCCTTAGTTGGGGAGGCGTGCGAGGCAAG

*UBC(1)*

TTTTTTTGCATGAACAATGGAAAGACTTTTATTCCCGACGACAATAACAGCGAGTATTGTTCCTCCTGCAAATTGCAATCCTGCTGATTCAAACAGCTACAGACTGATCAAACAGAATTCAAGTGGAACTGCCAAATCATGGGTCTTGGAAGAAAGGAAAGCATACTAAAAGTACAGACTCCAATTGCTCAGTTTACTAGTGTTTTCACATTCTGCAAGTATTGTAAGTCAAATCCAAATTACATGAAAACACACAAAACCCGATCCTACACAGTAACAACTAACAAGCAAGCGTCTCAGGTCCTTTTTTGCGCCCTCCTCACAGCACTTACACGATTAATATTCAAGACGACACAGTAAACAACAATCAAGTTGTACACAATATATAAGACAATGAGGTTGCCTCTTTTCAAGCGTTCTGAATCCCGAACCATGATGGGGAATGGCTAGAAGTAGGTCCCTTCTGGAGGTTGAACGAGCTTTCGGTTGTGAGGACAAGCCATTTCCTTTCTAAGCTGGGTCAGAATGTCCTCCATGGTGAACTCCCTCTGCCAATTTGTAAGCATCCCAACCTTCTTTGGTTCAACCACTCCAGTCTCAGGATTGACACAAGTCATGTTGATCCGTGTGTGGAAGCGAACGCTTGGTGGCTTCTCGGGATAATCTTTGTCACAAAAGAGCTTCAACTGATAAATGCGGCCTTCATGTACAGTATTGTGAGAGCCAATTATTGTTCCAGTCCACGAGCGCATGTAAATATCATCACTATCATCCATCCCATAGCTGACACTGCCATCTCCAGTCCCCTTCTCACCGCGTTCAAGCTCCTCCAACAACCTGAAGTTCCTCGGAACGACGACGCTGGATCCTCCAGAACCAATCGTCATTTTCCGGCTAGATCGAAACCGAAAGAGGTTGAAGACGGAGCTGCGGGGACGATCGCAAGAGAGAGAGAGAGAGAGGTAAAGATGTCGATGGTGCGGATGACTTGTTTTGAAGATTTCTCTCACTTTGTGGATTCCAGAA

*UBC(2)*

GAAAGACAATAGAGGTTGAGAGAATCCCCAATCTCCCCAATTTAAAGTCATACCTTGCCCTTTTCTACTTCTTCTTCCTCCTTTTCCTTCCGCCGGAGAAAAGGAAACCTAGCAATCTCGAAACCCTAGCTGCGTTGCAGTTGGCAGAGGAGAGGGACTCCTACGATCTTCTCTTTTTCAATCTCAGGTCAAGGGGATGGCTTCGAAGAGGATCTTGAAGGAGCTAAAGGATCTGCAGAAGGATCCTCCCACTTCATGCAGCGCCGGTCCCGTCGGTGAAGACATGTTCCATTGGCAAGCAACGATTATGGGCCCTCCTGACAGCCCTTATGCTGGGGGTGTTTTCCTTGTTACCATTCACTTTCCCCCAGATTATCCATTTAAACCCCCCAAGGTAGCATTCAGGACAAAGGTATTTCACCCAAATATAAATAGCAATGGAAGTATCTGCCTGGACATCTTGAAGGAACAGTGGAGCCCTGCCCTTACCATTTCTAAGGTATTGCTTTCCATCTGCTCGCTGTTGACGGATCCTAATCCTGACGATCCCCTTGTGCCGGAGATTGCTCACATGTACAAGACTGATAGAGCTAAATACGAGAGCACAGCAAGGAGCTGGACCCAGAAGTATGCTATGGGCTAACTACTCATCTGGTCATGTCACCTAGGGGAGGGTCAGAAGGCATTGTGGTTTCTGATAACTACTTTCTAAGCATTCTGTAAGTGGGTGATGAATCTTTGATTATGTGTGTCTTCGATCCTAAGAATGGACAAAAAGAATTGTCCACAAATTTCTCTGTCAAAAAGAGAGAACCTTTATGTTGCCCTTTTAATGTTTGTATGGTCAAAACTTAATTTTCAACCTGTGAAATATGATTCTAATCTGGTGTCACAACATCTTCGTCGTCACTTGATAACCCCCCCAGCCCCAACATCAACTTATCATCAGGGAACAACATGAACATATGTTCTGTTTGGAATTTGATGGTTATAAACAAATTTTGCTTTCAACTATATTGAGCTCTGATGCAG

*UBC(3)*

ATCAAATTGAACCCGAAAATCAGTAGAATGTAGTAGATGATTTTTGCATAAATAGACAGAGTACATTTATTTACAGGATGGAGAAGGATTAAACAAATTAAAGATATTTTCCCCAACAGTCTAAAGTCTTTACATTAGAGCTGGGGTTTTCAATTTTGAAGCAGATCTAAGGGATGATGATGTACAGATACAAGAGGAATTGAACAGGAAATAACTAGGGATCTGGTTGACCCACCATAGCTTCATCACTGGAAGCATACTCGTCTTCACTCAGCTCCTCATCGCTGCTTTTTTTCTTCAGGCACAGCTCCTGCATCCTCTGGCTTGGCATATTTCTCACAGTATTCTTTGACTTTTCGCTCATATGCAGCTCGGTCACGCATCATTAGAGCAGCAGCCTCTCCATTCAAGGGGTCTGAAGGATTAGGGTACAAAAGAAGCTGTGGGAGGAACACCTCAAAAACATTCACCAAATCAAACATTGGGCTCCAAGTCTGATTAATGACATCAAGACAGACTGAGCCTGACATCTCATCAACATTGGGATGGTACATCTTGTTAATAAAACCTATTGATGGAGATTTGTAGGGATAAGCATCAGGAAGTTCAACCCTAATCCTCCACACACCTCCTTGATAGGGACTGTCTTTAGGCCCATGAAATTCCACATAGAACTCTTGGACACCATCATTAATCATTTCCACCTTGTAATCACTCATCATCAATTTCATCAAATCCATTTCTCTTCGCTTGCTTGGGGACGACATGGTTGATGTTACTTGCCAAAGCAGTCACCTTCCGAG

*UBC(4)*

GGGGGGGGGGGCGCCTGGGCGGAGGAAGGGAGGCAACTCCGGTTCCTGATATTTTCCGGGTAATCTTTTGATGATATGAAAGTCTACCACGGCAAAGATCACATGCTACAGACCTCACAGAACCTACACCCAGCACTATCTTCCTATTGGTCCACATCATAGAACCCAGAAACCCACCCGCAACAGCAGCGGGAACTCACGCAGGTTTACCCGCATTGAATAATTCCTCAACCTCCCTCTCAGCGGACGACTCATAAATAACCCCCTCCCCTCTTTCCCTCTCTCTCTCTATGCTTTCGGTCTTCCCTTTTGGGCCCACCCCACTTTCTCTCTCTAACTTGTTTCCCTCTTTCTTTCTGTGTCGTCGTCACTGACGAATTTGCATGAGTGTTTACCAGGAGGCTCATCGGAAGCACAAAAGATCCGATTGCTTTTCCGGCCAACTTTCTCCCGCCGCCATGGATATGGAACTCGATGATCCCCCTTCTTCTGATTCTCTTGTCGCCAAAAAGCTCAAGCAACAACAAGACTCCTTGACTAGTGATCATGAACTCCCTGCTGCTGTGGTTTCTATGGATAGTGTCAAACCAGTCAAAAGCTCCACATCAGGATCCAATAATTCAGCTGATCATACTGGTTCCACTTCTGACTTATCATATCAGGATGATGCCAATGATGATTTGGATGATTACATTGATGATGCTGACGTTGTTTCTGATTATGATGATGAGAATAATGAGTATGTTGATGATTTGGGTGATGATGATGATGATGATTACTTGAAGCTGCAAGCACAATTTGATAATGTGGATTTGCCTCCTGGAGTAGAGGCATCAGTTCCCTGGTTGCAGGATCCTTCTTCAAGTGAGGACAAGGCGACTGAAACTTCAATGTTGCCTGGAAACAATGTAAAAATGTCGGGTGCTAGTAGTTCAAAAGTAGAGTCAAGTCCAAAGGCTAAAAAGGAAGAAGAAATGGATGATGTTTTACGGAAGTTAATGTACTTTAAACAGTTTGATACAGTTGCTGATTGTTCTGACCATTACTACCTGGACATGAAAAATACGAGCCGGCAGCCGTCAAAGTCTTGGGCAAAGAAAATTCAGGATGAATGGAAGATTCTAGAGCAGAACTTACCGGAGACTATATACGTGAGAGTATATGAGTCACGGATGGATCTTTTGAGGGCTGTCATTATAGGACCCGCCGGTACTCCGTATCATGATGGGCTTTTTGTCTTTGACGCTGTCTTCCCGCCTTCATACCCAAGTGATCCACCGATGGTCCATTATCATTCGAGTGGCTTTCGTCTGAACCCCAACCTTTATGAATGCGGAAAAGTCTGCCTGAGTCTGTTAAATACTTGGCATGGTGGAAGACAAGAGATGTGGCTTCCAGACAAATCAACAATGCTTCAAGTTTTGGTTTCAATTCAAGCTTTGATCTTGAATGCGGAGCCCTTCTTCAATGAGCCTGGGTATGAGAAGACATTCAAGGGTGCAGAGGGGAAAAGAAAGTCCAAGGAGTATAGTGAAGACATTTTTATTAAATCCTTGAAGAAAATGATGTACACCCTTAGGAATCCACCACAGCATTTTGAAGATCTGGTGGCTGGTCATTTTCGCGTTCATGCCTATGATATATTGACAGCATGTAAGGCATATACTGAAGGTGCTGAGGTAGGTTGCAGTATTAAGAAATGGCTTGAAGAAGGCGACAATGCTGAAAGGCCTGGCTCTACAAACTTCAGGCAGGAGGTTGCTAAGATGATGAAGCCACTGCTTAAATACTTCTTGAATAACGGATCCAAGGACTGTGATAAGTTTCAGACTGATGCTTAGTTCATAGACTGCCGGATTGAACAGCGATTACTGACATAAGTTGGTGTTTTTTGGTTTTGGGTTAATTAAGGCAATCTGTCGCAGTTCACTGACTTTCATGACAACGTTAAGTAGGAATGGTAATGTGGTGTACAGTTATTGATGGCTTAAATAAATTTGCTCATTTCCAATGTTCAGGTCTCTACGTTTTCAAGCTTTTTTCATGGTTGCTACAGTGCCACTTGCTTACTTGCTTACTACGCCGTGCAACAACAAAGTGTAAGGAACTGG

*UBC(5)*

TTAATCCTTTGGAGAAGAAGATTCTTCTGAACAAAACAGGCAAGATGGAAGATGGAGAAAGACAATTAATTCAGGGGGTTTACTTAAAGATTTAACAATAATTAACCTTATTGACAGAGAATATATATGAACCCATTAGGTTCCTCTGGCCCATGCACTATTTATTGGAGCTTTTTGCAAAGAGAAATCGAGTGGGAGTGAGAAAGGGAAAGAGCGATCCCATCCCAAGTTGCGGATTCGCAAGCAACATCTCACTTCTCGAATCTTCCCTTTGAGCAATCCCAATCCCAAATCCCAGTCCCCTGTTAATTGTATCGCTCTAAAGCTAAATCTAATTGGGGTTTCTGTAATTTCTCTCCTTTCATGGCTTCACCATCTCAAGCATCTCTCTTGCTTCAGAAGCAGCTCAAAGATCTTTGCAAGAACCCAGTTGATGGGTTCTCAGCCGGATTGGTTGATGAGAACAATCTATTTGAATGGAGTGTCACAATTATTGGGCCCCCCGATACCCTCTATGAGGGAGGTTTTTTTCAATGCCATTATGAAATTTCCACCGAATTATCCCAATAGCCCACCAACGGTGACGTTTACATCAGAGATATGGCATCCCAATGTTTATCCGGATGGAAAGGTTTGCATATCAATCCTTCATCCGCCAGGTGATGATCCAAATGGTTATGAGCTTGCAAGCGAGCGGTGGAGTCCTGTTCATACAGTGGAAAGTATAGTGCTAAGCATCATATCGATGCTTTCTGGTCCTAATGATGAATCGCCAGCAAATGTTGAAGCTGCTAAGCAATGGAGGGATGATAAGGAAGGGTTCAGGAAAAGAGTCAGCCGTTGCGTAAGGAAATCCCAAGAAATGCTATGACGAGTGATCATCATACGTCCTAAAAGTTGTTTCATCGTCTTTCTTAGCTTTATATGTTGATCTCGCCATTGTGCTCTGGTCCGAAATGAAAGGAAAGGGGATTAATGATCCAACTCAACTGCTTTAATAGAGATGTATTGTGGGGTTCTGGCATGTTCTTGTTTGATTCATGAATTGTGCTTTGTGCGTTTTGGAGTTGAAATGACAGTGTGTCTCTGTTGCAAATATTTCAGAGTCTGAATGGACAGTCCATGGAGTATCAAAGGGTTTCATTGTCCTGTTCTATTTTTGCAGTTGATTAAAATGTGTCGAGTTGGGTTAAGTATTCATTTCTGTGGCCATGATCTGTGGAAAATCTGAAGCAAAACAGGCACTCACACACATATTCTTCAGTTCAAAATGTTGTCAAAGTATGAAAAAATGTACTCCATTTTAAGTCGATATCATTATATCAACAGGTGAAATGGTCCATTGTTGGTGTGTCTTGGTGTCGATCATGAGTTGTTTTGTCAGTGGTTATGCTTTCCAATTCTCCAGACCCCAATATTGTCAGTGGCTATACTTTCCAATTTGCTAGACCCTGAATACGCGAGACCTAAGGTGGGAGTAGTTTAGTAGTTTGCTTTCTTGAAGCGTATGTGCTATTTTAGTTGATCTCGG

*UBC(6)*

TCTCTCTCTCTCTCTCTCTCCTGACTGTGCACTTCAGATAAGATAACTCTGCTGAAGCCCCTATCATCTGGAAGCGGAAGTTGTCGGAGCTTGGTGCAGAGTCAGGAGGAAGCTCCACTTTTCAGCAAAGCTAGTTATCATGGCTACTAATGAAAATCTTCCGCCTAACGTTATCAAGCAACTTGCCAAAGAGTTGAAGACTCTTGATGAAACACCTCCGGAAGGAATCAAAGTGGGGGTTAATGATGATGATTTCACCACCATATATGCAGACATTGAGGGCCCAGCTGGGACCCCTTATGAAAATGGCGTGTTCCGGATGAAGTTGATATTATCAAGTGACTTCCCTCATTCACCTCCAAAAGGTTATTTCCTTACCAAGATTTTCCATCCAAATATTGCGAACAATGGAGAAATTTGTGTGAATACCCTGAAGAAGGATTGGAATCCGACTTTGGGATTGCGTCATGTTCTTCTGGTGATAAGATGTTTGCTCATAGAACCTTTTCCTGAATCTGCTCTAAATGAGCAAGCTGGCAAGATGCTTCTTGAAAATTACGAGGAGTATGCAAGGCTTGCTAGGTTATACACTGGAATTCATGCGAAGCCAAAATCTAAGCTAAAGCAAGGAGCCATCTCTGAGTCAACTACAGCTCTGAATGTAGACCAGAGGAACACCACCACAGCTCAAAACCCTGACCAGAAGAACCCATTATCGAGCACTGGAGCTCCCTCGCCATCTCCTGTTGCCCCATCAACGGCTAGCAGCAAAGGAACGAGCAGTCAGGAGCAACCCGCAGCCTCTGTGGGGTCCACTCTAGTTTCAAATGAGACCGGAGGTGTGGGCTCATCGGCTGCACCGCCCCAAAAGAAGGATGGGGCAGCATTGAAGGCCCAGGCGGAGAAGAAGAAAATGGATGCAAGGAAGAAGAGCCTCAAGAGATTATGATCTTATCCATCCAATCAATTGGCAAAGTGTGGTGGCTGTCGTAGTTTCTCTTAGGCTTCCCCCTTCCCCCAGAAAAGGTTTTTTTTTAAAAAAAAAATTGAAAGAAAATTGAAGGAAGAGGGAACTGGCGATTGAGAAGTTTGGCCGGGTGGTGAACCCTAAGCTCTCATAGCGATCGGGAGGAGGAGAAAATATCTTGACAAAATTTCAAGCCTTTGAAGCTGCTTCTTCCTTTCAGAGAATAGAGATGAGGTCTAATGCGACTGGAGTAGGATACTACAACTCTGAACACAGGAGCTTCAATCGTGGTTTTGGGCGTGGACATCCCAGACCTTATCAATCATCTCAGCGTCAGCCATCCTCACAGCCAACCCGCAAGGGCGATCTTTTCATTGAGGCTGGCCGTCTTGCTGTTGAGTATTTGGTTTCAAATGGATTGTTATCTTCGAATGTGCTCTCAGGAAAGTGCCAGAATGGCACCTTGAGAAGCAGCTCCGGAGAATTCCGGGATCATCGAGGTTCTCACGATAGAGAAAGAATTTCAAATTCGCGTGTAGCTGATCGATCAACCGGAACATCTTGTCAAGGAGCTGCTTCTCCTGATGGAGTTTCGGGTGCTAGGAAGACAACTGATGATGAAGATCCAAGAATGTCTAGGAATTACTCTGGGGAAAAAAGAAGACTTGGAATATCCAGGAGTTACAGCTCTGACTGGAACCGTGAAAATGGAAGAGAAGGGTCTTTTTCTGATAGAGTGACTGCTTCACAGGGTATAGATGCTGAAAATGAGAAGTTTCTTGAGGGAAAATCCAAGGATGCAGATGCCGATAACTTGAAAGATTCTGCCTCTAAAGATGAAAGATACAGCGAGTTGCAAGGTGAAATGGGAAAGTCCCATCATGTTGATTCAGATTCGAAGCTGACTTCGTCACTTCAAAGCAAGGATGAAAAAGAGCTCTCTGAGCCTTCTGATATGGTCAATACTGGATGCAAGGAGACAAAAGATGGGACCGGGGATTCTCATGTGGATATAGAGACTTCCAAGCATGAGTTGACTAATCAGGATTGTGAAGAGAAGAATGATACTTCTAGCAAGAGTGGTGTGGATCTGCTTAGGTTATGCCCTTCTGCTAAGATTCCAACTAAAATTCGTTCCTCCTTGATGAATAAGAGTCCGAAGAGTGATCCACTTTTAATCAGTGAGGAAGAAAAATACAGTGGATATGATTCTATTGTGTCGATAAAAGATGATACCATGGATAGTTCCTCTGTAATGAATAGTTCCTCTGTAATTCTATCTTCAAATGAGGTCCCTAGTTCAGATTCTGTTGGTAATCATTCTTCAAGGGATGGAGATGACCTACGTGCTACTTATGTTGGAAGTCAGTCAATGCCTGTGGAATCAGTTGCATGTACTGAGAAAACATTCTCTTCTGAGCAAAAATCTGATGGGCTGCCTGAAATTGAGAGGTCCAGTTCCATCACTGGTGATAGAGGTGAAAAGCGAGCTTTTGAGGAAGACAATATGGGTGAGGGAGCCAAGAAAGCTAAAATGTGGCTTTCTGTTGGTCAGAGTGATGACTACTCACAGCTTTCAGATCTGAGTGAAAGGCTGTCTATTTCGCTGGCCAAGAGAGTGCCTTCTGCTAGCATTCTTAGTGAGTGTTCTAATCATGAGAGCCTCACCATGTCCTTGCGTCTAAAAGGCAGTGTTGAATCAGCTAAAGAACAAGGGGAGGAAAAGCAGCTATTCTCAAACTCATTTAAGATTTGTGATCTTAACCTTATGGAAGCTTCTGATGTGCGCGAGAATCGTGATATGCCTGTACTCATGTATCCCTCTATCTCTGCTGCTAGTAAAGGTTCACTAGCTGGCATTGATTTGTCCATGAATAACAATTGCGGTTTGACTAACAGCTACAATGATTGTGGTGCTGTGAGAAAAGACATTGAAATAATTGATCTGGAGGGCAGAACCTTCAAAGAAGGGGAAAGCATGAATGCTTCAGCGACAAACACGGAAGCTGGCATTATGAATGTGGGGAGCTTTCAGAACAGTTCACATGACTCTAATCATAACCCAGATGGTCAGGACGGGTATGGCTTCATGATCTCAGAGCTGCTTGGTAATGATGTTCCAAATTGCTCCTCAGTTCAACCAGATATGAATTCTTTGCAGAATGAGATGGGGGTGCATCATGGGCAGGGAATTTTTGCTGAAGATGATCCAATCTACATGTCTTTTGGAGACATTCCATTGAGTCTTTTGCGGGTTTGGGATCAACAAACACAGGAGAGAAATCCTTTTGAAATGTAGCTCCATAGAATCACATGTTGCTGCTGTCGACACAATCAGTAGCAGCAAATAGTTCACAAAGATCATGGGACTCTAGAACCACAGACTTGGCAGCTATCCTCACGTAGTAGATGCTAGCAAATGTCAGA

*UBQ(1)*

GAAGGTATGTAGAAGAGGAGGAGGGCAGGAGTGGAAATGTGGGCGGCGACGCTCTGTGGCGGCGGCGGGTGTCGGCATAAGGTGGCGGTGAGGAAGGTGGGGATGGTTGAGGATATGGACGTGATTTGGGTTCAGACCCAGTTAGAGAATTTGAGGCAATCAGCAATGTGGTGTAGGAATGTTTGTACTTTTCATGGGGTTATGAGGATGGATAATAGTCTTTGTCTTGTCATGGATAGGTGTTATGGGTCTGTTCAATCCGAGATGCAGCGGAACGAGGGCAGATTGACGCTTGAGCAAATCCTTAGATATGGAGCGGATGTTGCTAGAGGGGTGGCAGAGCTTCATGCTGCTGGAGTTGTTTGTATGAATTTGAAACCATCTAATCTTCTTCTTGATGCAACTGGTCATGCGGTGGTCTCTGACTATGGACTTCCAGCAATCCTGAAGAGGCCCACTTGTAGGAAAGCTCGAGCAGAAGGGGATTCCTTAACAATCCATTCTTGCATGGACTGCACGATGCTCAGTCCACACTATACCGCTCCAGAGGCGTGGGAGCCTGTTAAGAAGTCCTTGAATTTGTTCTGGGAGGAGGCTATAGGTATATCTGCTGAGTCAGATGCATGGAGTTTTGGCTGTACTTTGGTGGAGATGTGCACAGGAACTATTCCCTGGGCAGGTTTGAGTGCAGAGGAAATATATCGAACGGTTGTGAAGGCTCGAAAATTACCTCCACAGTATGCTAGTGTTGTTGGTGTTGGGATACCGAGGGAATTATGGAAGATGATTGGGGAATGCCTGCAGTTCAAACCATCCAAACGGCCTACTTTCAGTGCAATGTTAGCTATATTTCTTCGTCATTTACAATCCCTGCCTCGCAGTCCACCTGCAAGTCCTGATAATGATTTGGAGGCAACTGCACCTACAAACATGATGGAACCATCCCCAACATCTGTATTGGAGGTCTATCCATATAATCCAAATCATCTTCATCTTCTAGTATCAGAGGGGGATTTGAATGGTGTAAGAATTTTCCTTGCAAAGGCTGCTTCAGGTGCTGGTAGTAGTGCAGTGTCTTCACTGCTAGAAGCACAGAATAGTGATGGCCAGACTGCTCTTCACCTTGCTTGTAGGAGAGGGAGTGCGGAACTTGTCGAGGCTATCTTGGAGTACAAAGAGGCTGATGTGGACATTCTTGATAAAGATGGGGACCCTCCACTAGTTTTTGGTTTGGCGGCTGGTTCTCCAGAGTGTGTGCGTGCATTGATAAGAAAACATGCTAATGTGAGATCAAGATTGAGGGAAGGATTCGGCCCTTCACTTGCTCATGTCTGTGCATACCATGGCCAACCTGATTGCATGCGTGAATTGTTAATGGCTGGAGCTGATCCCAATGCTATAGATGATGAAGGTGAATCTGTGCTACACAGAGCTGTGGCTAAAAAATATACCGACTGCGCTATTGTTATACTAGAACATGGAGGTTCACGATCAATGGGCATTCGGAATTTGAAGAACATGACACCCTTGCACATCTGCGTAGCAACATGGAATGTGGCTGTTGTGAGGAGATGGGTGGAACTTGCTACCTTTGACGAGAGAGCTGATGCAATAAACATACCCAGCCCAGTAGGCACAGCCTTGTGTATGGCTGCAGCTGTGAAGAAAGATCATCAAAAAGAGGGAAGAGAATTGGTGAGGATATTGCTTGCTGCTGGGGCTGATCCAACAGTCCAAGATGCTCAACAGCGCACTGCTTTGCATACGGCTGCTATGGCTAATGATGTGGAGCTGTTACAGATAATTCTTCACGCCGGTGTTGATGTCAACATTCGCAATGCTCACAATACAATACCACTTCACATGGCATTGGCTCGAGGTGCAAAATCCTGTGTTGGCCTGCTTCTGTCTGCTGGCGCCGACTATAACTTTCAGGATGATGAAGGTGATAATGCTTTTCACATAGCCGCTGATGCAGCTAAAATGATCCGTGAAAATCTTGAATGGCTTATTCTAATGCTCAAGTATCCAGATGCTGATGTTGATGTCAGAAACCACAGTGGCAAGACCTTAAAGGACTTTCTGGAGGCCCTTCCACGAGAATGGATTTCTGAAGATCTGATGGATGCACTTTTGAACAAGGGGGTGCAATTGTCTCTGACAATATATGATGTTGGTGATTGGGTGAAATTCAGAAGAAGTGTGACGACTCCAACATATGGATGGCAGGGTGCGAAACACAAGAGTGTTGGTTTTGTACAAAGTATCCCAGATAAGGACCATCTTGTCATTTCATTTTGCACTGGAGAGGCTCGCGTTTTGGCAAATGAAGTTATAAAAGTGATTCCATTGGATAGAGGGCAGCATGTGAAGCTTAAATGGGATGTCAAGGAACCAAGGTTTGGATGGCGTGGTCAATCACGTGACAGCATTGGAACTGTTCTATGTGTGGATGATGATGGGATATTGCGGGTTGGGTTCCCTGGAGCTTCCAGAGGATGGAAGGCTGATCCTGCAGAGATGGAAAGAGTAGAAGAGTACAAAGTTGGGGACTGGGTACGTATCCGTCCAACTCTTACTACAGCTAAGCATGGTTTAGGAGCTGTTACTCCAGGTAGTATTGGTATTGTGTACTGCATTAGACCAGATAGTAGTCTGTTACTGGAGTTGAGCTATCTACCAAATCCATGGCATTGTGAGCCAGAGGAGGTTGAGCATGTCAAGCCTTTCAAGATTGGTGACCGGGTCTGTGTCAAGCGCTCTGTTGCAGAGCCGAGATATGCTTGGGGTGGTGAGACTCATCATAGTGTGGGAAGAATAAGTGACATAGAGAGTGATGGCCTTTTGATTATTGACATTCCCAACCGGCCAATTCCATGGCAAGCTGACCCTGCTGACATGGAAAAAGTAGATGATTTCAAGGTAGGAGATTGGGTAAGAGTCAAAGCTTCTGTTTCCTCTCCTAAATATGGATGGGAAGATGTCACCAGAAATAGCATTGGAGTCATTCATAGTTTGGAGGAGGATGGGGACATGGGTGTTGCCTTTTGCTTTCGAAGCAAACTCTTTTGCTGTTCTGTGACCGACATGGAGAAGGTGGCACCTTTTGAAGTTGGACAAGAGGTTCATGTGATGCAGTCTATTGTGCAGCCTCGACTTGGTTGGTCTAATGAATCTCCTGCTACGGTTGGAAAAATCGCGAGAATTGACATGGATGGTGCCTTAAATGTGAGAGTAGCTGGTAGATCCAGCTTGTGGAGAGTCTCCCCTGGAGACGCTGAACATCTATCAGGCTTTGAAGTGGGTGATTGGGTGCGATCAAAACCTTGCTTGGGTACCAGACCAAGTTATGACTGGAATGCTATAGGCAAGGAGGGTGTGGCAGTTATTCACAGTGTACAGGACAATGGTTATCTAGAGCTTGCTTGCTGTTTTCGGAAAGGAAAGTCAATTACTCATTACACAGACGTAGAGAAGATGCCTCGTCTCAAGGTTGGACAGTATGTCAAATTTCGTGCTGGACTAGCAGAGCCAAGATGGGGGTGGAGGGGCACCAAACCTGATTCCCGAGGCATTGTCACCAGTGTCCACGCTGATGGCGAAGTAAGAGTAGCATTCTTTGGCTTGTCGGGGCTATGGAGAGGAGATCCTGCTGATTTGGACACAGAGCCTGTGTTTGATGTGGGTGAGTGGGTAAGATTCAAAGATGATGCTGGAAACTGGAAATCTATCAGGCCTGGAAGTATTGGAGTTGTTCAGGGTCTAGGATACGAGGGAAACGAATGGGATGGAACGATCTTTGTTGGTTTCTGTGGGGAGCAAGAAAAGTGGGTGGGTCCGAGCTCTCATCTCGCGAGAGCTGATAAGCTGATACTTGGACAAAAAGTAAGAGTGAAATTCTCAGTGAAGCAGCCTAGGTTTGGCTGGTCTGGGCATAGTCATGGGAACATTGGGACAATAGCAGCCATTGATGCTGATGGCAAGCTAAGAATCTACACCCCAGCGGGCTCGAAGACTTGGATGCTGGACCCATCTGAGGTTGAGGTAGTTCAAGAGGAGGAGCTCGGGATAGGGGACTGGGTTAAGGTCAAGGCATCGGTGATAACCCCTACTCATCAATGGGGAGAAGTGACCCATTCGAGCATTGGGGTAGTTCACCGGATGGAGGAAGGGGAGCTCTGGGTGGCTTTCTGCTTCATGGAGAGGCTTTGGCTTTGCAAGGCGTCGGAAATGGAAAAGGTGAGGCCGTTTAGGGTTGGGGACAAAGTGAGGATCCGTGATGGGCTTGTGGCACCTCGATGGGGATGGGGGATGGAAACTCATGCTAGCAAGGGAGAAGTAGTTGGGGTGGACGCCAATGGAAAGTTGAGGATAAGGTTTAGATGGAGAGAAGGAAAACCGTGGATTGGAGATCCTGCTGATATTGTTCTAGATGAGGCCTAACCGATGTAGAGAACTGAGTATCCTACCCCCCACCTCCTCTAAAACTCTCAGGGTGACCCTACATTGCCTTCCAGTCTTCCATGGGCCGTGTGGAAAAAAGAGAGCTATTTTTCAAGCAGCCTCAAGATAATTTCACCCATCAAAGGTTCTCAGAGGATGCTAGCCAAACTGATCCCAGTGGTGCCTTCCCTGCGTCTGTTCTCTACTCTTATCAGCATGTCATGGGTGGTTCGTTGGCAGAACTCGTTGACTACTAATAACAACGATGGCAGTCGAGGAGGCGTGTTTATGTGGGCTTGTTTTCAATGTTTGACAGGTAAATATTTGAGCTGAAGAAGGGAAGTTCAGCAGTATAAGATTTGTATTTCAGTTAAAGGCGATTTCAATAGGCCTCTCTGAGCTGAAGTAAATAGAGATAGATATATATAGAAACAGAGAGCTGGCACTTGTACAAATTGCATGTCCACCATTATCTTTCAAGTTATTGTTTTTGCTTCACTTTTTGTGGTTTGCTTCAGTAGTGTCATATGAGGTTCTGCCCAGAGACTATTATTATTATTTTTTTTTTCGCAAGCCCATCTTGCTGACTCGCTAGGCCTTGCTAATTTGCCAAAGTTTGTGTACTTGAATATCTCTAGCTAGGATAGGCCATTATGGGTTCGGTTTGCCCTGAAGTTACAAATGCTGTTGTGAATGAATTAATCTTTGCGCCTCTTGTGGCAAATTATTGCATACTTTTAGGAGCACGTACTCTCATTGCGGCATATATATGCTTTTTTATATGTGTATTTGTCATATGAGCG

*UBQ(2)*

ACGTCTTTGGCTCTTTGCTCACAGGTTCGAGCAGCAGGCATAGAAGACAACGGTGAGAAGAATTGCGAGTCTTCCCTTCTTCTGAACAACAAGCCTTGCCTTCAAGTACTTCAATCTCCATACTAAACTCCACAAAGAGGAGAAACGGAGAAAGAGAAAACCCAAGCAAACCCATCAAATTCTTCTTCAGATTGTTTGGGTGAAAACAAGAACATAAAAAGGGAATTCAGGGGAAAGGTGATTGAGAATGTTTTTCTCAGGCGATCCATCTACCCGGAAGCGAGTAGATTTGGGGGGTCGGAGTTCGAAGGAGAGAGACAGGCAAAAGCTTTTGGAGCAAACTCGGTTAGAGCGGAATCGTAGACTCTTTCAGCGCCAGCAGAACTCTGCTGCTACCAAAATTCAGAAATGCTTCAGAGCTTGGAGGGCTCTGCAAGCTGAACGTCACAATGTGCGGGAGGAGTTTTTTGTCAAATATGGAAAGCGCTGCCAGAATGTTGGTCGACATTGTTTTGCTCCAAATTCAGAGTTCCTCCGTGAGCTGCTTTTTTTCTTCAATCCAAAACACCAGGACGATCTTTTTGCTCTAGCGGAGGCTTGTCAACTACTTCAGCATTTTGGTCAAGACAATGGGGATCTAATCAACCTGTTTGCAGACATGGATTATTCATCAAATCATAGTCTAGTAGATTACAGGGTCAAGCAACTTGCTTATGCTTGCATTCGTGCTGTTCATGCAAATAGAAATCAGTTGAAGGGACCAATTTTCCTGTCACATGCAAATTCACAGATTGCAACAATTTTTCTGGGAGCTGTGAACAAGTTGATTGATCCCAAACTTCCATGGGCTTGCACTGTAGTTGGCTATCTTTCGCGAAGAAATACTTTTGCTTTGTTCAGAGACATAAGTCTGAGCACCAAGGAAAGTTTGAAGACTGGAAATTTAATAGTGGTCTCTTCCCTGGAGCAAGTGCTGGCCTGTATAATTCCGCATGTCGGTGAGAGCCCCTGTGTCTGTGAAAATGAAGACCAACAATGGAACTTTTCGTCCCAGATTCTTACAATACCCTTCTTGTGGCAAATTTTCCCGAAGCTAAAAGAGGTGTTTGCCAGGCATGATTTAAGCAAACATTATATGCACCAGATGACACTGTGCATACAGAGCCAGAGTAATGTTCTGCCAAATGACGTCTCACCCGAATATCCCAGTTATGCTTGTCTTCTTGGGAATGTATTAGAAGCTGCTTCTGTTGCTTTATCGCAGGCTGGTGGCTCATTTGAGATGGCCGTTGATCTAGCAGAAGTGGCGACATTTTTATTAGAGATGCTTCCACCTCTGAGGTCATTCAGTAGAGAAGTTAAAGAGAATTTTACGATAGAGGATGATGAAATGTCTCCTGTGGAAGAGTACAATGATAATCTGTTAAACGAGGATTTGGAAACACAAATATCTAATGCAATAGACCCACGTTTTCTTCTCCACTTGACGAATGTTTTGTTTGGAGGGATTTCACCGTTGAGTACGACAGATGATGTTCCTGATGACAAAGAAGTGATGGCTATAGGAGCGGCTTGTTGTTTCTTGCATGTCACTTTCAACACATTGCCTCTTGAACGCATAATGACCATCCTAGCCTACCGAACATCACTTGTTCCTGTTTTGTGGAATTTTATGAAACGTTGCCATGAGACTCAGAAATGGTCATTGCTGGCTGAGCGATTGTCTTACTTGTCGGAAGGTGCACCTGGTTGGCTGTTACCTCTAGCTGTATTCTGTCCTGTATATAAGCACATGCTTATGATTGTTGATAATGAAGAGTTCTATGAGCAGGAGAAACCCCTAGCACTCAAGGATATCAGATGCTTGATCATCATTCTAAGACAGGCATTGTGGCAGCTGCTGTGGGTAAATCCTGTGACACCATTGAACTCAGGCAAGTCTACTGCTAGGAGCTCAAGTAAGAAGAAGCGCCCATTAGAATTTCTGCAGCATAGGGTCAGTGTTGTTGCCGCTGAGCTTCTGTCGCAGTTGCAAGATTGGAACAATAGACGGCAGTTTGCACCTTCGAGTGACTTCCATGCAGATGGTGTGAATGAATATTTTATTTCTCAGGGTATGCAGGAGAACACTAGAGCTCGTGAAATACTAAAGCAAGCTCCATTCCTGGTCCCATTTACGAGCAGGGTTAAAGTATTTGCTTCTCAACTTGCTGCTGCTAGAAAAAGGGATGGTTCTCATGCCGTTTTTACTAGGAATAGACTCAAAATAAGGCGAGGGCACATCTTGGAGGATGCTTTCAACCAGTTGAGCACATTGTCAGAAGATGACCTTCGTGGCCCAATTCGTGTCACATTTGTCAATGAACTTGGGGTTGAAGAGGCTGGAATTGACGGTGGTGGCATTTTTAAAGATTTTATGGAGAACATCACTCGAGCTGCTTTTGATATACAATACGGATTGTTTAAGGAAACAGCTGATCATCTCCTCTACCCTAACCCTGGATCAGGACTAGTCCATGAGCGGCATCTTCAGTTCTTCCATTTTCTTGGAACAATTCTTGCAAAAGCAATGTTCGAGGGAATTCTTGTGGATATACCATTTGCGACCTTTTTCTTGAGCAAACTGAAACAAAAGCACAACTATTTGAATGACTTGCCTTCATTGGATCCAGAATTGTATCGCCACCTTATCTTTTTGAAGCATTATCAAGGTGACATTTCAGACTTGGAGTTGTACTTCGTCATTGTAAATAATGAATATGGGGAGCAATCAGAAGAAGAATTGATCCCAGGAGGAAAAAATATACGTGTTACGAATGACAATGTGATCCCTTTTATACATCTTGTAGCTAACCACCGCTTAAATTATCAGATACGCCAGCAGAGTTCTCATTTCCTTCGAGGATTTCAGCAGCTTGTACGGAAAGATTGGATTGATATGTTTAATGAACATGAACTTCAGATTCTGATATCGGGATCACTTGATGGTTTGGATATTGATGACCTACGTTCTCATTCGCAGTACTCAGGTGGATATCATGCAGAGCACTATGTTATCGAAATGTTCTGGGAAGTTCTCAAGGGCTTTTCTCAGGAAAATCAGATGAAATTCCTCAAATTTGTCACTGGGTGCTCTCGTGGACCATTGCTGGGATTTAAACACCTGGAGCCTCAATTTTGTATACAGAGAGCTGCTGGTAGTGCTTCAGAAGAAGCTCTTGATCGGTTACCTACGTCAGCGACTTGCATGAATCTGCTGAAGCTTCCACCATATAGAAGCAAGGAGCAACTGGAGAGAAAATTGCTCTATGCTATAAATTCAGAGGCAGGCTTTGACCTGAGTTGATAAAATTGCAAGTGTATGGGCTACTCCATGTATCTCACTTCATGTGGCGATCAGGATACTTCAATCTCCAGAGGTTGAATGTTCATAAATTCTCATCTACCTGCAGATCCAGCCCTGACGACAATAATTTATCTGAATTCAGAGGCATCGTTATCCAGCCACATTCCTGGAGGGTGAGATGACATTGCAAAAACGCTCCCAAGAGCCACTGTAGATATTTATGTATATCACCAGCTTTGCTGATTTACTTGTACGTGTAAATGTCTGAGGTAAAAATGCTCAGTACATTCAACTTCTGAAAATGTTACATGTTGAGGGTGATAGCGCTCCAATTTCAGTTCTTGTAACATCATTTCCAGTTGAATTTAACAGTTGAAAGTGGCAATATTTGAAAACATCCCTTTGGGCATTGAAGGGATGATTGCAGAGGCACGAACTCTGCAAAATTGTAAAAACAAGTATTTTTTCCCTTGCCTGAAGGTAAAAGGGACGAGTGCCCTTGTTTTTGCAACGTTCAATGGAGTTGCGATTATGGCTGTTACTGCAGATTGTGCTGTTCATCCTACATTCAACTATTACTTGGGTTATGGCTGCCCAATGGTATCATGCATCTTAGAAGGCTTGGGTTAGGAATAAGGTTGTATTTCTGTGATATTCTTTGGGACCGTATAGACTGAAAACTGATGAGTATTGAGCATCTGG

*UBQ(3)*

TTTTAAACAGAAGTCCTTCATCAGTTTGGTTTTGGGATTGATATGATAATTAGCAATCCCTTGGGTCCTCAAGCATATATTTGCATGATGTACAATTCAATCTGTGCGAATTACACATAATTTACGCATCATACTTCGGTTTTCTTTTGTTGTGCTACAAAAACATTACTGATTTTGACAAGGTATGTATGTCAAATCTTTCAGAATACACTGACTGTAACTGAGGTGGAGAAGATCAGAGCGAAAACACTACCACAAAATGCTCCACCCACTCAAAGCAGGACATGAACAGTGTACCCAATAAGGATGTTATTTCATCCAATAGTGTGCTAAGTACCATGGCTCAGCAAAAGCTCACAACTATAACCTTCGGATCCAATTTAGTACAGCTTGACATATACCATCTTCTTCTCAACCCCATCGGCAATAGTAGTTTCTCCCATTCCCCCCAATACCAAGTCAACCGCAAATCGATCAACCTGTGCTCAAGAAAGTTCAAACCCTGCATTGGAGTTGATTGCATACAGAAGCTTTGTCCTCAACGTACTAGCACGTTTATAGGTAGGAAGCTTCAGAGTATTGTAGCAGGTCGAAGCTGAGGGAAGCCGTTCCACATCCTGTCCTCCCATTGCTGCCCAAAGAGGCACATCACATGCAACCTTATGAATGGTAAAGGTTGGTTGCAAATGTTTAAAACCAAGTAGTGGAGCTCGTGAACAACTTGTTACGAACTTGAGGAGCGCACACCGTTCTTTTGGCTCAAAAGCTGCCATAACCTCCCAAAAGAGTTTGACTGTTCGGCTTCCTTCAGAGTATCCACCAGTATATCGGGTATTTTTTCTTAGATCATCAACTTCAATGTCATGATTTCCACCTGAAAGCAACTGATTAACTTCACTAGCATTAAACAGCTTCAGCCAGGATGGTGAAATAAGATCGGTTACTCCTCTGTAGAATGCAGTTGATAAAGGTAGCATCTGCCGATTGAGTTTATAATCTGCAACAGCATGAATGTATTGTAATTTGTTTTCATTTGTCACACTGATATCTTTGCCCCCAGATTTAAGCTCAATGACATGCCTCTTGCCTAACCACTCCTCTATAATAGTAAAATCCAATGAAAGTTCAGTAATATCACCACCATAACTCTTGACATACAATAGGTTCCGGTACAGCTCCGGATCAAGAGCAGAGAGTTCATCAAGAAAGCAATAACGGCCCAAAAGCTTTTGCACGAACACATGAGAAAAAGAATAATCCAGTAAGATTCCTTCATATAGAGCTTTTCCAACAATTCTTCCGATGAATTCAATCATCTGAATACCATTTTCTAGAAATCTTGCAGCAGTATTGGGGATTAACAGTCTTTCCGAAGTTGAGGTCTGACAGAACAGTCCATACTGTGGGTCAAATGCTGCCTTCGTGATGTCAGTTAAAAATTCCTTGGATAAACCCCCATAATCCAGTCCAGCCTCTCGAAGGCCACATTCACTGACAAATGAAACATGTATGGATGATTTCAACCTTGATCCCAGAGAATTTAATTGCTGAAAGCCATCCTCAACCAAATAACCCCTGCGAACTACTATCTCAATTGATCGCTCGCCAGATACATTCAGTTCACCAGCCATCCGGCGAGAGGTCTTGTCCATCTTGATGAACTCTCTGAATATCTGGACCCTTTCTTCAAAAGGATATACATGAGGAGTAATAGTTATAAATGAACCCATGCTTGGAAGGATCAAGGAACCATCTGATCTGACATCTGATAAAACTTCATGAGTCCTGGCAGCCAGTGCAATTGGGGGCCTACTTCTTCGGGCTGGTGAGAGCCACAAGGCAGGAGGACAAAACTTATGTCTGCAATCTCTTTCATACAACATTTGCAGACATCTAGCAGCAGCATCCATAAGATGTTTATCCCGGTGAGCAGTGTTGTGGAGCAAGCTGTTGTATACTAGTGTATTTAGCATCGATGCAATCCTCCTCTGCTGCTCCAACAAGAATGGCACCTGTTTCTCATAGAATTCTATATCATCAAGCACTAACAGTAGGTGTGCATATGTGGCACAAAACAAATGCATTAGGCATGAATTTTCTTTTGAAATGCCTTCAAGGCCACCTCTCAAAGATTCAACATCCCATACATCATCTACATTGCCTTTTTCATCGCTGCCTCTCGGCCGACTATCAATTGCATCAGGGTTAACTGTATCACTCTGTGATTTGCCACTGATTTTATGCAATACATTAACCCATTTAGAAGCCATATCTTTACTAGGATTTTTTTGTTTTTTTCTTAGAGACCGCATTAAATTTGTTCTCATACATCTCAGGACCACAGCGAGAATCATCTCCAGAAGGTTGAGCTGTTCCTGGAAAAAGAGAGCACTCCAGAGCTCCCCAAAGGCTGGTCAGAAAGCCAGGAGTAAAAGACAGCATATTCAGAACAGGCAAGGACCCACCCATTGAATTTAAAACTGAGTACACTCTGATCATCTGAGAATAAAAACAAGCAATGTTGGCCAATTCCAACATTTTCATATGTTTTTTGTCCGCATATGACAGCAGAGAATCACTCTCATTTGCATAAGCATTGCTCTTTAATGCATCCATAAGTTTTGCAAGATGCCACTGCTGACAAACAGGCCTTAGAAGGTCCATGTATCTCAAGGATTCAAAAGTTGTTTCCCTCAAAAAGACAAATGTCTTGACCGGCACAACAGAGCTTCCTTCATCTTCCTCTAAATTTTGACATCCCTTTCGCACACACTCATTGCTCTCAATCCCACCTAACAATCCCCTCGCAAGGATATTGAGTGCATGAACATATCCAGGAATATCCACACCATCAGTAAACCTGCTAGGTTCAATTGATGCATTCTCACTCTCTGTTACCAGACAGATCATGTTTGCAAGAGCCCAACCAACAGAAGGCATATGATTACTAGAGGAAGCTTTTGAGAGATCTATCCTTGAATATTCCGTCAAAATTTTTTTCATTCAAGATCTGCCATCACCAGGAGAGTCTGAAAGCAGGGGCCCAGAGTGGACATATGCCTCAAAGCACGTAGCAGTACTGCCGGTATACGTTGAACAAGCCAAGGTACTGTAAGTATGAACAGGCAATACTGCTCAGCAGCACATTGCGAGTTGAAAAATAAAGAATCCTTCTCATCTAATTTGAATGCCTGAAAGGGACGCAAAGCCAGAGTTATTGCACTTGCAGTAACCAAGAACTTATCATCCTTATGCACAGCCCCTGGAGAAGAAAAAGAATCGTTCAGCTTATTTAAGTATCTTCCAATGGATCCATATAGGCCACTTTTATCACTTCCCATAAATTTGATTATATCTTTCACAGCTCTCTCTGCATCCTCACAATATTCATCAGATATGCACTTCCATGCCTTTGAATCAGTGAAGATGATCATCAAATGCATTAGCGGAGTAGCAACAGCAACAACATCACCACTCGCTGGGGAATAACAATCATCATTACCAGCAAGTATAAATGAGCATAACGAGACTAGCTTCTGAACTTGGTAAGCCCACATCTTTCGCTCATCAGGCGTGCCAATTGCCAAAGAGCAGAAGTTTCTATCTGGGTCAACTGAATTGATGTTTTCCAACAGAATAATGAAGCAGCTGCGCATGCAGTTTACAGCCTGAGTACAAATGGCACATCGGCTGGTTGATAAATTTGTAATGAAAAACAGAAAGGGCCTCAATAAACGAGAGGAAATCCATGTTTTATCTATCAACCCAGAACCATCTCTTAGTACAACGTCCCATTCTTCTTGAAGCTTGAGGGCCGCTGTCTTTGTCGCATAATAGCGTCGCCAAACCCTCTGGATGAAAAGAGCAGCGGCGTTGGCTCGCCTAGCAAAATTACGGAGCTCTCTTTCCCGGGAGACCCTGTCAAGAAGGGCATCCCTTGTGATTTCCCGGGCACTCGCTCCTCTGAGAGACACCTGATTTTTCCAGGATTCATCCATGAAATCAAGCGATCATCTCCGATACAGAGTCGTCCTCGGTTGTTGGCGCCGTCCGTCTACTTGCTGACTGCTCA
