# Supplementary material for: Identification of reliable reference genes for quantitative real-time PCR normalization in pitaya
Source: Plant Methods. 2019 Jul 8;15:70. doi: 10.1186/s13007-019-0455-3 (PMC6613322; doi:10.1186/s13007-019-0455-3)
Supplement: Supplementary file 3 — Additional file 3: Table S2. Sequence analyses of thirty-nine reference genes. [file 13007_2019_455_MOESM3_ESM.doc]

**Additional file 3: Table S2 Sequence analyses of 39 reference genes**

| Gene Name | Length of cDNA fragments (bp) | Accession No. of similar genes | Identity (%) |
| --- | --- | --- | --- |
| *18S rRNA* | 234 | KY860919.1 | 100 |
| *Actin(1)* | 1082 | MF356257.1 | 98 |
| *Actin(2)* | 482 | XM_010687471.2 | 85 |
| *Actin(3)* | 718 | XM_021893846.1 | 89 |
| *Actin(4)* | 804 | XM_021904392.1 | 88 |
| *CYP(1)* | 499 | XR_002066190.1 | 83 |
| *CYP(2)* | 764 | XM_021914119.1 | 83 |
| *EF1-α(1)* | 3305 | XM_021919865.1 | 86 |
| *EF1-α(2)* | 1698 | XM_021987711.1 | 81 |
| *EF1-α(3)* | 638 | XM_021986216.1 | 87 |
| *EF1-α(4)* | 5304 | XM_010674818.2 | 83 |
| *EF1-α(5)* | 2418 | KY056733.1 | 91 |
| *eIF(1)* | 1463 | XM_021866570.1 | 86 |
| *eIF(2)* | 1504 | XM_021912280.1 | 83 |
| *eIF(3)* | 3522 | XM_021918090.1 | 82 |
| *eIF(4)* | 5189 | XM_021906622.1 | 83 |
| *eIF(5)* | 4127 | XM_021871814.1 | 80 |
| *GAPDH* | 1845 | MF356269.1 | 96 |
| *histone(1)* | 601 | XM_013846585.2 | 86 |
| *histone(2)* | 506 | XM_021881080.1 | 92 |
| *histone(3)* | 992 | KC415067.1 | 92 |
| *histone(4)* | 733 | XM_019846886.1 | 82 |
| *histone(5)* | 2184 | XM_010697425.2 | 82 |
| *TATA(1)* | 1551 | MF356277.1 | 99 |
| *TATA(2)* | 768 | KY056735.1 | 86 |
| *TATA(3)* | 1280 | XM_021870997.1 | 82 |
| *TUA(1)* | 914 | MF356280.1 | 93 |
| *TUA(2)* | 2173 | XM_010695516.2 | 87 |
| *TUB(1)* | 2268 | MF356281.1 | 96 |
| *TUB(2)* | 2270 | XM_010100239.2 | 84 |
| *UBC(1)* | 1026 | XM_022011354.1 | 89 |
| *UBC(2)* | 1032 | MF356282.1 | 96 |
| *UBC(3)* | 803 | XM_010671803.2 | 86 |
| *UBC(4)* | 2111 | XM_010690708.2 | 81 |
| *UBC(5)* | 1531 | XM_016864885.1 | 83 |
| *UBC(6)* | 3394 | XM_021980180.1 | 86 |
| *UBQ(1)* | 5246 | XM_021918796.1 | 84 |
| *UBQ(2)* | 4048 | XM_010674296.2 | 81 |
| *UBQ(3)* | 4072 | MF356264.1 | 99 |
