# Supplementary material for: Identification of reliable reference genes for quantitative real-time PCR normalization in pitaya
Source: Plant Methods. 2019 Jul 8;15:70. doi: 10.1186/s13007-019-0455-3 (PMC6613322; doi:10.1186/s13007-019-0455-3)
Supplement: Supplementary file 4 — Additional file 4: Fig. S2. Melt curve analyses of thirty-nine reference genes from eight different tissues (including roots, stems, flowers, and fruits) of Hylocereus. [file 13007_2019_455_MOESM4_ESM.docx]

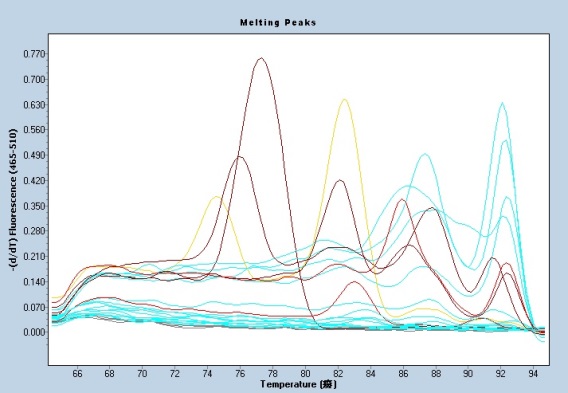

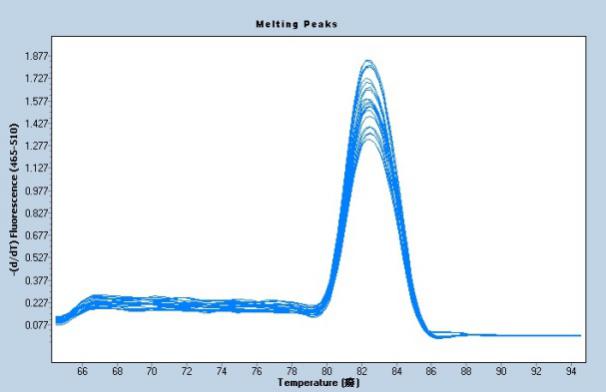


*18S rRNA* *Actin(1)*


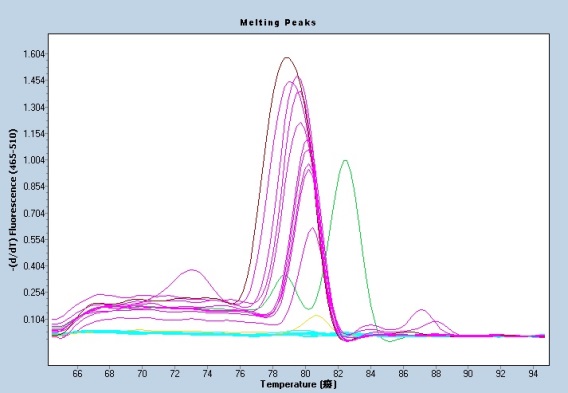

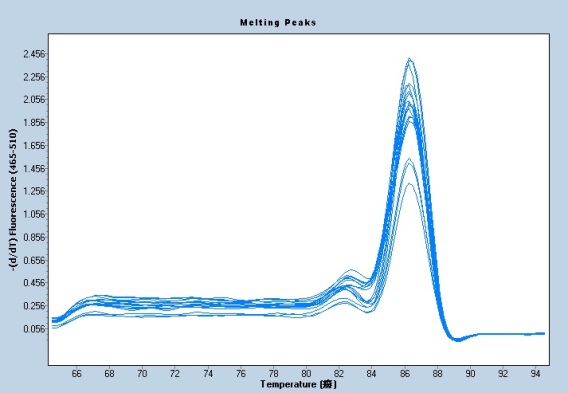


*Actin(2)* *Actin(3)*


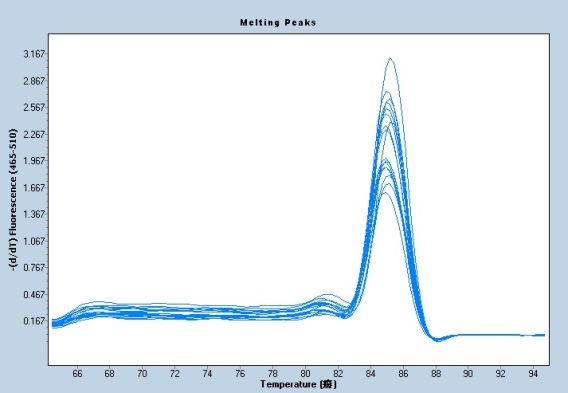

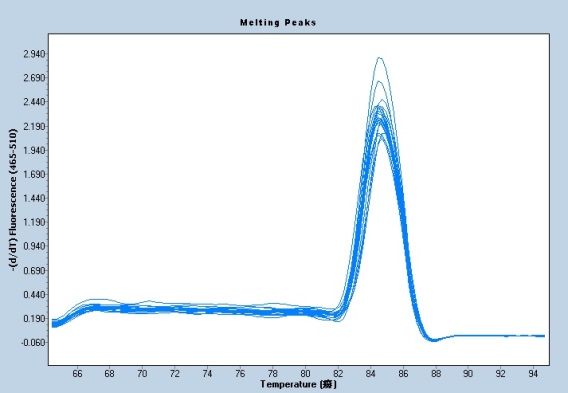


*Actin(4)* *CYP(1)*


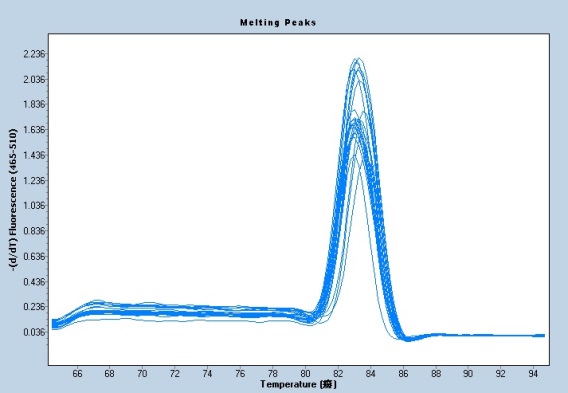

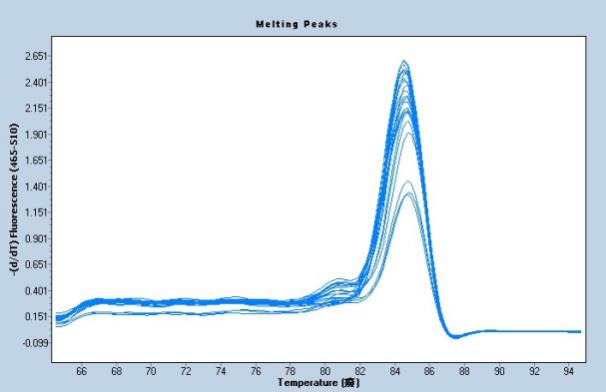


*CYP(2)* *EF1-α(1)*


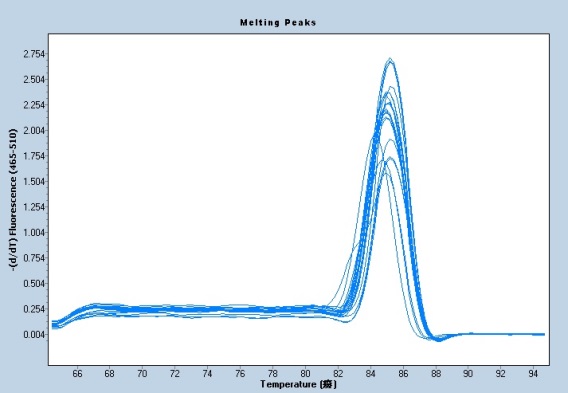

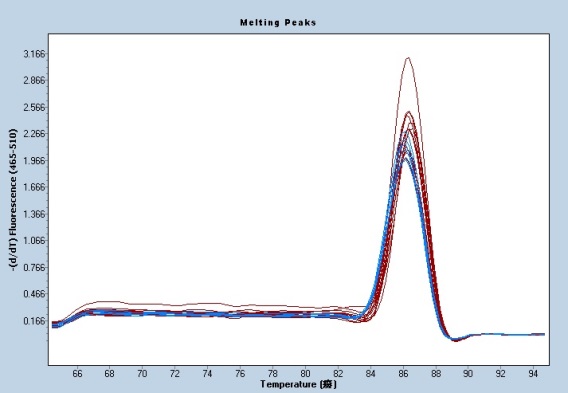


*EF1-α(2)* *EF1-α(3)*


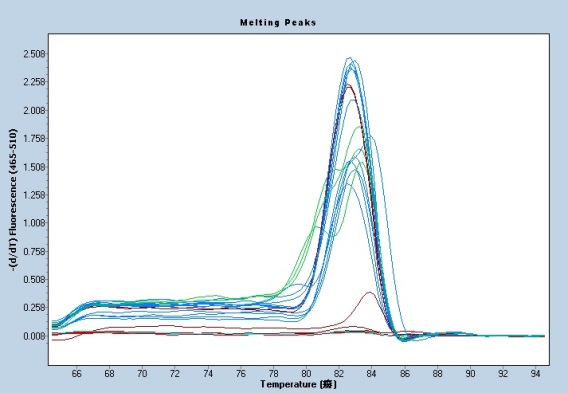

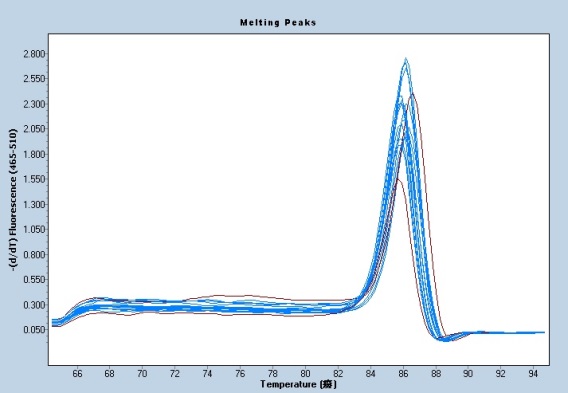


*EF1-α(4)* *EF1-α(5)*


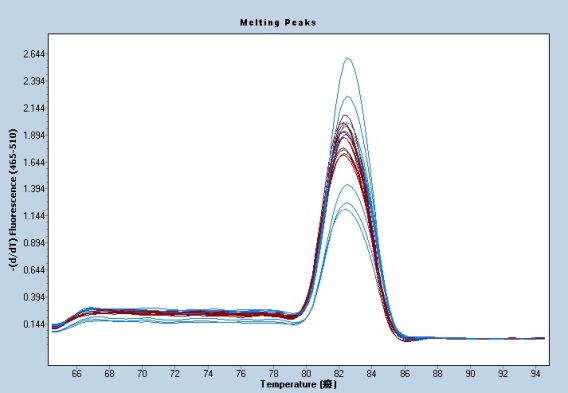

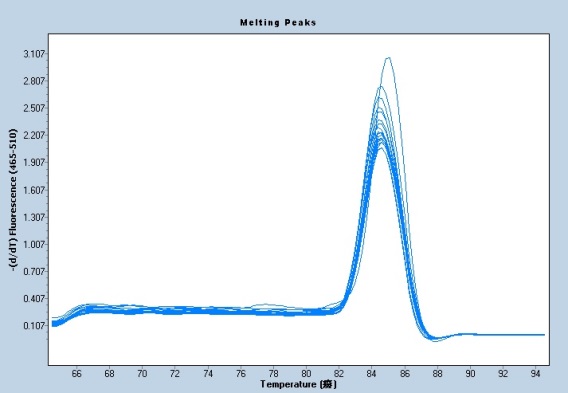


*eIF(1)* *eIF(2)*


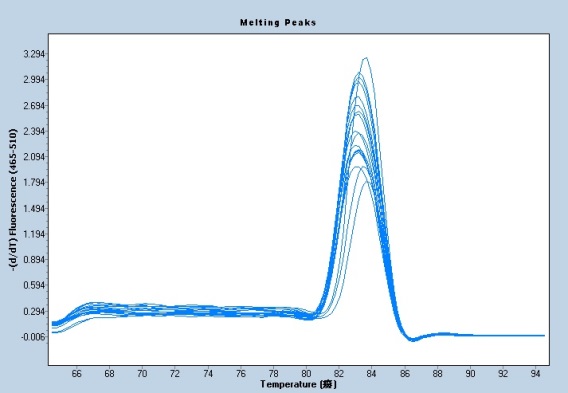

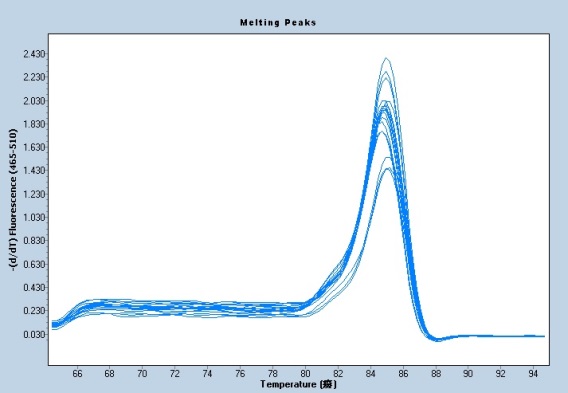


*eIF(3)* *eIF(4)*


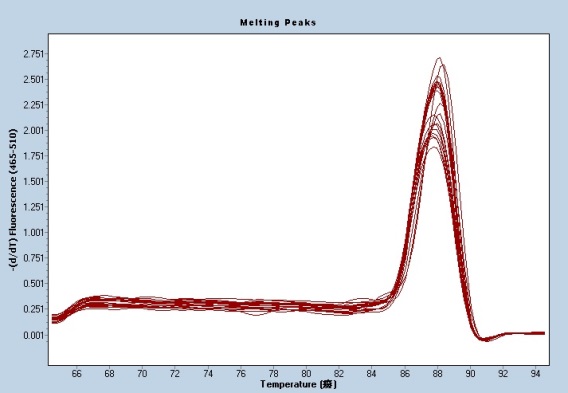

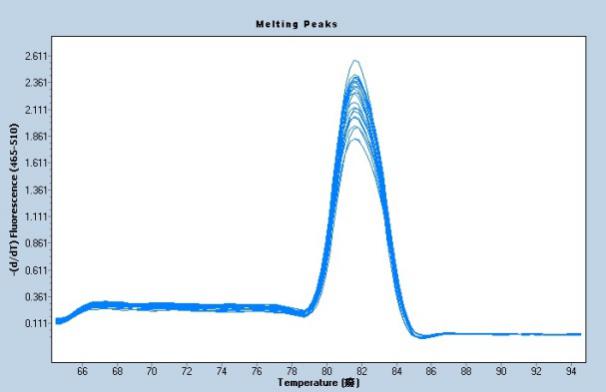


*eIF(5)* *GAPDH*


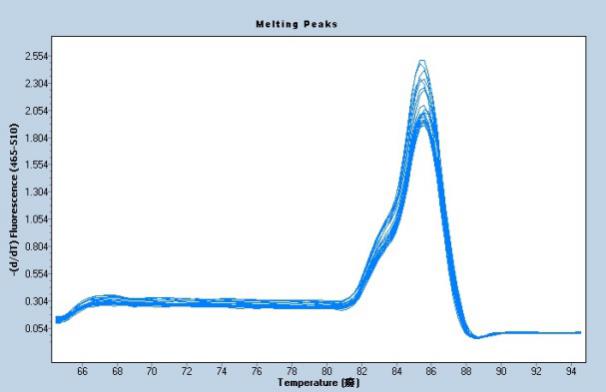

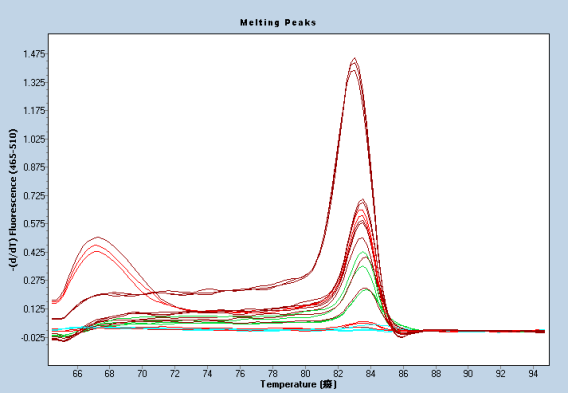


*histone(1)* *histone(2)*


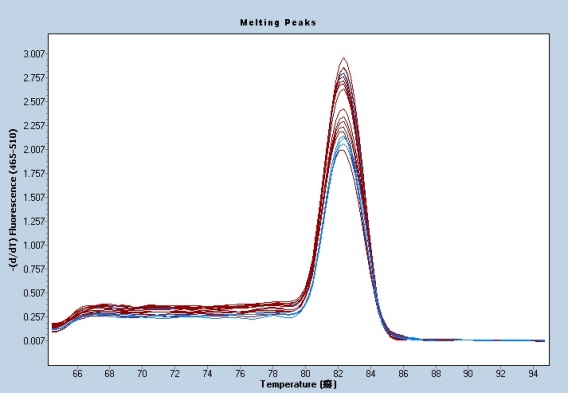

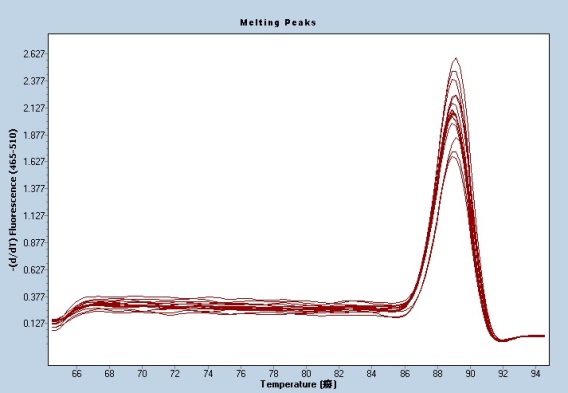


*histone(3)* *histone(4)*


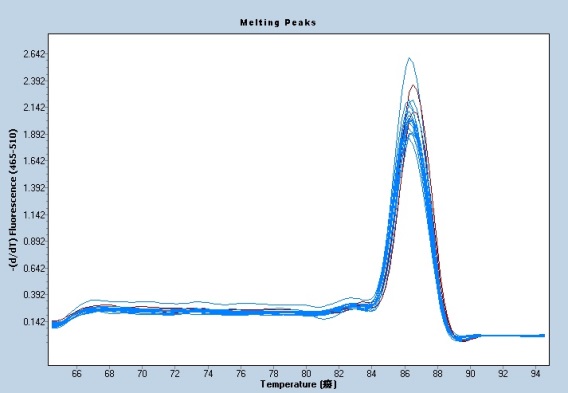

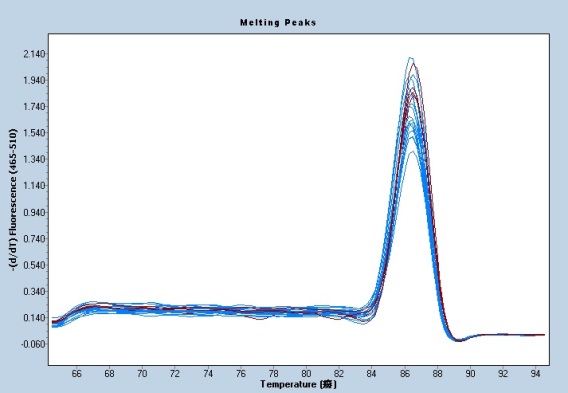


*histone(5)* *TATA(1)*


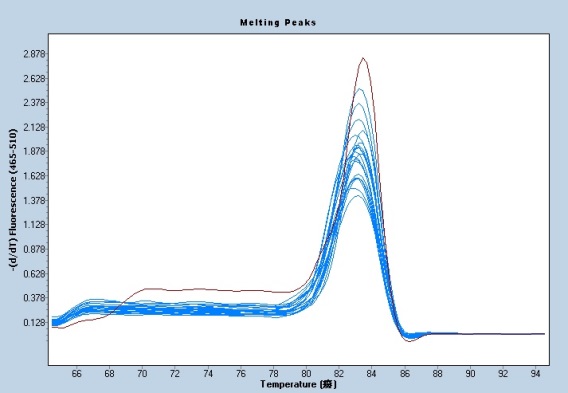

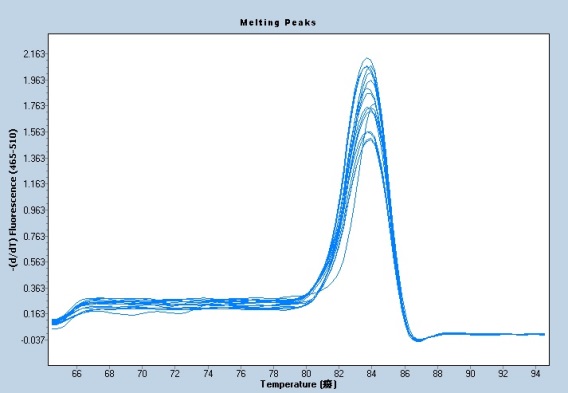


*TATA(2)* *TATA(3)*


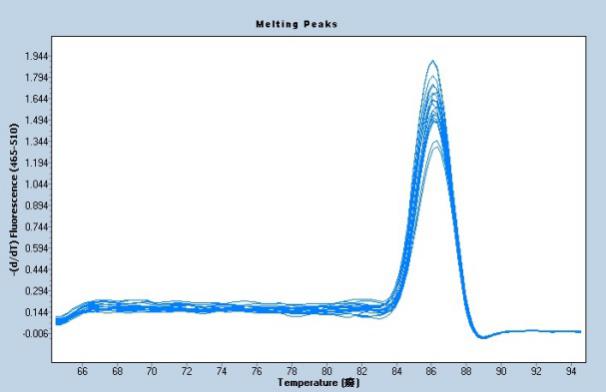

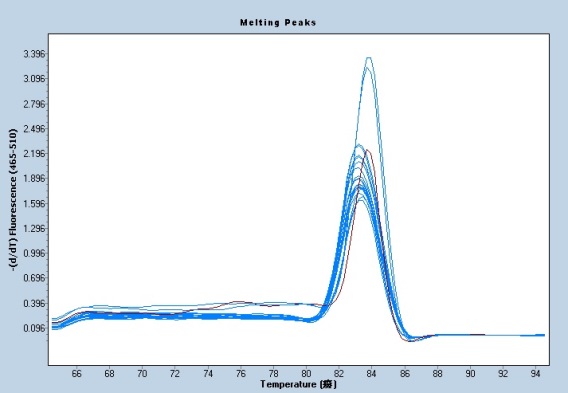


*TUA(1)* *TUA(2)*


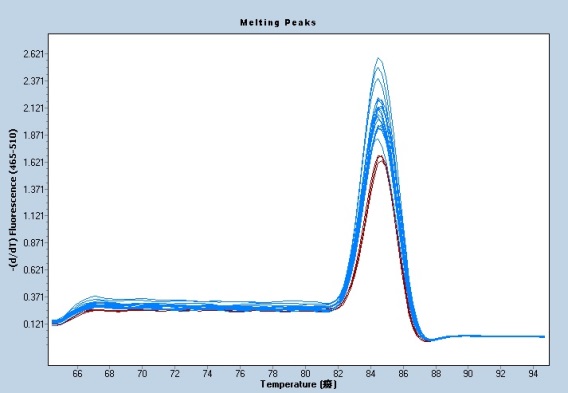

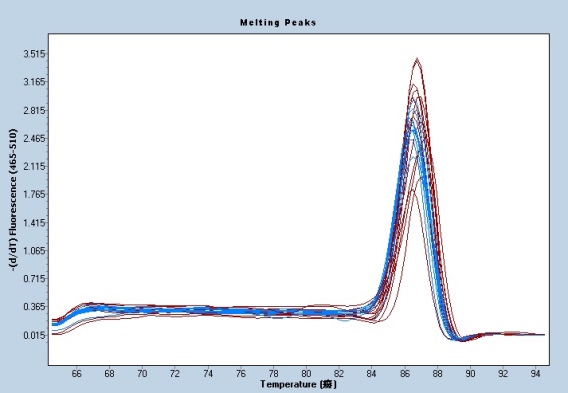


*TUB(1)* *TUB(2)*


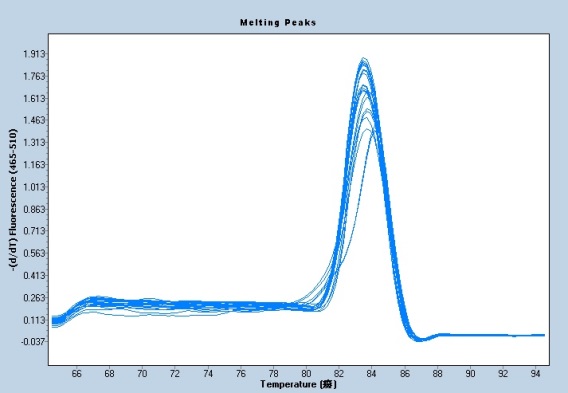

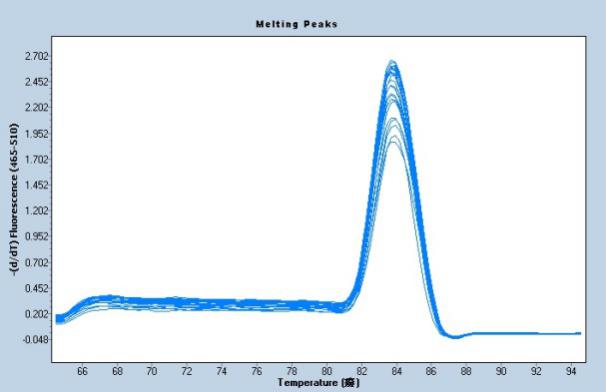


*UBC(1)* *UBC(2)*


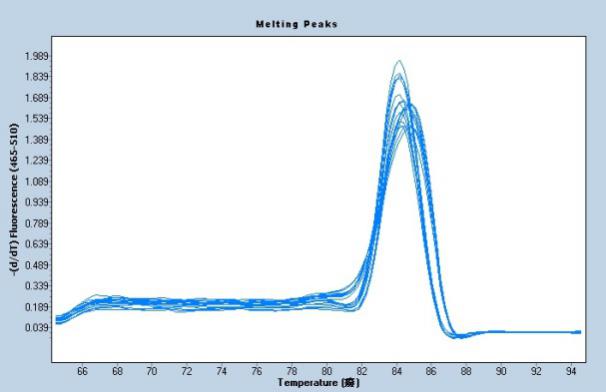

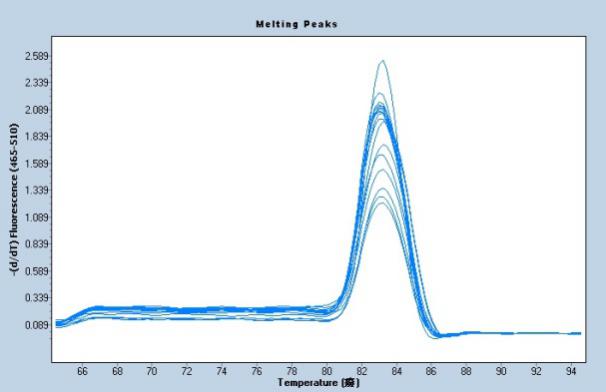


*UBC(3)* *UBC(4)*


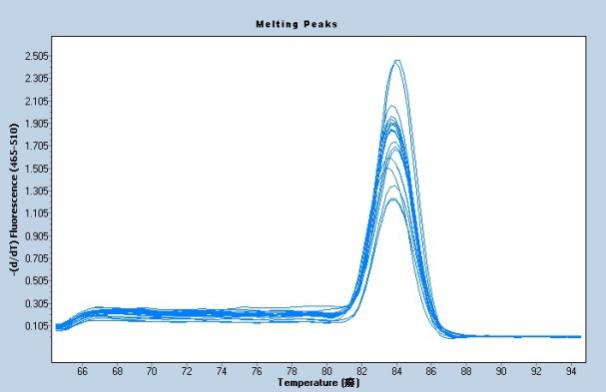

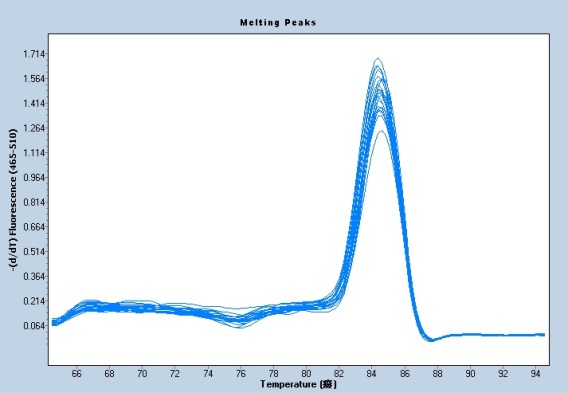


*UBC(5)* *UBC(6)*


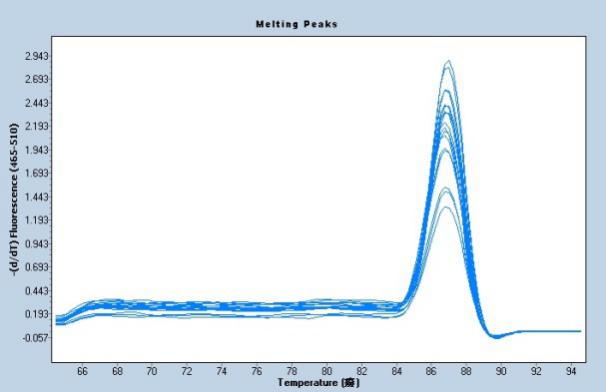

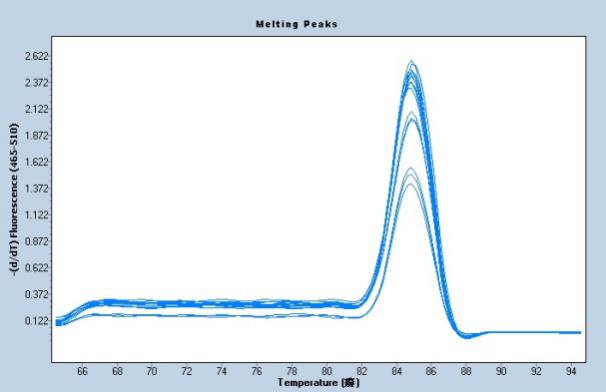


*UBQ(1)* *UBQ(2)*


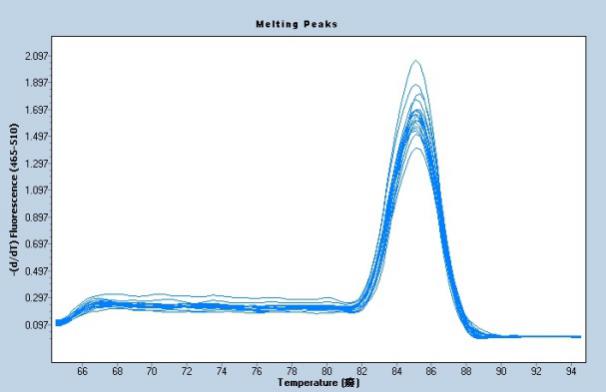


*UBQ(3)*

**Additional file 4: Fig. S2. Melt curve analyses of thirty-nine reference genes from eight different tissues (including roots, stems, flowers, and fruits) of *Hylocereus***
