# Supplementary material for: Identification of reliable reference genes for quantitative real-time PCR normalization in pitaya
Source: Plant Methods. 2019 Jul 8;15:70. doi: 10.1186/s13007-019-0455-3 (PMC6613322; doi:10.1186/s13007-019-0455-3)
Supplement: Supplementary file 5 — Additional file 5: Fig. S3. Boxplot analyses of thirty-nine reference genes from eight different tissues (including roots, stems, flowers and fruits) of Hylocereus. The whisker caps show the distribution of the highest and lowest Ct values. The boxes indicate the first and third quartile, while the middle line marks the median. [file 13007_2019_455_MOESM5_ESM.docx]

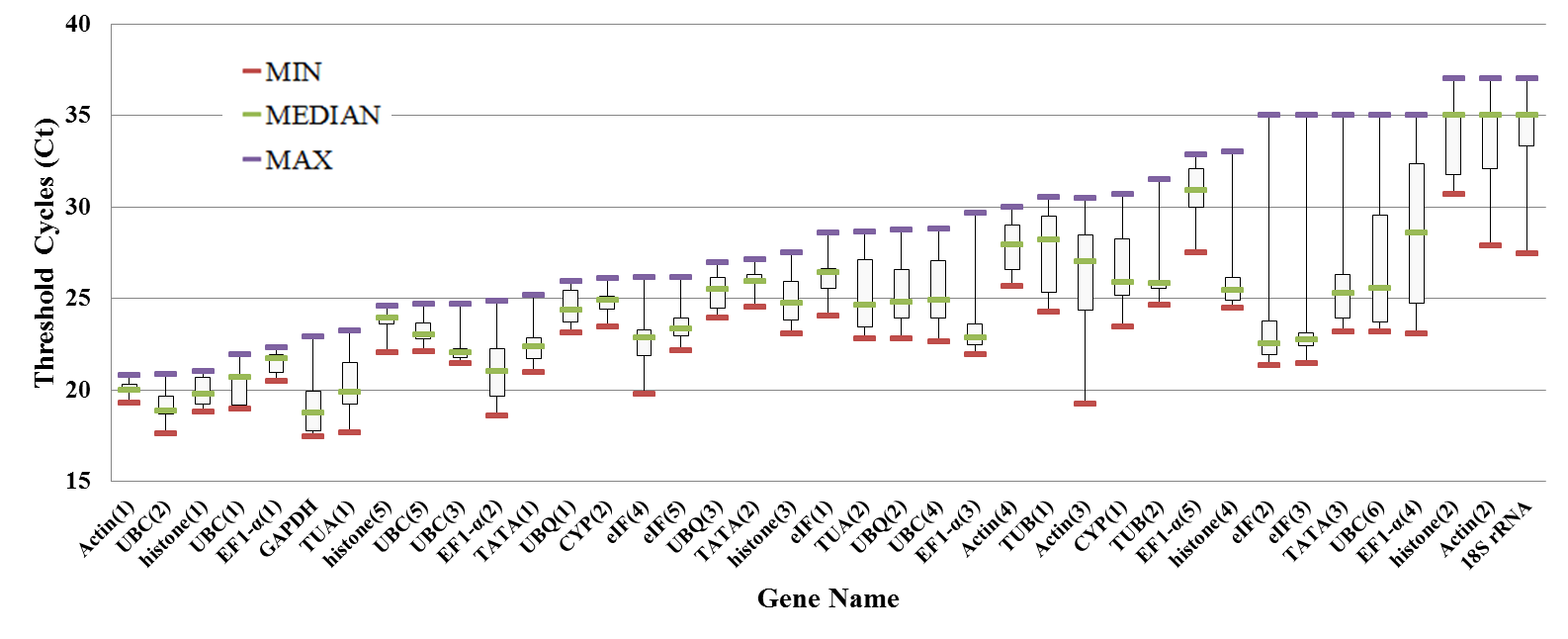


**Additional file 5: Fig. S3. Boxplot analyses of 39 reference genes from eight different tissues (including roots, stems, flowers and fruits) of *Hylocereus.*** The whisker caps show the distribution of the highest and lowest Ct values. The boxes indicate the first and third quartile, while the middle line marks the median.
