# Supplementary material for: Identification of reliable reference genes for quantitative real-time PCR normalization in pitaya
Source: Plant Methods. 2019 Jul 8;15:70. doi: 10.1186/s13007-019-0455-3 (PMC6613322; doi:10.1186/s13007-019-0455-3)
Supplement: Supplementary file 6 — Additional file 6: Fig. S4. Ct value of six candidate reference genes in different temperature stresses of pitaya cutting plantlets. [file 13007_2019_455_MOESM6_ESM.doc]

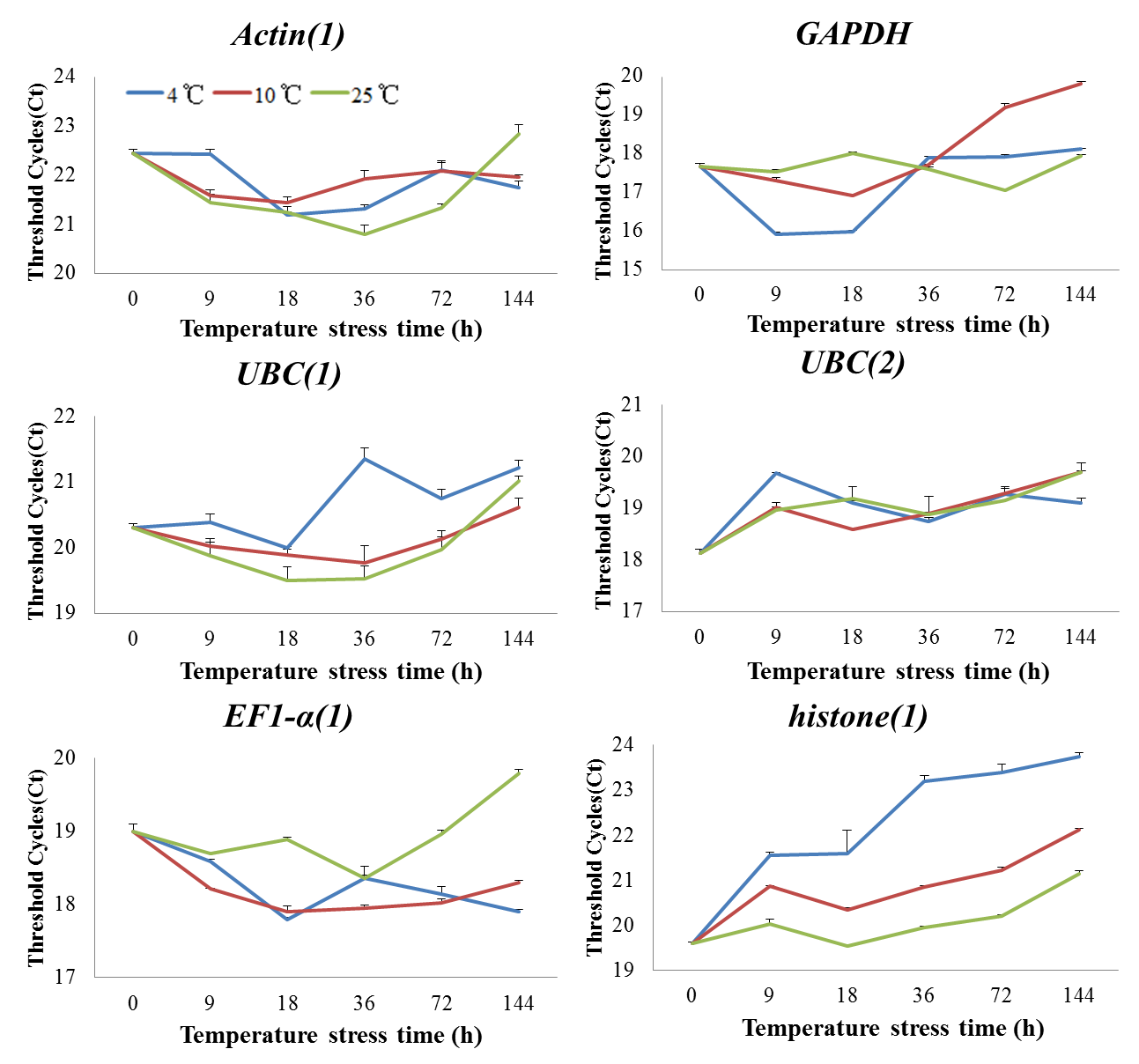


**Additional file 6: Fig. S4. Ct value of six candidate reference genes in different temperature stresses of pitaya cutting plantlets.**
