# Supplementary material for: Identification of reliable reference genes for quantitative real-time PCR normalization in pitaya
Source: Plant Methods. 2019 Jul 8;15:70. doi: 10.1186/s13007-019-0455-3 (PMC6613322; doi:10.1186/s13007-019-0455-3)
Supplement: Supplementary file 7 — Additional file 7: Table S3. The coefficient of variance (CV) of the six candidate reference genes using BestKeeper algorithm. [file 13007_2019_455_MOESM7_ESM.docx]

**Additional file 7: Table S3. The coefficient of variance (CV) of the six candidate reference genes using BestKeeper algorithm**

|  | *Actin(1)* | *GAPDH* | *UBC(1)* | *UBC(2)* | *EF1-α(1)* | *histone(1)* |
| --- | --- | --- | --- | --- | --- | --- |
| Fruits of Guanhuabai pitaya | 3.44% | 5.15% | 13.16% | 8.89% | 4.88% | 6.41% |
| Fruits of Guanhuahong pitaya | 3.35% | 5.60% | 8.16% | 8.70% | 4.36% | 6.14% |
| Tissues of Guanhuabai pitaya | 4.96% | 9.46% | 6.80% | 8.01% | 4.88% | 7.05% |
| Tissues of Guanhuahong pitaya | 4.40% | 5.97% | 8.28% | 15.61% | 7.37% | 8.99% |
| Temperature stress | 2.18% | 3.35% | 2.07% | 1.99% | 2.38% | 5.14% |
| Total | 4.71% | 7.07% | 9.44% | 11.44% | 6.00% | 7.74% |
